# Characterization of black oat root exudates in the presence of interspecific weed species and intraspecific neighbors, and their effects on root traits

**DOI:** 10.3389/fpls.2026.1729814

**Published:** 2026-03-25

**Authors:** Çağla Görkem Eroğlu, Alexandra A. Bennett, Teresa Steininger-Mairinger, Stephan Hann, Victor Patricio Rueda Ayala, Judith Wirth, Aurélie Gfeller

**Affiliations:** 1 Weed Science in Arable Crops, Plant Production Systems, Agroscope, Nyon, Switzerland; 2 Core Facility Mass Spectrometry, University of Natural Resources and Life Sciences, Vienna (BOKU), Vienna, Austria; 3 Department of Chemistry, Institute of Analytical Chemistry, University of Natural Resources and Life Sciences, Vienna (BOKU), Vienna, Austria

**Keywords:** *Alopecurus myosuroides* (blackgrass), *Amaranthus retroflexus* (redroot pigweed), *Avena strigosa* (black oat), weed, non-targeted metabolomics, plant-plant interactions, root exudates, root traits

## Abstract

**Introduction:**

Cover crops, like black oat, suppress weeds in multiple ways, e.g. by resource competition and/or root exudates, but very little is known about their root exudate composition.

**Methods:**

Root exudates from black oat interacting with intra- (black oat) and interspecific (redroot pigweed and blackgrass) neighbors were collected using split-root systems and analyzed with mass spectrometry. Changes in black oat root exudation patterns and root morphology were assessed. Moreover, collected exudates were applied to weeds to observe the effect on weed root traits.

**Results:**

Redroot pigweed root traits declined when grown with black oat, whereas blackgrass showed the opposite trend. Upon black oat root exudate application, pronounced effects on redroot pigweed root traits were observed, whereas there was no reaction in blackgrass. The proportionally more accumulated compounds in root exudates in response to neighbors were primarily organic oxygen compounds, most annotated as carbohydrates. In the presence of redroot pigweed, root exudation increased, with sugar sulphates and potential glycosylamines identified as priority compounds.

**Discussion:**

This study provides the first characterization of black oat root exudates and demonstrates the varying patterns of black oat root exudation when interacting with different neighbors and their influence on root traits.

## 1 Introduction

Weeds can cause substantial crop yield losses when not adequately managed (Oerke, 2006). In this context, increasing attention has been given to plant-plant interactions occurring within agroecosystems, particularly considering growing concerns regarding herbicide use. Among alternative, non-chemical weed management strategies, the use of weed-suppressive cover crops represents a promising approach. These crops play an important role in integrated weed management, reducing the emergence and growth of weeds through competition, allelopathic interactions and mulching. Black oat (*Avena strigosa* Schreb.) is widely recognised as one of the most effective species for this purpose across various agroecosystems (Gfeller et al., 2018b; Schappert et al., 2019; Cechin et al., 2022; Marquez et al., 2022). This is due to its rapid growth, strong competitive ability, high biomass production, and potential allelopathic effects. These characteristics make black oat a valuable component of sustainable and integrated weed management systems.

Previous field and pot trials have demonstrated the ability of black oat to significantly suppress the growth of the summer annual dicotyledonous weed redroot pigweed (*Amaranthus retroflexus*) (Gfeller et al., 2018b). However, in this initial study, which aimed to differentiate effects related to competition from those associated with allelopathy, only the aboveground biomass of redroot pigweed was measured. To gain more insight into the underlying mechanisms, this study focuses on the root traits of redroot pigweed in interaction with black oat. In addition to studying a dicotyledonous weed, we also examined the interactions between black oat and a monocotyledonous weed. We selected blackgrass (*Alopecurus myosuroides* Huds.), one of the most significant and troublesome winter annual weeds in European cereal production systems, which has been shown to respond to allelopathic wheat cultivars with reduced biomass (Bertholdsson, 2012).

Several studies indicate that allelopathy contributes to the weed-suppressive properties of black oat (*Avena strigosa*) through the release of chemical compounds via roots and root exudates (Gfeller et al., 2018b; Sturm et al., 2018; Reginatto et al., 2023), yet the mechanisms underlying these allelopathic effects remain incompletely understood. Root exudates comprise a wide range of primary and secondary metabolites, including sugars, amino acids, organic acids, phenolics, terpenoids, and alkaloids, which function as signaling molecules in plant–plant and plant–microbiome interactions (Badri and Vivanco, 2009; Zhalnina et al., 2018; Maurer et al., 2021; Mondal et al., 2023; Alahmad et al., 2024). Exudate composition and quantity vary with species, genotype, and environment, and can be reciprocally influenced by neighboring plants, creating a cycle of mutual chemical modulation (Gfeller et al., 2018a; Eroğlu et al., 2022; Upadhyay et al., 2022; Gunjal, 2023). Primary root metabolites, can shape root system architecture by promoting lateral root growth, elongation, or meristem depletion, thereby enhancing resource uptake (Malamy and Ryan, 2001; Canarini et al., 2019; Upadhyay et al., 2022). Some exudates act as allelochemicals that suppress or stimulate neighboring plants and influence soil processes and microbial communities (Inderjit and Duke, 2003; Bais et al., 2006).

For black oat, limited literature documents scopoletin as a potential secondary metabolite of interest (Pérez and Ormeño-Nuñez, 1991), while primary metabolites can be assumed given their universality across plants. However, the range of exudates relevant to plant–plant interactions remain poorly characterized in black oat. Studies across a wide range of plant species reported in the literature highlight multiple possible chemical classes that may contribute to these interactions, but the diversity of potential compounds makes the use of a focused target list an impractical approach. In this context, mass spectrometry-based non-targeted analysis (NTA) provides qualitative and relatively quantitative coverage across a broad range of analytes while limiting selection bias, enabling the annotation of both known and previously unrecognized exudates in the rhizosphere (Gertsman and Barshop, 2018). Accordingly, the developed NTA framework ensured coverage of the above highlighted classes while remaining open to the discovery of additional compounds that may influence weed responses. To ensure reproducibility and confidence in identified compounds, this framework applied established community standards for feature prioritization, statistical validation (Chanana et al., 2017; Kumar et al., 2018), compound identification (Schymanski et al., 2014), and data corroboration through molecular networking (Vaniya and Fiehn, 2015; Wang et al., 2016; Nothias et al., 2020).

This study aims to characterize neighbor-dependent shifts in black oat root exudation and their effect on two representative weeds. In particular, black oat (*Avena strigosa*) is hypothesized to (i) modify root traits of redroot pigweed (*Amaranthus retroflexus*) and blackgrass (*Alopecurus myosuroides*) through both direct root interactions and indirect effects mediated by root exudates; (ii) adjust its own root traits and exudation patterns in response to interspecific and intraspecific neighbors; and (iii) increase the accumulation of specific root exudates in the presence of weed neighbors, potentially contributing to weed suppression.

To achieve this, the study used split-root systems, which had been previously established (Eroğlu et al., 2024), to grow plants and collect root exudates. A dual extraction method was used to collect exudates from the same system at different time intervals (Bennett et al., 2024). This approach sheds new light on black oat root exudation and its impact on the root characteristics of intraspecific and interspecific neighbors. It aims to lay the groundwork for future research into plant-plant interactions and their implications for sustainable weed management strategies.

## 2 Material and methods

### 2.1 Plant material and split-root set-up

**Figure 1:**
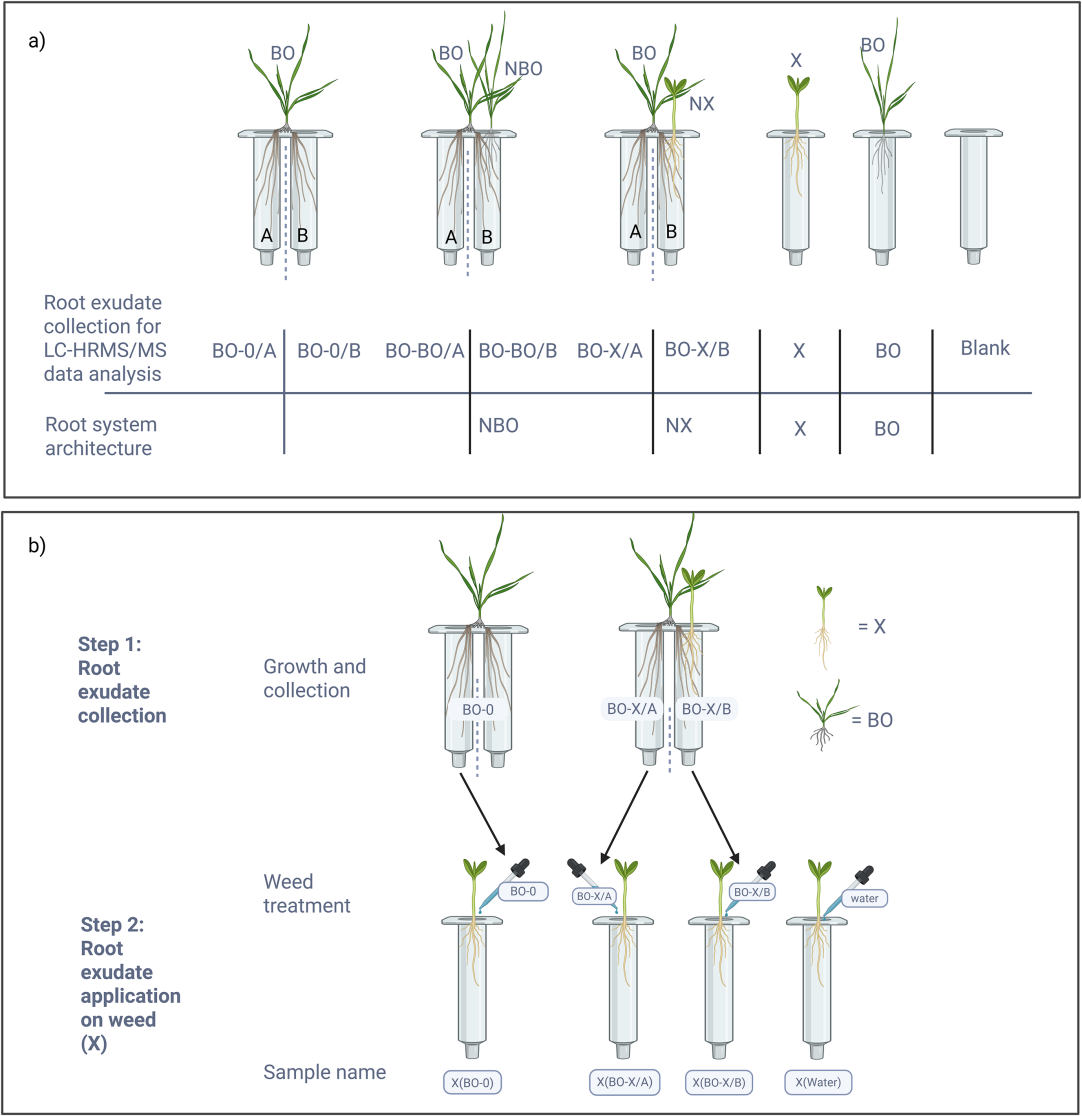
Experimental design for root exudate collection from black oat split-root systems and assessment of their effects on weed root system architecture. **(a)** Black oat (BO) plants were grown in split-root systems with roots distributed between two compartments (A and B). Plants were cultivated either without neighbors (BO-0), with a conspecific neighbor (BO-BO), or with a heterospecific weed neighbor (BO-X), where X represents redroot pigweed (P) or blackgrass (G). Non-split plants grown alone (BO and X) and blank cartridges containing glass beads only were included as controls. Root exudates were collected from compartment A of BO-0, BO-BO, and BO-X systems, as well as from single-root BO, X, and blank cartridges, for LC-HRMS/MS analysis. Root system architecture (RSA) of plants growing in compartment B was measured on the same day as exudate collection (NBO = black oat neighbor; NX = weed neighbor; BO = black oat grown alone; X = weed grown alone). **(b)** Root exudates collected from BO split-root systems were applied to weed seedlings to evaluate their effects on RSA. Plants were grown in glass beads and supplied with nutrient solution; nutrients were washed out with water 24 h prior to exudate collection. Exudates were collected from BO plants grown without neighbors (BO-0) and with weed neighbors (BO-X). Two types of BO-X exudates were obtained: BO-X/A, collected without direct root–root contact, and BO-X/B, collected with direct root–root contact. Collected exudates (3 mL day^-^¹) were applied to weed seedlings for 10 days, after which RSA was measured. Control plants received nanopure water. X corresponds to redroot pigweed (P) or blackgrass (G), depending on the experimental setup.

Five black oat (*Avena strigosa*, variety: Altesse) seeds were placed on Whatman filter papers moistened with half-strength Hoagland's solution (pH 5.8) in 120 x 120 x 15 mm Petri dishes. The Petri dishes were sealed, set vertically in a phytotron (25/20 °C day/night, 70% humidity) in the dark for three days, then exposed to a 16/8-hour light/dark cycle (200 μmol m^-^² s^-^¹). Five days after sowing (DAS), the seedlings were transferred to split-root systems, consisting of two joined Solid Phase Extraction (SPE) cartridges (60 mL, Bond Elut, Agilent) covered with black film, and filled with glass beads (250-400 µm, Guyson SA). Black oat (BO) roots were split to be equally placed in cartridges A and B (**Figure 1a**). The A compartments contained only split BO roots. In the B compartments there was either no neighbor (BO/0-B), a homospecific BO neighbor (BO/BO-B), or a weed neighbor (BO/X-B). Neighbor plants were sown on the day of the split-root establishment (**Figure 1a**). Two distinct weed neighbor treatment groups were included, denoted as X. Specifically, two subsets of X were created: 'P set', where redroot pigweed (P) serves as the neighboring weed species, and 'G set', where blackgrass (G) serves as the neighboring weed species. P and G seeds were harvested from a field at Agroscope, Changins, Switzerland. Single cartridges contained individual plants of black oat (BO) and weeds (X). Plants were grown under controlled conditions (16/8-hour light/dark cycle at 25/20 °C, 200 μmol m^-^² s^-^¹, and 70% RH) with 5 mL of half-strength Hoagland's solution added every other day. To control potential positional effects, trays of plants were moved to different locations within the phytotron at regular intervals. Three independent trials were conducted, each with five biological replicates per treatment. Due to plant mortality, the final number of replicates per trial ranged from three to five, resulting in 12–15 replicates per treatment across all trials. Root exudate collection was initiated on day 14 after split-root establishment. Day 14 was deemed suitable because shorter incubation periods were not feasible due to slow early development of the weed species, and extended growth periods led to nutrient deficiency symptoms in black oats. Root exudate collection followed by root system architecture measurements.

Root exudates were collected from all split and non-split root plants (**Figure 1a**) using an SPE vacuum manifold (Macherey-Nagel) with a Buchi V-300 vacuum pump and I-300 Pro interface set to 780 mbar to maintain 5 mmHg pressure in the glass chamber. Nanopure water (30 ml) was added over 30 seconds by hand using a pipette; valves were kept open for an additional 30 seconds (total of 1 minute per sample). The collection was done in a randomized order. After the aqueous extraction, or "first aqueous extract", which captured water-soluble metabolites from the undisturbed biological system, 15 mL of nanopure water was added, and plants were returned to the phytotron. For split-root plants, two adjacent valves were used simultaneously to collect the root exudate samples.

After a 24-hour exudate regeneration period, a second extraction was performed by adding 30 mL of extraction solution [95% methanol, 4.95% nanopure water, 0.05% formic acid with internal standard (3,5-di-tert-butyl-4-hydroxybenzoic acid, 0.5 µmol L^-^¹)]. This solution was applied over 30 seconds and vacuumed for a total of one minute to generate the "second methanolic extract". This captured a wider breadth of metabolites and reduced matrix variation, though at the cost of greater biological disturbance. This dual-extraction protocol captures readily available exudates after a two-week accumulation period (first extract) and following a 24-hour regeneration (second extract), reflecting discrete snapshots of exudate accumulation and turnover rather than time-series patterns. Each sample (15 mL from aqueous and methanolic extractions) was evaporated using a vacuum concentrator (Genevac EZ-2 Plus) at 35 °C for one hour in HPLC mode then aqueous modes until reduced to ~200 µL.

Following the root exudate extraction, root system architecture (RSA) of BO and X plants grown in single cartridges and neighbor plants NBO and NX grown in split-root B compartments (**Figure 1a**) were digitalized using WinRHIZO^TM^ as described in Eroğlu et al. (2024). After drying the tissues at 50 °C for 48 h, aboveground dry weight (mg) and root dry weight (mg) were recorded (**Table 1**). Neighbor plants (NP, NG, and NBO) grown in the B compartments of the split-root system shared the compartment with one half of the black oat root system, whereas plants grown in single cartridges (P, G, and BO) developed in isolation. Comparisons between neighbor plants in split-root systems and single-cartridge plants were therefore used to assess the influence of black oat root presence, while acknowledging that structural and handling differences between growth systems may influence root growth and exudation patterns. Due to these limitations, it was necessary to perform the root exudate application experiments described in the next section to discern changes in neighbor plants arising from the physical presence of another plant competing for space versus from the exudates coming from the cover crop.

**Table 1:** Effects of split-root black oat plants on the root traits of neighboring black oat (NBO), redroot pigweed (NP), and blackgrasss (NG) plants. Values represent adjusted marginal means ± SE (n = 12–15).

Root traits	BO	NBO	P	NP	G	NG
Root length (cm)	202 ± 15.8	90 ± 15.7***	117.4 ± 31.2	69.2 ± 31.2***	73.7 ± 6.77	118.0 ± 6.44***
Specific root length (cm mg^-1^)	7.54 ± 0.56	6.92 ± 0.56	36.4 ± 7.66	21.7 ± 7.67***	18.2 ± 1.09	22.0 ± 1.10*
Average root diameter (mm)	0.338 ± 0.025	0.316 ± 0.025	0.272 ± 0.006	0.271 ± 0.006	0.255 ± 0.024	0.244 ± 0.024
Root volume (cm^3^)	0.158 ± 0.016	0.088 ± 0.016**	0.07 ± 0.02	0.04 ± 0.02***	0.043 ± 0.008	0.052 ± 0.008
Number of root tips	628 ± 9.05	388 ± 52.7***	379 ± 106.0	226 ± 63.3***	229 ± 19.6	412 ± 32.7***
Root surface area (cm^2^)	18.96 ± 1.10	9.53 ± 1.03***	10.35 ± 2.76	6.19 ± 2.77***	6.48 ± 0.78	8.62 ± 0.79*
Aboveground dry weight (mg)	43.0 ± 3.44	26.6 ± 3.40***	7.68 ± 3.55	10.84 ± 3.56***	8.76 ± 0.912	9.17 ± 0.912
Root dry weight (mg)	28.0 ± 2.54	13.8 ± 2.51***	4.05 ± 1.18	3.34 ± 1.20	3.86 ± 0.35	5.07 ± 0.34**

Treatment effects between BO, P and G plants grown alone and those grown with BO in split-root systems (NBO, NP and NG) were evaluated using model−based tests on fixed effects. Significance was denoted as ns (P > 0.05), * (P < 0.05), ** (P < 0.01), or *** (P < 0.001).

All analyses were done with the statistical software R, version 4.5.2 “[Not] Part in a Rumble” (R Core Team, 2025). Seven continuous root and shoot traits were analyzed using linear mixed−effects models fitted with the ‘nlme**’** package (Pinheiro and Bates, 2000; Pinheiro et al., 2025). Split-root systems of i) black oat (BO) plants alone on root traits of neighboring black oat (NBO); ii) redroot pigweed (P) grown alone on root traits of neighboring redroot pigweed (NP), and iii) blackgrass (G) grown alone on root traits of neighboring blackgrass (NG) were compared as BO v NBO, P v NP and G v NG, respectively.

In the linear mixed-effects model, the treatments for the comparisons were considered as fixed effects, and repetition experiment was included as a random intercept to account for variation among experimental runs (3 experiments). Because residual variability differed among experiments, a heterogeneous variance structure was modelled using a ‘varIdent’ weighting function. The count variable (number of root tips) was analyzed using a negative binomial generalized linear mixed−effects model fitted with the package ‘glmmTMB**’.** Here, treatment was used as a fixed effect, experiment as a random intercept, and an experiment−specific dispersion structure (dispformula = ~ 0 + Exp) to accommodate heterogeneous dispersion across experiments. Adjusted marginal means (EMMs) and their standard errors (SE) for BO v NBO, P v NP and G v NG were obtained using the ‘emmeans**’** package (Lenth, 2023). Treatment effects were evaluated using model−based tests on fixed effects. Significance was denoted as ns (P > 0.05), * (P < 0.05), ** (P < 0.01), or *** (P < 0.001).

### 2.2 Black oat root exudate treatment on P and G

Root exudates BO-0, BO-X/A and BO-X/B (**Figure 1b**) were extracted using the same dual extraction method as explained in section 2.1 to prevent varying salt concentrations across different experimental conditions (verified by pH and electrical conductivity measurements). Exudate solutions showed comparable values, with pH approximately 4.0 and EC around 1.0 mS cm^-^¹. The decrease in pH relative to the half-strength Hoagland’s solution likely reflects root-mediated acidification and metabolic activity. For the second extraction performed after 24 hours, nanopure water was used instead of the extraction solution. Organic solvents would be directly toxic to the treated weeds which would obscure the effects of the exudates and compromise the accuracy of the results. Ten individual exudate samples per condition were combined to form a single homogeneous solution, which was applied to recipient seedlings. Thus, the pooled solution represented the experimental unit for all downstream treatments, and treatment effects were assessed at the level of plant responses (**Figure 1b**).

Ten P and five G seeds were sown into single cartridges, which received 3 mL of half strength Hoagland's solution daily and thinned to three seedlings per tube 5 DAS. For the next ten days, P and G received daily either 3 mL exudates of BO-0 for P(BO-0), BO-P/A for P(BO-P/A) or BO-P/B for P(BO-P/B) and similarly BO-0 for G(BO-0), BO-G/A for G(BO-G/A), or BO-G/B for G(BO-G/B) (**Figure 1b**). Controls received 3 mL of nanopure water daily. All plants, both control and root exudate treated, received 1 mL of half-strength Hoagland's every other day. Because the exudates were collected in a matrix containing residual Hoagland nutrients following washout, producing an equivalent salt-only matrix control was technically challenging. The residual nutrient content in exudate solutions could not be precisely replicated, and using Hoagland solution as the control would have resulted in control plants receiving higher total nutrient levels than root exudate treated plants.

15 DAS, RSA measurements were performed as described in the previous section. Neighbor species differed in developmental stage at the time of interaction due to inherent differences in germination and growth rates, with redroot pigweed at the fourth-leaf stage and blackgrass at the second-leaf stage. Statistical significance (p < 0.05) of root traits was assessed by conducting either a student's t-test or one-way ANOVA. As a *post hoc* test, Tukey’s multiple comparisons test was performed by comparing the mean of each condition with the mean of every other condition. Statistical analysis was performed using R. The experiment was performed with 10 replicates.

### 2.3 Sample preparation for LC-HRMS/MS analysis

Pre-concentrated sample extracts (~200 µL) were reconstituted to 10% methanol and 0.1% formic acid and to be a 30-fold concentration of the original volume (500 µL total). Aqueous extracts formed particulates during preconcentration and were ultracentrifuged at 125,000 g for 15 minutes at 4 °C before analysis.

Quality control (QC) were pooled samples from each experimental set (P and G) or extraction type (“first aqueous extract” and “second methanolic extract”) for a total of 4 different QC samples and data sets (P aqueous and methanolic, G aqueous and methanolic). Samples were separated into two aliquots for negative and positive ionization modes and stored at -80 °C until analysis.

### 2.4 Mock root exudate mixture

A mock root exudate mixture was made up of 58 chemical standards at 10 µmol L^-^¹ concentration to serve as reference for building an in-house database (**Supplementary Table S1**). They were known from literature to be exuded by agricultural plant roots. This mixture was used to assess the repeatability of signal intensity and retention times of the instrument on different days of liquid chromatography mass spectrometry (LCMS) analysis.

### 2.5 LC-HRMS/MS data acquisition

The liquid chromatography high-resolution tandem mass spectrometry (LC-HRMS/MS) system consisted of a cooled autosampler unit, two binary pumps, and a temperature-controlled column compartment (1290 Infinity II) brought the sample to the primary sprayer of a 6560-ion mobility QTOFMS with a dual Agilent jet stream electrospray ionization interface. A nano pump for reference mass solution (1260 Infinity) led to the secondary sprayer.

Samples were thawed, vortexed, and stored at 4 °C in the autosampler until injection. All samples were randomized for injection order. A QC sample was run every 14–16 injections.

A single-column method was implemented for the “first aqueous extract” samples. The stationary phase was comprised of a Discovery HS F5 (150 x 2.1 mm, 3 μm particle size, Sigma-Aldrich) pentafluorophenyl (PFP) column paired with a Discovery HS F5 Supelguard Cartridge. Gradient details can be seen in **Supplementary Table S2a** and mobile phase details are in **Supplementary Table S2d**.

For the “second methanolic extract”, a dual-column method developed by (Bennett et al., 2024) was utilized. The Discovery HS F5 PFP column and a Hypercarb porous graphitic carbon (PGC) column (2.1 × 150 mm, 5 μm particle size, Thermo Scientific) equipped with a Hypercarb PGC guard column were connected to a valve within the column compartment which controlled the flow from the two separate binary pump systems. Gradient details are in **Supplementary Tables S2b, c** and mobile phase details are in **Supplementary Table S2d**.

The MS method was the same for single and dual column methods. Data was acquired using data-dependent acquisition (DDA) with negative and positive ionization modes performed as separate runs. A detailed report of the HR-MS/MS acquisition method is given in **Supplementary Table S2d**.

### 2.6 LC-HRMS/MS data analysis

#### 2.6.1 Data pre-processing

Data was recalibrated and centroided using Agilent reprocessing software. Initial pre-processing was performed in MS-DIAL (v4.9.221218) with a void volume cutoff of 1.6 min (single-column method) or 2.0 min (dual-column). Isotopologues, but not their adducts, were aggregated, and a 20% blank filter was applied using an extract with glass beads and no plant. Data was normalized to the internal standard for the “second methanolic extract”. For both extracts, further normalization was performed using a pooled QC sample adjusted via LOWESS regression to account for signal drift and batch effects. Gap filling addressed missing or zero values. Pre-processing details are in **Supplementary Table S3**. Further analysis in R aggregated adducts with protonated/deprotonated features to generate a “total compound signal” (TCS), applied an S/N cutoff of 10, and normalized samples to root weight.

#### 2.6.2 Statistical analysis

Statistical analysis was conducted in R on TCS values. Outliers were removed using the 1.5x interquartile range rule. Among-group variation was assessed with F-tests or Bartlett’s tests, and normality with Shapiro-Wilk’s test. Significant differences between experimental conditions (e.g., P or G experimental setups: A compartment split-root conditions BO-0/A, BO-BO/A and BO-P/A or BO-G/A; single-cartridge conditions BO and P or G) were determined using Welch’s t-test with false discovery rate (FDR) correction. Fold changes were calculated pairwise. Data was centered, auto-scaled, and analyzed via principal component analysis (PCA) and partial least squares-discriminant analysis (PLS-DA) for each experimental set. Due to QC normalization requirements and equipment limitations, P and G experimental sets were acquired and analyzed separately for “first aqueous extracts” and “second methanolic extracts” and all relative quantification comparisons are based on these four datasets. Consequently, cross-species comparisons (P vs. G experimental sets) are qualitative in nature, limited to patterns, compound classes, and counts of differentially accumulated compounds, rather than direct comparisons of absolute signal intensities. To assess whether pooling of compound class distribution data was appropriate for BO-0/A and BO-BO/A conditions shared across P and G datasets, Pearson's chi-squared tests were performed on compound counts for these conditions. Specifically, pairwise Welch’s t-tests were performed with unequal variances, and FDR correction was applied per metabolite feature using the Benjamini–Hochberg method. Metabolites were prioritized for biological interpretation using a dual threshold of FDR < 0.05 and |log_2_ fold change| > 0.6. PCA was performed with centering and scaling, and PLS-DA was conducted for supervised classification. While this approach does not account for the non-independence of compounds within samples, it provides a practical assessment of distributional similarity for visualization purposes.

To assess potential confounding by biological batch, batch effects were first evaluated by permutational multivariate analysis of variance (PERMANOVA) on feature matrices and found evidence of batch effects for some conditions. To address this, a sensitivity analysis then compared the primary (uncorrected) results against a linear mixed-effects model with treatment as a fixed effect and batch as a random intercept. Concordance in metabolite prioritization (FDR < 0.05 and |log_2_FC| > 0.6) ranged from 96.2–97.1% across all datasets (**Supplementary Figures S1**, **S2**). To avoid over-correction of genuine biological variation of metabolomics data (Nygaard et al., 2016; Goh et al., 2017), results from the primary analysis are presented.

#### 2.6.3 Identification

Identification followed levels 1–4 as described by Schymanski et al., 2014. It is important to note that 'identification' at different confidence levels represents varying degrees of structural certainty: levels 1–2 provide definitive or probable structural identification, while levels 3–4 represent compound class assignments or molecular formula determinations, respectively, without definitive structural elucidation. For level 1, an in-house database was created using MS-FINDER from 58 mock root exudate standards and applied to sample sets in MS-DIAL. Level 2 was divided into 2a (spectral database match) and 2b (in silico database match). Level 2a utilized GNPS and MS-FINDER databases, favoring the best-scored result. MS-FINDER results were preferred when both databases returned matches. Since each database frequently used different synonyms for the same compound, it was not viable to assess if the databases returned the same or different results for each compound. For level 2b, structure scores >6 (MS-FINDER) or >0.6 (SIRIUS) were required, with the highest score of all formulas selected and MS-FINDER preferred for overlaps. MS-FINDER was selected as the preferred software for level 2 identification as it performed better when benchmarked against the mock root exudate mixture. CANOPUS (SIRIUS) determined compound classes (level 3) using fragmentation patterns. Thresholds of >0.80 (class), >0.95 (superclass), and >0.90 (subclass) were applied. Molecular formulas (level 4) were assigned using SIRIUS and MS-FINDER based on parent m/z and adduct/ionization form information. Both formulas were kept and used for validation / corroboration during manual identification of prioritized metabolites, but as there were many high scoring and conflicting candidates, formulas were not reported.

Black oat specialized metabolites were absent from the major databases and lacked analytical standards, as both resources favor primary and well-studied metabolites. Therefore, avenaol (Kim et al., 2014) and scopoletin (Sánchez-Moreiras et al., 2003) required manual searches of MoNA and PubChem databases and hand annotation using diagnostic fragment ions (**Supplementary Table S4**). Without available authentic standards, avenaol, scopoletin, and scopoline (a scopoletin glycoside) were classified as confidence level 2a identifications based on fragment ion matches to database spectral records.

#### 2.6.4 Prioritization & visualization

Metabolites with an FDR-corrected p-value <0.05 and a |log_2_ fold change| >0.6 were prioritized and considered to be “more accumulated.” Differentially accumulated compounds (DAC) were defined as “more accumulated” in BO exudates by the presence of any neighbor in comparison BO-0/A. To visualize this prioritization, volcano plots were generated to display p-values versus log_2_-fold changes for comparisons between experimental groups. PC1 and PC2 of PCA and PLS-DA were plotted to distinguish three or more sample groups. Feature-based molecular networks were generated in GNPS for the “second methanolic extracts” (positive/negative ionization for P and G experimental sets) to compare fragmentation patterns. Related compounds were given a cosine score with a threshold of 0.6 to quantify how similar they were. All compounds were plotted in relation to one another. As node proximity to one another is not a perfect visualization of compound relatedness with this method, the line thickness of the edge between nodes was based on the cosine relationship score. Identification information was integrated; nodes were colored by compound class (identification level 3). Identification level 1 and 2a compounds were annotated by name. Confidence level 1 and 2a labels for identified compounds were placed above the nodes they represent. Prioritization information was integrated by highlighting compounds of interest in bold black outlines around the representative node. Compounds more accumulated in BO-P/A when compared to BO-0/A were noted by diamond nodes, and those more accumulated in BO-G/A when compared to BO-0/A were noted by triangular nodes.

Prioritized compounds underwent additional evaluation beyond automated identification. Outputs from identity confirmation processes were compared for consistency with fragmentation patterns and experimental data. While the identification thresholds of the different software were not ignored, if multiple identification results passed the given thresholds, a lower-ranked annotation was selected over a higher-ranked one if the connections between compounds in the network corroborated with the lower-priority result.

## 3 Results

### 3.1 Effect of black oat on neighboring redroot pigweed and blackgrass

Black oats grown alone in single cartridges (BO) were compared with neighboring black oat (NBO) grown alongside one half of a split-root black oat system in a shared compartment (BO-BO/B; **Figure 1a**). Neighboring black oat showed significant reductions in root length (-55%), root volume (-44%), number of root tips (-38%), root surface area (-50%), aboveground dry weight (-38%), and root dry weight (-51%) compared to BO grown alone, whereas specific root length and average root diameter were not significantly affected.

Redroot pigweed grown with split-root black oat (NP) exhibited significantly lower root length (-41%), specific root length (-40%), root volume (-43%), number of root tips (-40%), and root surface area (-40%) compared to redroot pigweed grown alone in single cartridges (P). In contrast, aboveground biomass of NP was significantly higher than P (+41%). Average root diameter and root dry weight were not significantly different between NP and P.

Blackgrass grown with split-root black oat (NG) showed significant increases in root length (+60%), specific root length (+21%), number of root tips (+80%), root surface area (+33%), and root dry weight (+31%) compared to blackgrass grown alone in single cartridges (G), while average root diameter, root volume and aboveground biomass remained unchanged.

These results indicate that interaction with split-root black oat significantly affected neighboring plant root traits in a species-specific manner, reducing growth in redroot pigweed and black oat, while enhancing several root traits in blackgrass.

### 3.2 Root exudate treatment on redroot pigweed and blackgrass

Root exudates collected from black oat split-root systems had pronounced effects on redroot pigweed (P) root traits, whereas effects on blackgrass (G) were less pronounced (**Table 2**; **Figure 1b**).

**Table 2:** Effect of root exudates collected from black oat split-root systems on redroot pigweed (P) and blackgrass (G) root traits.

Redroot pigweed traits	P (BO-0)	P (BO-P/A)	P (BO-P/B)	P (water)
Root length (cm)	60.8 ± 7.1^ns^	55.9 ± 7.6^ns^	36.9 ± 7.1^ns^	47.8 ± 7.6^ns^
Average root diameter (mm)	0.31 ± 0.01^a^	0.26 ± 0.02^ab^	0.3 ± 0.01^ab^	0.25 ± 0.02^b^
Root volume (cm^3^)	0.04 ± 0.00^a^	0.03 ± 0.00^ab^	0.02 ± 0.00^ab^	0.02 ± 0.01^b^
Number of root tips	122 ± 13.1^b^	153.4 ± 14.0^ab^	106.9 ± 13.1^b^	197.5 ± 15.1^a^
Root surface area (cm^2^)	5.5 ± 0.5^a^	4.0 ± 0.5^ab^	3.4 ± 0.5^b^	3.1 ± 0.5^b^
Aboveground dry weight (mg)	12.9 ± 0.7^a^	11.7 ± 0.7^a^	10.6 ± 0.7^a^	7.7 ± 0.75^b^
Root dry weight (mg)	3.01 ± 0.23^a^	3.01 ± 0.24^a^	1.84 ± 0.23^b^	1.93 ± 0.24^b^
Blackgrass traits	G (BO-0)	G (BO-G/A)	G (BO-G/B)	G (water)
Root length (cm)	123.0 ± 15.6^ns^	104.4 ± 15.6^ns^	115 ± 12.3^ns^	112.3 ± 14.2^ns^
Average root diameter (mm)	0.27 ± 0.01^ns^	0.25 ± 0.01^ns^	0.23 ± 0.01^ns^	0.25 ± 0.01^ns^
Root volume (cm^3^)	0.07 ± 0.01^ns^	0.05 ± 0.01^ns^	0.05 ± 0.01^ns^	0.06 ± 0.01^ns^
Number of root tips	337.0 ± 27.7^ns^	274.0 ± 27.7^ns^	302.6 ± 23.42^ns^	287.7 ± 25.3^ns^
Root surface area (cm^2^)	10.1 ± 1.18^ns^	8.1 ± 1.18^ns^	8.4 ± 0.93^ns^	9.0 ± 1.08^ns^
Aboveground dry weight (mg)	9.52 ± 0.51^a^	7.44 ± 0.51^b^	7.91 ± 0.43^ab^	7.26 ± 0.51^b^
Root dry weight (mg)	6.72 ± 0.52^ns^	4.90 ± 0.52^ns^	5.45 ± 0.41^ns^	5.23 ± 0.48^ns^

The first table shows the mean value ± SEM of redroot pigweed traits, and the second table shows the mean value ± SEM of blackgrass traits (n = 5-8, each data point is an average of 3 P and G seedlings). Redroot pigweed and blackgrass seedlings received root exudates obtained from the A compartments of black oat split-root systems without neighbors P (BO-0) and G (BO-0). P seedlings received root exudates obtained from black oat split-root systems with P neighbors: having no direct root contact P(BO-P/A), and with direct root contact P (BO-P/B), while G seedlings received root exudates obtained from split-root systems with G neighbors: having no direct root contact G (BO-G/A), and direct root contact G (BO-G/B), and all controls received nanopure water. One-way ANOVA with Tukey’s multiple comparisons test was performed. Different letters (a, b, ab) indicate statistically significant differences between groups (p < 0.05), while “ns” denotes no significant difference.

In redroot pigweed, no significant differences in root length were observed between the treatments, although there was a tendency for shorter roots in the BO-P/B treatment and the water control compared to the other treatments. Similar trends were observed for root diameter and root volume, which were significantly lower by 19% and 50%, respectively, in the water control compared to BO-0. Root surface area was reduced by 38% in BO-P/B and by 44% in the water control compared to BO-0. The aboveground biomass of redroot pigweed was reduced between 27% and 40% in the water control relative to all exudate treatments. Root dry weight was 39% lower in BO-P/B and 36% lower in the water control compared to BO-0 and BO-P/A. Conversely, the water control had the highest number of root tips compared to BO-0 (+62%) and BO-P/B (+85%). In blackgrass, root exudates had minimal effects on root architecture, with root length, diameter, volume, number of root tips, root surface area, and root dry weight showing no significant differences across treatments. The only significant effect was observed in aboveground biomass, which was higher in G(BO-0) compared to G(BO-G/A) and the water control, while G(BO-G/B) was between these two values.

These results indicate that black oat exudates differentially affected the two neighbor species, producing stronger responses in redroot pigweed than in blackgrass.

### 3.3 Statistical analysis of LC-HRMS/MS root exudate data shows modest but statistically robust neighbor-induced metabolic changes

**Figure 2:**
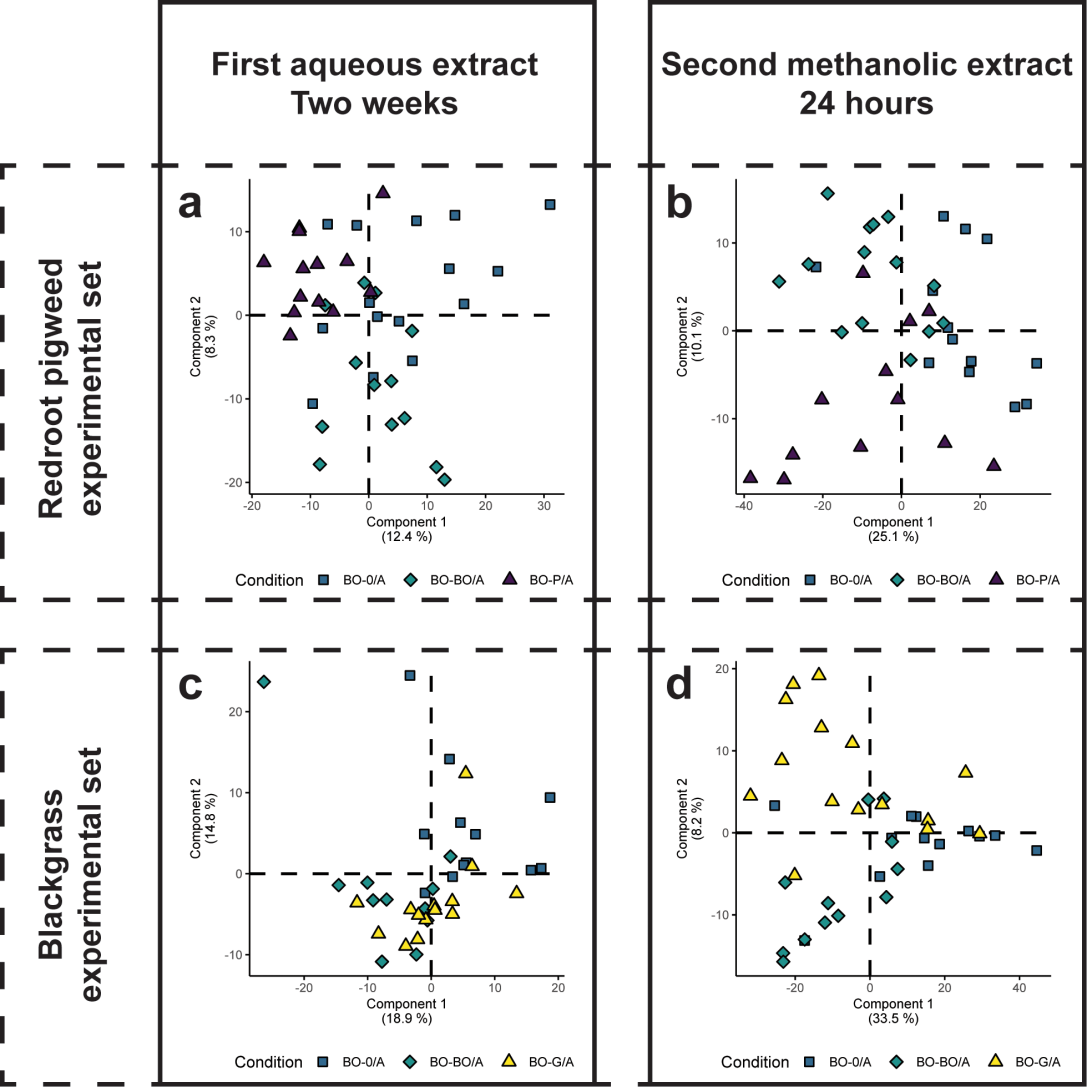
Partial least squares discriminant analysis (PLS-DA) of black oat root exudate metabolomes across neighbor treatments and extraction methods. Panels **(a, b)** show PLS-DA for the redroot pigweed experimental set in the first aqueous (two weeks) and second methanolic (24 hours) extracts, respectively. They highlight differences between compartment A with no neighbor (BO-0/A), with a same species neighbor (BO-BO/A), and with a redroot pigweed neighbor (BO-P/A) groups. Panels **(c, d)** show PLS-DA for the blackgrass experimental set in the first aqueous and “second methanolic extracts”, respectively, comparing the same neighbor conditions (BO-0/A, BO-BO/A) with a blackgrass neighbor (BO-G/A).

Within all the PCAs (**Supplementary Figure S3**), the 10 of the 11 QC samples analyzed for each experimental setup (P aqueous, P methanolic, G aqueous, and G methanolic) clustered together (with the first QC sometimes not clustering well). Samples containing P or G root exudates separated from samples containing only BO root exudates. There was a high degree of overlap for all the extracts from BO plants, regardless of the interaction / tested condition. Similarly, the PLS-DA (**Figure 2**) also showed a high degree of overlap of BO extracts for the “first aqueous extract”. For the “second methanolic extract” there was a better separation in the PLS-DA, but there was still some overlap between groups within the center of the PLS-DA for both P and G experimental sets.

**Figure 3:**
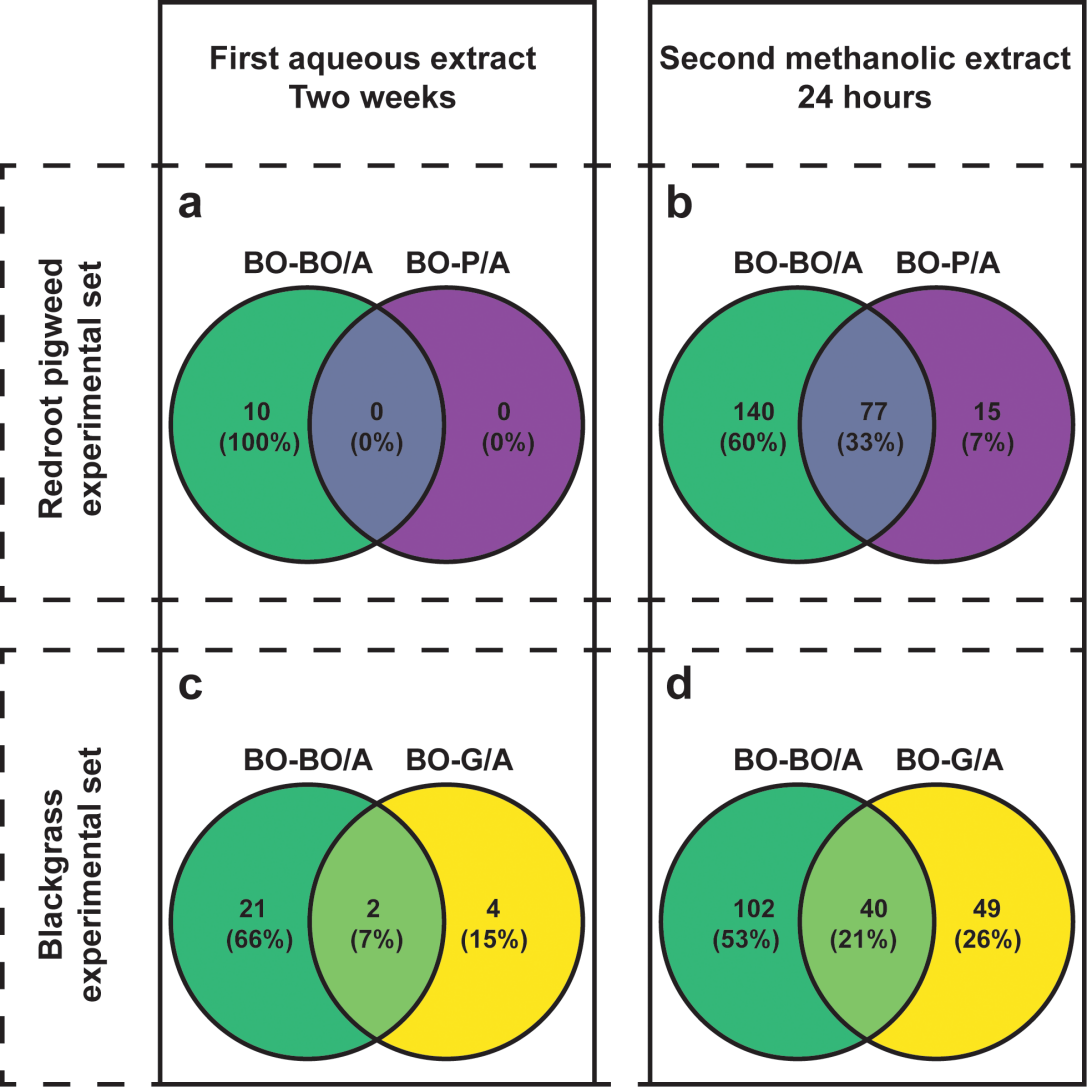
Venn diagrams of differentially accumulated compounds in black oat exudates across neighbor types and extraction methods. Panels **(a, b)** display unique and shared differentially accumulated compounds between black oat with a black oat neighbor (BO-BO/A, green) and with a redroot pigweed neighbor (BO-P/A, purple) for the first aqueous and “second methanolic extracts,” respectively. Panels **(c, d)** show the same comparison for black oat neighbor (BO-BO/A, green) and blackgrass neighbor (BO-G/A, yellow). Compounds were considered differentially accumulated when significantly higher in neighbor treatments compared to black oat alone (BO-0/A), as determined by Welch's t-test (FDR-corrected p < 0.05) with |log_2_ fold change| > 0.6.

Univariate analysis identified group-specific metabolites across both extracts. In the “first aqueous extract”, redroot pigweed and blackgrass each accumulated hundreds of compounds compared to black oat when grown alone (335 and 157 compounds, respectively). However, black oat showed strikingly different responses to neighbor presence: no metabolites were DAC in black oat when paired with redroot pigweed (BO-P/A), while only 6 compounds changed in response to blackgrass neighbors (BO-G/A) (**Figure 3a**, 3c).

The “second methanolic extract” revealed substantially more neighbor-induced changes in black oat (**Figures 3b, d**). When grown alone, redroot pigweed and blackgrass again showed 207 and 303 more abundant compounds than black oat, respectively (**Supplementary Figures S4**, **S5**; **Supplementary Tables S5**, **S6**). Critically, black oat now exhibited clear metabolic responses to both neighbors: 92 compounds were differentially accumulated with redroot pigweed (BO-P/A; **Supplementary Table S7**; **Supplementary Figure S8**) and 89 with blackgrass (BO-G/A; **Supplementary Table S8**; **Supplementary Figure S9**). Although separate acquisition and normalization preclude direct quantitative comparison of specific metabolites between experimental sets, these similar counts suggest comparable response magnitudes, and the shared BO-0/A baseline enables valid cross-set pattern comparisons. These patterns align with the PLS-DA (**Figure 2**), confirming greater observable differences in the “second methanolic extract.

The limited group separation in PCA and PLS-DA occurs because neighbor-induced changes target specific metabolites rather than reorganizing overall composition. While numerous compounds met statistical thresholds as DAC (FDR p < 0.05, |log_2_ fold change| > 0.6), most showed modest fold changes that do not dominate overall variance. Additionally, PCA and PLS-DA display individual samples, which show considerable overlap between groups, whereas DAC analysis compares group means, where consistent directional changes achieve statistical significance despite individual sample variability.

### 3.4 Structural characterization reveals compound class shifts in response to neighbors

**Figure 4:**
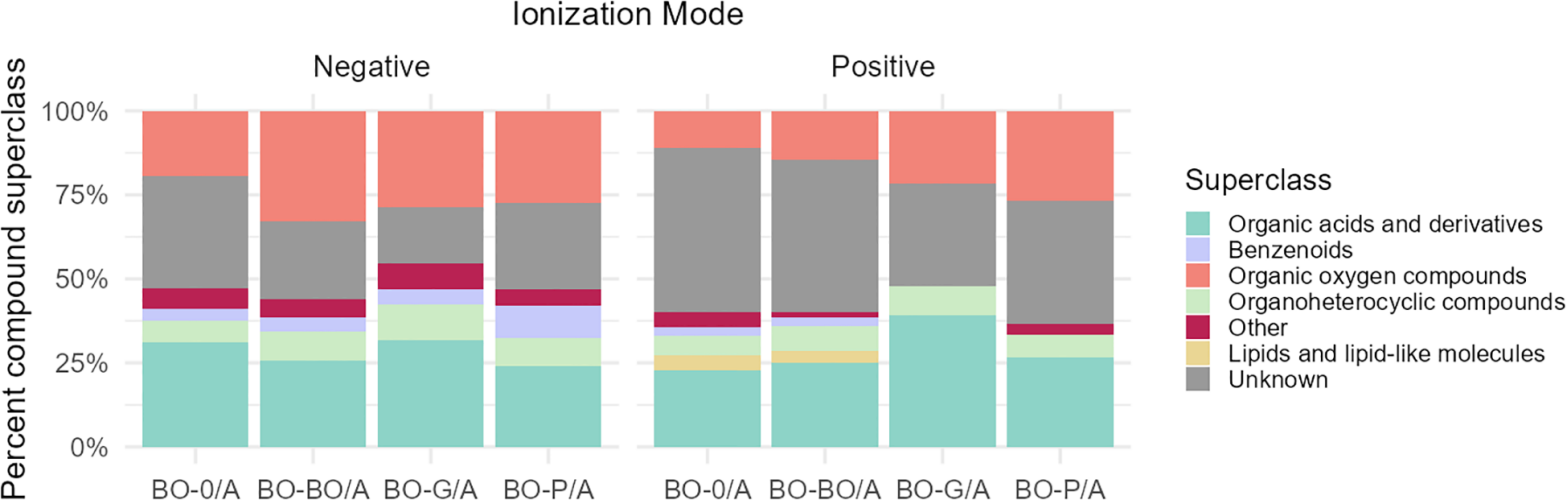
Superclass distribution of differentially accumulated compounds in black oat root exudates from the “second methanolic extract.” Compound classes of exudates were annotated using the CANOPUS application of SIRIUS. Compounds shown for black oat alone (BO-0/A) were those not differentially accumulated in redroot pigweed or blackgrass (i.e., not significantly different between black oat and either weed species), as determined by Welch's t-test (FDR-corrected p < 0.05) and |log2 fold change| > 0.6. Compounds that were more accumulated by the presence of an intraspecific neighbor (BO-BO/A), an interspecific redroot pigweed neighbor (BO-P/A), and an interspecific blackgrass neighbor (BO-G/A) were those which were significantly higher than BO-0/A using these same thresholds. Since BO-0/A and BO-BO/A conditions were present in both the pigweed and blackgrass experimental comparisons, compounds from both datasets were pooled for these categories in the stacked bar graphs. Compound classes which comprised less than 5% of the total number of compounds for that ionization mode were binned together into the “other” category.

Depending on the data set, ~1-2% of compounds were annotated to confidence level 1 and ~2-4% and 4-7% were identified to confidence levels 2a and 2b, respectively (**Supplementary Tables S9**–**S11**; **Supplementary Figure S10**. Notably, no confidence level 1 compounds were unambiguously identified as DAC (**Supplementary Tables S5**–**S8** highlight confidence level 1 and 2 identification). Many detected features (45-65%) were assigned to confidence level 3, representing compound class annotations based on fragmentation patterns rather than definitive structural identifications. Superclass annotation of different experimental groups from the “second methanolic extracts” show that the DAC are comprised of more organic oxygen compounds (**Figure 4**). For BO-0/A and BO-BO/A conditions shared across P and G datasets, chi-squared tests (p = 0.37 and p = 0.64) and visual inspection (**Supplementary Figure S12**) confirmed similar class distributions, supporting cross-set pattern comparisons. However, these comparisons remain qualitative, reflecting class distribution patterns rather than absolute intensities. Probabilistic subclass annotation from CANOPUS indicates that amino acids and carbohydrates are present in both the "first aqueous extracts" and "second methanolic extracts" (**Supplementary Figure S13**). Known allelochemicals were searched for in the extracts. Avenaol was not detected, possibly due to method incompatibility or its absence in the samples. Scopoletin and scopolin were present in black oat root exudates but showed no increased accumulation across the A compartments (BO-0/A, BO-BO/A, BO-G/A).

### 3.5 Molecular networking identifies four structural clusters containing neighbor-responsive metabolites

**Figure 5:**
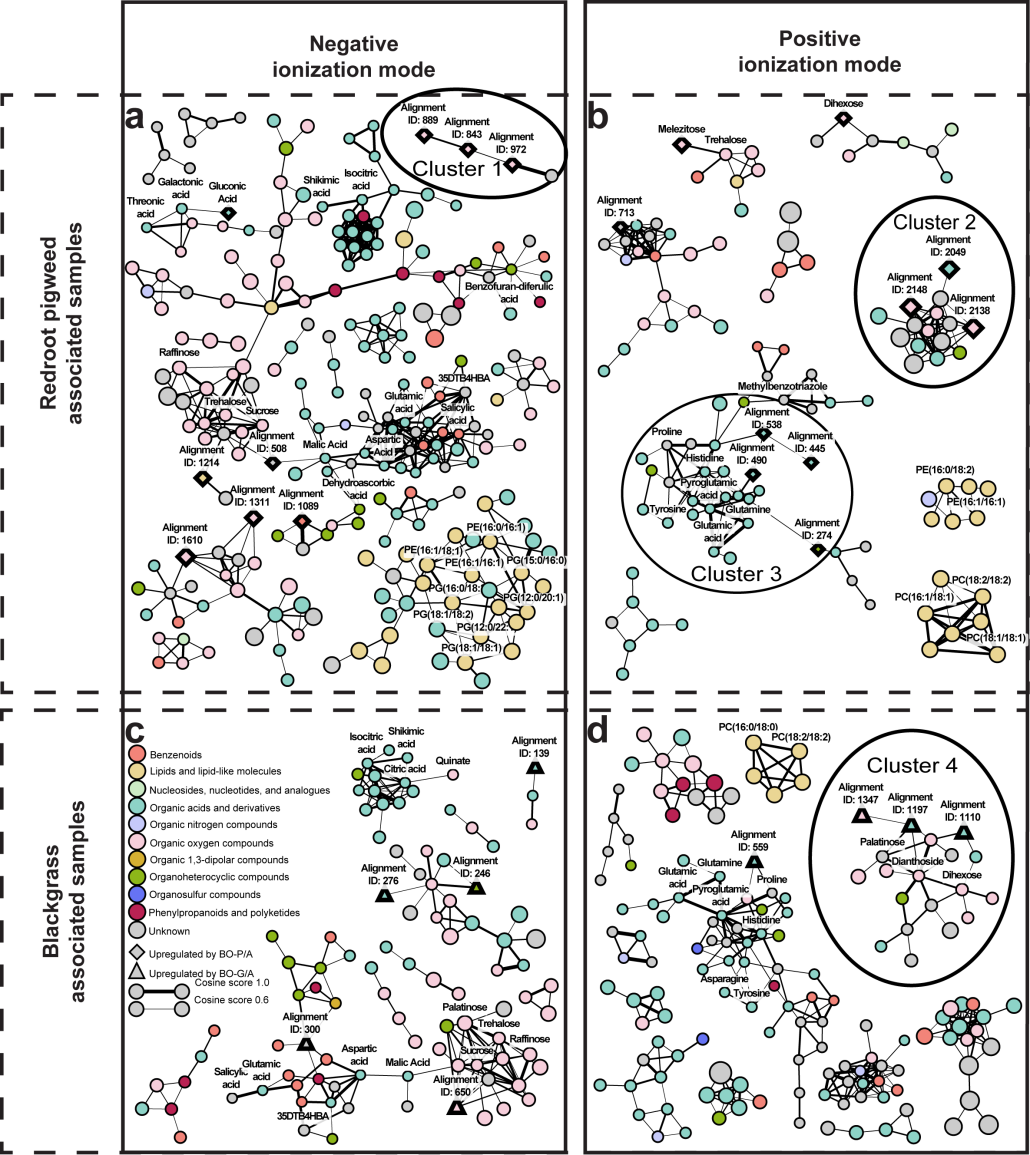
Molecular network analysis of black oat root exudates based on MS/MS spectral similarity. Networks were generated using GNPS (Global Natural Products Social Molecular Networking) to cluster structurally related compounds. Panel **(a)** shows the redroot pigweed experimental set in negative ionization mode, panel **(b)** shows the redroot pigweed experimental set in positive ionization mode, panel **(c)** shows the blackgrass experimental set in negative ionization mode, and panel **(d)** shows the blackgrass experimental set in positive ionization mode. Node color represents compound class as determined by the CANOPUS application of the SIRIUS open-source software. Node size represents the mass-to-charge ratio (m/z) of the analyte, with larger nodes representing larger m/z values. Edge width directly relates to the cosine relationship score indicating the strength of the fragmentation similarity, with thicker edges indicating higher scores. Diamond-shaped nodes represent compounds that are more accumulated by the presence of a redroot pigweed neighbor (BO-P/A) when compared to no neighbor (BO-0/A) as determined by Welch's t-test (FDR-corrected p < 0.05) and |log_2_ fold change| > 0.6. Triangle nodes denote compounds more accumulated by the presence of a blackgrass neighbor (BO-G/A). Four clusters, each containing three or more differentially accumulated compounds, are encircled for detailed structural discussion. Due to space constraints, phosphatidylethanolamine and phosphatidylglycerol abbreviations (PE and PG) were retained within the network.

Molecular networking of the "second methanolic extracts" from both P and G experimental sets (**Figure 5**) corroborated the compound identification and classification results; compounds with similar structures and fragmentation patterns clustered together according to their assigned superclass, providing orthogonal validation of the annotations. For instance, in the negative ionization mode network from the P experimental set, three confidence level 1 identified sugars (organic oxygen compounds superclass) formed a tightly connected cluster. Similarly, annotated phosphatidylethanolamines and phosphatidylglycerols (lipid and lipid-like superclass) clustered together alongside unknown organic acids and derivatives, while amino acids (organic acids and derivatives superclass) formed distinct clusters. This clustering pattern confirmed that compounds identified to confidence levels 1 and 2a aligned with their predicted superclass based on both database identification and spectral similarity relationships.

Four molecular network clusters containing three or more DAC were selected for detailed structural analysis (**Figure 5**; **Supplementary Figures S14**–**S17**). Three clusters contained compounds more accumulated in black oat in the presence of redroot pigweed neighbors, while one cluster contained compounds more abundant in redroot pigweed than black oat.

Cluster 1 consisted of DAC sharing a hexose sulfate functional group, evidenced by characteristic sulphate fragments (HSO_4_^-^, m/z 97) and hexose-sulphate adducts, along with consistent neutral loss of SO_3_ across all fragmentation patterns (**Figure 5a**; **Supplementary Figure S14**).

Cluster 2 contained DAC identified as aromatic amino acid derivatives based on shared fragments indicating a benzene ring, amino group, and carboxyl group (**Figure 5b**; **Supplementary Figure S15**). One non-DAC compound within this cluster (Alignment ID 1310) was structurally characterized as containing these aromatic amino acid features plus a dihexose moiety, though whether the DAC also contain dihexose remains unclear.

Cluster 3 DAC were annotated as amino acids by CANOPUS, classified under the organic acids and derivatives superclass, and shared characteristic carboxylic acid neutral losses in their fragmentation patterns, though definitively shared structural fragments were limited (**Figure 5c**; **Supplementary Figure S16**).

Cluster 4, containing compounds more accumulated in black oat in the presence of blackgrass neighbors, included three DAC with minimal shared fragmentation information and limited SIRIUS annotation, precluding much structural characterization (**Figure 5d**; **Supplementary Figure S17**).

## 4 Discussion

### 4.1 Differential root trait responses of redroot pigweed and blackgrass to black oat neighbor presence: outcomes from heterospecific interactions and root exudate applications

Root systems respond to neighboring plants through multiple mechanisms, including chemical signaling, competition and stress responses, even under non-limiting nutrient conditions (Yamauchi et al., 1996; Wang et al., 2006; Craine and Dybzinski, 2013; Belter and Cahill, 2015; Jacob et al., 2017; Lyu and Smith, 2022; Wu et al., 2023). In this study, we show that these responses are strongly species- and context-dependent. Redroot pigweed and blackgrass exhibited contrasting root responses to black oat, and their responses differed markedly between exposure to black oat root exudates and conditions allowing direct root–root interaction (**Figure 1**). These results highlight the complexity of root response regulation and help explain why predicting plant responses based on commonly measured root traits remains challenging, despite their widespread use in trait-based approaches (Freschet et al., 2021).

#### 4.1.1 Redroot pigweed

Treatment with root exudates from black oat grown alone (BO-0) significantly increased redroot pigweed aboveground biomass while other root traits such as root diameter, root volume, root surface area, and root dry weight showed non-significant increases. This response may reflect the presence of growth-promoting compounds in the black oat exudates, potentially sugars and amino acids (Hu et al., 2018). Together, these effects relative to the water control indicate a baseline growth-promoting influence of black oat exudates in the absence of neighbors.

However, the number of redroot pigweed root tips was significantly reduced by black oat root exudates from plants without a neighbor and from split-root black oat plants interacting with the weed, suggesting that redroot pigweed root tip number is influenced by black oat root exudates. Root exudates from split black oat roots in direct contact with redroot pigweed, compared to exudates from black oat grown alone significantly reduced root surface area, root dry weight and number of root tips, while root length showed a non-significant decreasing trend.

Under our experimental conditions, these exudates likely contained compounds released by both species, indicating a chemically mediated response to direct competition. This was reflected in the suppression of several redroot pigweed root traits and a concurrent increase in aboveground biomass, mirroring changes observed during direct black oat–redroot pigweed interactions (**Table 1**). In contrast, redroot pigweed exposed to exudates from black oat grown in adjacent, non-contact compartments did not show significant root trait suppression, suggesting that the absence of direct physical interaction may result in weaker or less inhibitory chemical signaling. These findings support previous work showing that root exudate composition and concentration can shift from growth-promoting to inhibitory (Gniazdowska and Bogatek, 2005; Dayan et al., 2009; Macías et al., 2020; Upadhyay et al., 2022). A similar inhibitory effect on redroot pigweed root traits was also reported in a previous study using buckwheat as a neighbor (Eroğlu et al., 2024), although in that case, both local and distal buckwheat root exudates similarly suppressed redroot pigweed root growth.

However, the increase in aboveground redroot pigweed biomass in all tested conditions suggests that root exudates of black oat may have triggered a strategic reallocation of resources from roots to shoots in redroot pigweed. While chemical cues from black oat root exudates may have played a role in these responses, species-specific growth tendencies and competitive strategies may have also influenced the biomass allocation. Previous field trials revealed that redroot pigweed growth is strongly suppressed in the presence of black oat, regardless of black oat biomass or light availability (Gfeller et al., 2018b). This underlines the hypothesis that below ground root interactions between redroot pigweed and black oat are responsible for reduced redroot pigweed development, both *in situ* and *ex situ*. This effect could be exploited in the field by using competitive or allelopathic black oat varieties to suppress the growth of redroot pigweed or other problematic weeds, for example by disrupting root development and altering biomass allocation. Given the problematic nature of redroot pigweed, its sensitivity to black oat supports the strategic use of black oat as a summer cover crop for sustainable weed management. Understanding the role of root-exuded compounds from black oat that affect redroot pigweed growth could further guide the development of improved black oat varieties for weed control.

#### 4.1.2 Blackgrass

In contrast to redroot pigweed, blackgrass root growth was not suppressed by the presence of black oat. No differences in root parameters were observed when blackgrass seedlings were treated with black oat exudates or water. However, a black oat neighbor stimulated blackgrass growth, with significant effects on most root traits. This distinct response suggests that blackgrass may engage in different interaction mechanisms or exhibit reduced sensitivity to black oat-derived cues. However, species-specific growth patterns and competitive strategy differences may also have contributed to the observed differences. Effects on aboveground biomass were less pronounced, and a significant increase was only observed when blackgrass was treated with exudates from black oat grown without neighbors, compared to other conditions. These results indicate that blackgrass is largely insensitive to exudates from black oat and that root traits remain stable under neighbor presence.

This response may reflect traits inherent to blackgrass. It is a noxious weed primarily affecting winter cereals, known for maintaining growth under stress, complete its vegetative cycle, and sustain the soil seedbank (Moss, 2017; Bitarafan and Andreasen, 2020; Harrison et al., 2024; Clark et al., 2025; Hickman et al., 2025). However, given the weak and limited effects observed in our experiments, our ability to make strong conclusions about blackgrass responses to black oat exudates is limited.

The differences in responses to black oat root exudates between redroot pigweed and blackgrass may reflect their biological traits in addition to any inhibitory or stimulatory effects of root exudates. While redroot pigweed is a summer annual dicot with a taproot system, blackgrass is a winter annual monocot with a fibrous root system. The differences in their root structure, growth timing, and resource use strategies could also help explain their differing responses to black oat exudates.

### 4.2 Neighbor identity shapes root exudate metabolome of black oat

Root exudate analysis revealed that the presence of redroot pigweed and blackgrass neighbors altered the metabolome profile of split-root black oat, even without direct root contact, compared to black oat without a neighbor. Studies suggest plants modify root exudates in response to environmental factors, including neighboring plants, triggering different local and systemic responses based on neighbor identity (Semchenko et al., 2014). Our previous research showed that buckwheat root exudate composition varied depending on whether its neighbor was buckwheat or redroot pigweed (Eroğlu et al., 2024). It should be noted that some of these compounds may be transformation products from the microbial community as these experiments were not performed under sterile conditions and microbiome metabolism can modify root exudate compositions.

#### 4.2.1 Dual extraction approach: complementary insights and differential detection

This study employed two sequential extracts to capture complementary information. The "first aqueous extract" captured undisturbed exudate accumulation over two weeks, closely mirroring natural conditions. The "second methanolic extract" benefited from prior removal of Hoagland's solution, minimizing matrix effects and ion suppression from nutrient salts. This extract also provided a 24-hour snapshot of exudate release and enabled better extraction of phenolics (Alara et al., 2021) and lipid-like molecules. Keeping the extracts separate preserved these distinct temporal and chemical profiles, which would have been lost if combined. Methanol was restricted to the second extraction to avoid plant stress and ensure sequential sampling feasibility, with brief (one-minute) application minimizing cell lysis risk (Pétriacq et al., 2017).

The "second methanolic extracts" showed better group separation in PLS-DA than the "first aqueous extracts" (**Figure 2**), with more exudate variation explained by experimental conditions. This difference likely stems from Hoagland's salts in the "first aqueous extract" causing ion suppression and salt adduct formation, reducing sensitivity and increasing within-group variation (Leitner et al., 2007; Furey et al., 2013). Additionally, the two-week collection period of the first extract may have allowed exudate composition to stabilize as plants reached homeostasis (McLaughlin et al., 2023), whereas the 24-hour window of the second extract captured more dynamic between-group variation. Removing exudates for the first extract may have reset this equilibrium, allowing clearer differences to emerge during re-exudation.

The limited changes observed in the RSA of blackgrass and redroot pigweed in response to water-extracted root exudates may reflect the lower chemical resolution of this extraction, which explained less variation among exudates despite salt removal, potentially masking subtle differences relevant for root growth responses.

#### 4.2.2 Patterns of differential accumulation across neighbor types

Both extracts revealed compounds more accumulated by black oat in response to neighbors. Compared to the “first aqueous extract,” the “second methanolic extract” showed a notable increase in compounds when BO interacted with a neighbor (**Figures 3b, d**) In both experimental sets, intraspecific black oat neighbors triggered the accumulation of more compounds than did heterospecific neighbors. While separate normalization precludes direct quantitative comparison of specific metabolites between datasets, patterns can be compared as both sets share a BO-0/A baseline against which changes were measured. A similar pattern was observed in buckwheat with intraspecific neighbors or redroot pigweed (Eroğlu et al., 2024). This repeated trend may stem from: (1) intense competition between plants with similar resource needs, prompting a negative stress response (Ehlers et al., 2016) or (2) a positive intraspecific interaction, as supported by studies showing cooperative behavior among same-species plants in the rhizosphere (Adler et al., 2018). The first hypothesis (a negative effect due to competition) may be more plausible given the root architecture responses observed in neighbor black oat and neighbor redroot pigweed, which showed reductions in several root traits (**Table 1**).

#### 4.2.3 Enrichment of organic oxygen compounds in response to weed neighbors

In response to the presence of any weedy neighbors, organic oxygen compounds were consistently shown to be more accumulated in black oat exudates (**Figure 4**). Organic oxygen compounds are a vast group of compounds which include alcohols, aldehydes, ketones, ether, carboxylic acids and peroxides (Schmiermund, 2022). More interestingly, organic oxygen compounds are also comprised of well-known components of root exudates such as sugars. Additionally, some specific amino acids (e.g., glutamate, aspartate, and serine) and organic acids are classified as both organic oxygen species and as organic acids and derivatives. These compounds would be preferentially annotated as organic acids and derivatives by our workflow (i.e., they would not cause the observed increase in oxygen compounds). Within the network, many of the organic oxygen compounds were further annotated through level 1 and 2 identifications as carbohydrates, indicating an increase in exudation of sugars and/or glycosides from BO when grown with any weed neighbor.

### 4.3 Molecular networking highlights increased presence of sugars and glycosides within black oat exudate solution in response to redroot pigweed neighbors

The network in **Figure 5** highlights several important findings for the redroot pigweed experimental setup. First, there is an increased accumulation of sugars (annotated as melezitose – though it could be one of many isomers - and an unknown dihexose) and of some unidentifiable amino acids in cluster 3 when black oat is grown with redroot pigweed neighbor. Plants can release certain compounds to change the microbial community for their benefit. They could shape microbial communities to recruit beneficial microbes (Kong and Liu, 2022) and this could be induced by the presence of neighboring plants (Zhou et al., 2023). The sugars and/or amino acids detected in the root exudates might be part of this response. Root exudates with high sugar content may attract beneficial microbes which in turn could help improve plants in the acquisition of nutrients and the suppression of pathogens. Redroot pigweed neighbors may have induced black oat to release sugar-rich exudates to alleviate competition stress (Bais et al., 2006; Ulbrich et al., 2022). Additionally, sugars in the extracts might not be directly from exudated but could result from microbial breakdown of glycosides into simpler sugars as plants were not grown in sterile conditions (Berlemont and Martiny, 2016; Ali et al., 2019).

In cluster 1, three DAC were tentatively annotated as sulphate-containing glycosides (**Figure 5a** and **Supplementary Figure S14**). While they resemble glucosinolates, a defense compound in Brassicales, they lack the sulfur bridge and nitrogen but still share the sugar and sulphate components. Glucosinolates play a role in plant defense, and their breakdown products are toxic compounds utilized in biofumigation (Craine and Dybzinski, 2013; Hanschen et al., 2015). Although these plants are unrelated, making it unlikely that pathways are the same, these sugar sulphates may represent an independently evolved type of protective metabolite. Additionally, the Alliaceae and Asteraceae families also defensively utilize sulfur containing secondary metabolites (Burow et al., 2008). These sugar sulphates would need to be purified or synthesized and applied to weeds to assess this postulation.

Last, cluster 2 contains three aromatic amino acids (**Figure 5b** and **Supplementary Figure S15**). While this seems unrelated to sugar and sugar sulphates, they are linked within the network to an aromatic amino acid with a glycoside. This glycoside is not observable within the aromatic amino acids DACs within the cluster. However, glycosides are often only observed through neutral losses within mass spec fragmentation patterns in positive ionization mode (Qu et al., 2004), as is with the case for alignment ID 1310 which is the related sugar containing aromatic amino acid. Since the parent ions are in such low abundance in the MS2 spectrum, it is not surprising that the fragmentation pattern lacks an observable neutral loss for a sugar, if it contains one. One supporting piece of evidence that these compounds may contain sugars is the annotation from SIRIUS CANOPUS which labels these compounds both as peptides (Level 3 classification) and gives a possible alternative annotation of glycosylamine for Alignment ID 2049 and 2138.

Potential glycosylamines identified in black oat root exudates could influence plant growth. Current research is conflicting on whether plants can actively uptake glycosylamines or not with certain studies showing active uptake (Roberts et al., 1971; Camacho-Pereira et al., 2009) and others arguing that this only occurs at extremely high concentrations (Roberts and Jones, 2012). However, several studies in higher plants have shown that glycosylamines are potent inhibitors of respiration, hexokinase activity, and interfere with the oxidative pentose phosphate pathway, an important source of biosynthetic precursors and reducing power in plants (Sanz and Ullrich, 1989; Hofmann and Roitsch, 2000; Garlick et al., 2002; Roberts and Jones, 2012). Other studies show that glycosylamines are also highly toxic to maize roots even at low concentrations (Roberts et al., 1971). Again, targeted analyses and bioassays using purified compounds are needed to assess their potential effects on neighboring weeds and no conclusions regarding their actual impact in the present study can yet be drawn.

### 4.4 Conclusion

Split-root experiments revealed species specific interactions: black oat inhibited root growth in redroot pigweed but enhanced root development in blackgrass. Moreover, exudates collected from black oat grown alongside redroot pigweed reduced redroot pigweed biomass, while exudates produced in the presence of blackgrass showed minimal impact, highlighting that the suppressive potential of black oat exudates is strongly shaped by the neighbor species.

Black oat also modified its own root characteristics and exudate profiles in response to neighboring plants, with the most pronounced metabolic shifts occurring when growing next to other black oat plants rather than heterospecific weeds. Interactions with redroot pigweed were associated with elevated levels of sugar sulfates and putative glycosylamines, which may be involved in neighbor-dependent root–root interactions, although their precise roles in perception or response modulation remain to be determined.

Although the experimental setup enabled detection of neighbor associated changes, it did not allow for a clear distinction between signaling driven processes and allelopathic activity. Additionally, because the findings stem from controlled split root conditions with simplified substrates, their relevance to naturally complex soil systems remains to be verified.

Future research should focus on validating these patterns in increasingly realistic environments. Structural confirmation and targeted analyses of key metabolites should precede evaluations in soil-based rhizotrons and, eventually, field trials. Expanding the approach to other weed species will help determine how broadly black oat’s metabolic responses apply. Once these mechanisms are better understood, integrating the role of rhizosphere microbial communities will provide deeper insight into biologically mediated weed suppression.

Ultimately, clarifying how black oat modulates its root exudation in response to neighboring plants may guide the selection and management of cover crops with stronger weed suppressive traits, supporting more sustainable, biology driven weed management strategies in agroecosystems.

## Data Availability

The original contributions presented in the study are included in the article/**Supplementary Material**. Further inquiries can be directed to the corresponding author.
