# Supplementary material for: Characterization of black oat root exudates in the presence of interspecific weed species and intraspecific neighbors, and their effects on root traits
Source: Front Plant Sci. 2026 Mar 25;17:1729814. doi: 10.3389/fpls.2026.1729814 (PMC13057449; doi:10.3389/fpls.2026.1729814)
Supplement: Supplementary file 1 [file DataSheet1.docx]

Supplementary Material

# Supplementary Data

## Table S1: A series of compounds across five major classes (small organic acids, amino acids, sugars, flavonoids, and fatty acids) known to exudate from the roots of mostly agricultural plants were selected for assessing the chromatographic and data processing workflows. Each compound’s name, molecular formula, exact mass in Da, and chemical ontology/class is listed. The species each compound is known to be exudated from, the source of each standard, and the purity of each standard is given. One compound, 3,5-Di-tert-butyl-4-hydroxybenzoic acid, is a synthetic and non-naturally occurring compound used as in internal standard.

| Compound name | Molecular formula | Exact mass (Da) | Ontology | Species | Source of chemical standard |
| --- | --- | --- | --- | --- | --- |
| Urea | CH4N2O | 60.0324 | Organic molecular entity | *Arabidopsis thaliana*[1], *Lactuca sativa* L.[2] | Sigma-Aldrich |
| Glycine | C2H5NO2 | 75.0320 | Amino acid | *Arabidopsis* thaliana[1],  Triticum *aestivum* L*.*[3],  *Brachypodium distachyon*[4] | Sigma-Aldrich |
| Butyric acid | C4H8O2 | 88.0524 | Organic acid | *Triticum aestivum* L*.*[3], | Sigma-Aldrich |
| β-AIanine | C3H7NO2 | 89.0477 | Amino acid | *Arabidopsis thaliana*[1],  *Triticum aestivum* L*.*[3], *Brachypodium distachyon*[4], *Zea mays*[5] | Fluka |
| Oxalic acid | C2H2O4 | 89.9953 | Organic acid | *Triticum aestivum* L*.*[3] | Merck Millipore |
| Valeric acid | C5H10O2 | 102.0681 | Organic acid | *Triticum aestivum* L*.*[3] | Fluka |
| Y -Amino butyric acid, GABA | C4H9NO2 | 103.0633 | Amino acid | *Arabidopsis thaliana*[1],  *Triticum aestivum* L*.*[3], *Brachypodium distachyon*[4], *Zea mays*[5] | Sigma-Aldrich |
| Serine | C3H7NO3 | 105.0426 | Amino acid | *Arabidopsis* thaliana[1],  Triticum *aestivum* L*.*[3],  *Brachypodium distachyon*[4] | Sigma-Aldrich |
| Uracil | C4H4N2O2 | 112.0273 | Nucleic Acids | *Arabidopsis thaliana*[1] | Sigma-Aldrich |
| Proline | C5H9NO2 | 115.0633 | Amino acid | *Triticum aestivum* L*.*[3] | Sigma-Aldrich |
| Fumaric acid | C4H4O4 | 116.0110 | Organic acid | *Arabidopsis* thaliana[1],  Triticum *aestivum* L*.*[3] | Fluka |
| Valine | C5H11NO2 | 117.0790 | Amino acid | *Arabidopsis thaliana*[1],  *Triticum aestivum* L*.*[3], *Brachypodium distachyon*[4] | Merck Millipore |
| Succinic acid | C4H6O4 | 118.0266 | Organic acid | *Arabidopsis* thaliana[1],  Triticum *aestivum* L*.*[3] | Fluka |
| Threonine | C4H9NO3 | 119.0582 | Amino acid | *Arabidopsis thaliana*[1],  *Triticum aestivum* L*.*[3], *Brachypodium distachyon*[4], *Zea mays*[5] | Sigma-Aldrich |
| Benzoic acid | C7H6O2 | 122.0368 | Organic acid, Phenol | *Arabidopsis thaliana*[1], *Hordeum vulgare*[6] | Fluka |
| Erythritol | C4H10O4 | 122.0579 | Sugar alcohol | *Arabidopsis thaliana*[1], *Zea mays*[5] | Sigma-Aldrich |
| Pyroglutamic acid | C5H7NO3 | 129.0426 | Amino acid | *Arabidopsis thaliana*[1],  *Sorghum bicolor* L. Moench[7] | Sigma-Aldrich |
| Isoleucine | C6H13NO2 | 131.0946 | Amino acid | *Arabidopsis thaliana*[1],  *Triticum aestivum* L*.*[3], *Brachypodium distachyon*[4], *Zea mays*[5] | Sigma-Aldrich |
| Leucine | C6H13NO2 | 131.0946 | Amino acid | *Triticum aestivum* L*.*[3], *Brachypodium distachyon*[4], *Zea mays*[5] | Sigma-Aldrich |
| Asparagine | C4H8N2O3 | 132.0535 | Amino acid | *Arabidopsis thaliana*[1],  *Triticum aestivum* L*.*[3], *Brachypodium distachyon*[4], *Zea mays*[5] | Sigma-Aldrich |
| Aspartic acid | C4H7NO4 | 133.0375 | Amino acid | *Triticum aestivum* L*.*[3], *Brachypodium distachyon*[4] | Merck Millipore |
| Malic acid | C4H6O5 | 134.0215 | Organic acid | *Triticum aestivum* L*.*[3] | Merck Millipore |
| Threonic acid | C4H8O5 | 136.0372 | Organic acid | *Arabidopsis thaliana*[1], *Sorghum bicolor* L. Moench[7] | Sigma-Aldrich |
| Salicylic acid | C7H6O3 | 138.0317 | Organic acid, Phenol | *Medicago sativa*[8]*,  Triticum aestivum, Avena fatua,* many others[9] | Fluka |
| Glutamine | C5H10N2O3 | 146.0691 | Amino acid | *Triticum aestivum* L*.*[3], *Brachypodium distachyon*[4], *Zea mays*[5] | Sigma-Aldrich |
| Lysine | C6H14N2O2 | 146.1055 | Amino acid | *Triticum aestivum* L*.*[3], *Brachypodium distachyon*[4] | Sigma-Aldrich |
| Glutamic acid | C5H9NO4 | 147.0532 | Amino acid | *Triticum aestivum* L*.*[3],  *Brachypodium distachyon*[4], *Zea mays*[5] | Sigma-Aldrich |
| Methionine | C5H11NO2S | 149.0510 | Amino acid | *Triticum aestivum* L*.*[3], *Brachypodium distachyon*[4] | Sigma-Aldrich |
| Ribose | C5H10O5 | 150.0528 | Sugar | *Arabidopsis* thaliana[1],  Triticum *aestivum* L*.*[3] | Sigma-Aldrich |
| Dopamine | C8H11NO2 | 153.0790 | Phenol | *Brachypodium distachyon*[10] | Sigma-Aldrich |
| Histidine | C6H9N3O2 | 155.0695 | Amino acid | *Brachypodium distachyon*[4], *Oryza sativa*[11] | Fluka |
| Courmaric acid | C9H8O3 | 164.0473 | Organic acid, Phenol | *Medicago sativa*[8]*,*  *Ageratum conyzoides*[12]*,  Eucalyptus spp.*[13]*,  Triticum vulgare*[14]*,  Avena fatua*[15] | Fluka |
| Phenylalanine | C9H11NO2 | 165.0790 | Amino acid, Phenol | *Triticum aestivum* L*.*[3], *Brachypodium distachyon*[4] | Sigma-Aldrich |
| Vanilic acid | C8H8O4 | 168.0423 | Organic acid, Phenol | *Hordeum* vulgare[6],  *Medicago sativa*[8]*, Eucalyptus spp.*[13]*,  Triticum vulgare*[14]*,  Avena fatua*[15]*, Fagopyrum esculentum*[16] | Alfa Aesar |
| Shikimic acid | C7H10O5 | 174.0528 | Organic acid | *Arabidopsis thaliana*[1]*, Oryza sativa*[11] | Sigma-Aldrich |
| Arginine | C6H14N4O2 | 174.1117 | Amino acid | *Brachypodium distachyon*[4], *Oryza sativa*[11] | SAFC Pharma |
| Aldohexose (glucose) | C6H12O6 | 180.0634 | Sugar | *Arabidopsis thaliana*[1]*,*  *Triticum aestivum* L*.*[3], *Zea mays*[5] | Sigma-Aldrich |
| Tyrosine | C9H11NO3 | 181.0739 | Amino acid, Phenol | *Triticum aestivum* L*.*[3], *Brachypodium distachyon*[4], *Zea mays*[5] | Fluka |
| Isocitric acid | C6H8O7 | 192.0270 | Organic acid | *Oryza sativa*[11] | Sigma-Aldrich |
| Citric acid | C6H8O7 | 192.0270 | Organic acid | *Triticum aestivum* L*.*[3],  *Zea mays*[5] | Sigma-Aldrich |
| Ferulic acid | C10H10O4 | 194.0579 | Organic acid, Phenol | *Hordeum vulgare*[6],  *Medicago sativa*[8], *Ageratum conyzoides*[12]*,  Triticum vulgare*[14]*,  Avena fatua*[15]*, Fagopyrum tataricum*[17] | Fluka |
| Syringic acid | C9H10O5 | 198.0528 | Organic acid, Phenol | *Medicago sativa*[8]*, Eucalyptus spp.*[13]*, Triticum vulgare*[14]*,  Avena fatua*[15] | Sigma-Aldrich |
| Lauric acid | C12H24O2 | 200.1776 | Fatty acid | *Arabidopsis thaliana*[1]*, Lactuca sativa* L.[2] | Sigma-Aldrich |
| Tryptophan | C11H12N2O2 | 204.0899 | Amino acid | *Oryza sativa*[11],  *Fagopyrum esculentum*[18] | Sigma-Aldrich |
| Jasmonic acid | C12H18O3 | 210.1256 | Organic acid | *Triticum aestivum, Avena fatua,* many others[9] | Sigma-Aldrich |
| N-acetyl-D-mannosamine | C8H15NO6 | 221.0899 | Sugar | *Arabidopsis thaliana*[1] | Sigma-Aldrich |
| Cystathionine | C7H14N2O4S | 222.0674 | Amino acid | *Triticum aestivum* L*.*[3] | Sigma-Aldrich |
| 3,5-Di-tert-butyl-4-hydroxybenzoic acid | C15H22O3 | 250.1569 | Organic acid, Phenol | None - synthetic internal standard | Sigma-Aldrich |
| Palmitic acid | C16H32O2 | 256.2402 | Fatty acid | *Arabidopsis thaliana*[1]*, Fagopyrum esculentum*[19] | Sigma-Aldrich |
| Stearic acid | C18H36O2 | 284.2715 | Fatty acid | *Arabidopsis thaliana*[1]*, Fagopyrum esculentum*[19] | Sigma-Aldrich |
| Catechin | C15H14O6 | 290.0790 | Flavonoid, Phenol | *Fagopyrum esculentum*[20] | Fluka |
| Epicatechin | C15H14O6 | 290.0790 | Flavonoid, Phenol | *Fagopyrum tataricum*[17] | Sigma-Aldrich |
| Quercetin | C15H10O7 | 302.0427 | Flavonoid, Phenol | *Fagopyrum esculentum*[20] | Sigma-Aldrich |
| Arachidic acid | C20H40O2 | 312.3028 | Fatty acid | *Fagopyrum esculentum*[19] | Sigma-Aldrich |
| Myricetin | C15H10O8 | 318.0376 | Flavonoid, Phenol | *Fagopyrum esculentum*[20] | Sigma-Aldrich |
| Dihexose  (sucrose/maltose) | C12H22O11 | 342.1162 | Sugar | *Arabidopsis thaliana*[1]*, Zea mays*[5]  *Triticum aestivum* L*.*[3] | Sigma-Aldrich |
| α tocopherol | C29H50O2 | 430.3811 | Lipid, Phenol | *Arabidopsis thaliana*[1] | Sigma-Aldrich |
| Rutin | C27H30O16 | 610.1534 | Flavonoid, Phenol, Glycoside | *Fagopyrum tataricum*[17] | Sigma-Aldrich |

## Table S2: Chromatographic gradients & parameters for LC-HRMS/MS data acquisition

| **a:** Single column gradient to PFP column | | |
| --- | --- | --- |
| Time (min) | % A | % B |
| 0.50 | 100 | 0 |
| 4.50 | 95 | 5 |
| 5.00 | 60 | 40 |
| 8.00 | 60 | 40 |
| 10.50 | 0 | 100 |
| 12.50 | 0 | 100 |
| 13.50 | 100 | 0 |
| 15.50 | 100 | 0 |

| **b:** Dual column pump 1 gradient to PFP column | | |  | **c:** Dual column pump 2 gradient PGC column | | |
| --- | --- | --- | --- | --- | --- | --- |
| Time (min) | % A | % B |  | Time (min) | % A | % B |
| 3.00 | 100 | 0 |  | 2.50 | 100 | 0 |
| 7.00 | 95 | 5 |  | 6.50 | 40 | 60 |
| 7.50 | 60 | 40 |  | 9.00 | 40 | 60 |
| 10.50 | 60 | 40 |  | 11.00 | 0 | 100 |
| 13.00 | 0 | 100 |  | 13.00 | 0 | 100 |
| 15.00 | 0 | 100 |  | 14.00 | 100 | 0 |
| 16.00 | 100 | 0 |  | 20.00 | 100 | 0 |
| 20.00 | 100 | 0 |  |  |  |  |

**d:** Other chromatographic and mass spectrometric parameters

| Software: MassHunter acquisition software (version 10.1) | |
| --- | --- |
| Single column and dual column pump 1 chromatographic conditions | |
| Stationary phase | Sigma-Aldrich Discovery HS F5  (150 x 2.1 mm, 3 μm particle size) |
| Mobile phase A | 99.9% v/v H2O, 0.1% v/v formic acid |
| Mobile phase B | 99.9% v/v methanol, 0.1% v/v formic acid |
| Flow rate (ml min^-1^) | 0.350 |
| Column oven T (°C) | 50 |
| Injection volume (μl) | 5 |
| Dual column pump 2 chromatographic conditions | |
| Stationary phase | Thermo Scientific Hypercarb column (2.1 × 150 mm, 5 μm particle size |
| Mobile phase A | 99.9% v/v H2O, 0.1% v/v formic acid |
| Mobile phase B | 99.9% v/v acetonitrile, 0.1% v/v formic acid |
| Flow rate (mL min^-1^) | 0.350 |
| Column oven T (°C) | 50 |
| Injection volume (μl) | - |
| Source parameters | |
| Gas Temp (°C) | 300 |
| Gas Flow (L min^-1^) | 8 |
| Nebulizer (psig) | 35 |
| Sheath Gas Temp (L min^-1^) | 300 |
| Sheath Gas Flow (L min^-1^) | 11 |
| VCap | 3500 |
| Nozzle Voltage (V) | 500 |
| Fragmentor (V) | 360 |
| Skimmer 1 | 65 |
| Octopole RF Peak | 750 |
| Scan parameters: Acquisition Mode AutoMS2 (DDA) | |
| MS Min Range (m/z) | 50 |
| MS Max Range (m/z) | 1700 |
| MS Scan Rate (spectra sec^-1^) | 5.00 |
| Resolution MS1 and MS2 | ~17.5 K @ m/z 322 |
| MS/MS Min Range (m/z) | 50 |
| MS/MS Max Range (m/z) | 1700 |
| MS/MS Scan Rate (spectra sec^-1^) | 7.00 |
| Isolation Width MS/MS | Narrow (~1.3 amu) |
| Minimum cycle time (Hz) | 1 |
| Maximum cycle time (Hz) | 5 |
| Collision Energy Table | |
| Mass | Z1 |
| 0 | 5 |
| 300 | 10 |
| 1700 | 45 |
| Precursor Selection | |
| Max Precursors Per Cycle | 5 |
| Threshold (Abs) | 2000 |
| Threshold (Rel %) | 0.010 |
| Precursor abundance based scan speed | No |
| Purity Stringency (%) | 100.000 |
| Purity Cutoff (%) | 30.000 |
| Isotope Model | Common |
| Active exclusion enabled | Yes |
| Active exclusion excluded after (spectra) | 3 |
| Active exclusion released after (min) | 0.30 |
| Sort precursors | By abundance only |
| Reference Masses | |
| A nano pump for reference mass solution (1260 Infinity) led to a secondary sprayer | |
| Negative ionization | Positive ionization |
| 119.0363 m/z | 121.0509m/z |
| 966.0007 m/z | 922.0098m/z |

## Table S3: MS DIAL preprocessing parameters

| MS-DIAL ver. 4.9.221218 | | | |
| --- | --- | --- | --- |
| MS1 Data type | Centroid | | |
| MS2 Data type | Centroid | | |
| Ion mode | Negative & Positive | | |
| Target | Metabolomics | | |
| Mode | ddMSMS | | |
| Data collection parameters | | | |
| Retention time begin (min) | Single: 1.6 | | Dual: 2.5 |
| Retention time end (min) | Single: 15.5 | | Dual: 21.0 |
| Mass range begin (m/z) | 50 | | |
| Mass range end (m/z) | 1700 | | |
| MS2 mass range begin (m/z) | 50 | | |
| MS2 mass range end (m/z) | 1700 | | |
| Centroid parameters | | | |
| MS1 tolerance (Da) | 0.01 | | |
| MS2 tolerance (Da) | 0.025 | | |
| Isotope recognition | | | |
| Maximum charged number | 2 | | |
| Peak detection parameters | | | |
| Smoothing method | Linear Weighted Moving Average | | |
| Smoothing level (points) | 6 | | |
| Minimum peak width (points) | 6 | | |
| Minimum peak height (counts) | 1000 | | |
| Peak spotting parameters | | | |
| Mass slice width | 0.1 | | |
| Deconvolution parameters | | | |
| Sigma window value | | 0.5 | |
| MS2 Dec amplitude cut off (counts) | | 0 | |
| Exclude after precursor | | TRUE | |
| Keep isotope until (Da) | | 0.5 | |
| Keep original precursor isotopes | | FALSE | |
| MSP file and MS/MS identification setting | | | |
| Retention time tolerance (min) | | 1 | |
| Accurate mass tolerance MS1 (Da) | | 0.02 | |
| Accurate mass tolerance MS2 (Da) | | 0.03 | |
| Identification score cut off (%) | | 80 | |
| Using retention time for scoring | | TRUE | |
| Using retention time for filtering | | FALSE | |
| Adduct ion setting | | | |
| Negative ionization | | **Positive ionization** | |
| [M-H]- | | [M+H]+ | |
| [M+Cl]- | | [M+Na]+ | |
| [M+FA-H]- | | [M+K]+ | |
| [M-H2O-H]- | | [M+NH4]+ | |
| [M-CO2-H]- | | [M-NH2]+ | |
|  | | [M+H-H2O]+ | |
|  | | [M+H-CO2]+ | |
| Alignment parameters setting | | | |
| Retention time tolerance (min) | | 0.2 | |
| MS1 tolerance (Da) | | 0.015 | |
| Retention time factor | | 0.5 | |
| MS1 factor | | 0.5 | |
| Peak count filter (%) | | 0 | |
| N detected in at least one group (%) | | 60 | |
| Remove feature based on peak height fold-change | | TRUE | |
| Sample average (height, counts) /  blank average (height, counts) | | 5 | |
| Keep identified and annotated metabolites | | TRUE | |
| Keep removable features and assign the tag for checking | | TRUE | |
| Gap filling by compulsion | | TRUE | |

## Table S4: Diagnostic ion for identification of avenaol, scopoletin, and scopoline

| **Compound** | **Ionization mode** | **Parent mass** | **Product 1** | **Product 2** | **Product 3** |
| --- | --- | --- | --- | --- | --- |
| avenaol | Positive | 377.160 | 97 | 235 | 263 |
| scopoletin | Positive | 193.050 | 122.04 | 133.03 | 178.03 |
| scopoletin | Negative | 191.035 | 104.03 | 148.02 | 176.01 |
| scopoline | Positive | 355.102 | 133.03 | 178.026 | 193.05 |
| scopoline | Negative | 353.088 | 176.01 | 191.04 |  |

## Table S5: Differentially accumulated compounds in the A compartment of black oat grown with intraspecific neighbors (BO-BO/A) compared to black oat grown alone (BO-0/A) in the redroot pigweed experimental set (P). Data are from the second 24-hour methanolic re-exudation extract. Compounds were identified using multiple confidence levels according to the Schymanski scale: Level 1 identification was based on matching to authentic standards (annotation "w/o MS2" indicates no confident MS2 spectral match); Level 2a identification was obtained from spectral library matching via MS-FINDER and GNPS; Level 2b identification was predicted using MS-FINDER and SIRIUS CSI:FingerID. Statistical significance was determined by Welch's t-test (FDR-corrected p < 0.05) and |log2 fold change| > 0.6.

| Alignment ID | Ionization Mode | Level 1 Identification | Level 2a Identification | Level 2b Identification | Average Rt(min) | Average Mz | BO-0/A | BO-BO/A | p.value | fdr adjusted p.value | Fold Change | Log2 fold change |
| --- | --- | --- | --- | --- | --- | --- | --- | --- | --- | --- | --- | --- |
| 2 | Negative | Unknown | Unknown | Unknown | 11.89 | 57.03 | 58.06 | 97.43 | 0 | 0.01 | 1.68 | 0.75 |
| 4 | Negative | Unknown | Unknown | Unknown | 11.89 | 59.01 | 160.23 | 260.56 | 0 | 0.04 | 1.63 | 0.7 |
| 18 | Negative | Unknown | Unknown | Unknown | 4.89 | 71.01 | 110.35 | 184.5 | 0.01 | 0.05 | 1.67 | 0.74 |
| 69 | Negative | Unknown | Unknown | Unknown | 11.89 | 99.04 | 146.18 | 293.9 | 0 | 0 | 2.01 | 1.01 |
| 129 | Negative | Unknown | Unknown | Unknown | 12.46 | 121 | 100.04 | 214.07 | 0 | 0 | 2.14 | 1.1 |
| 136 | Negative | Unknown | Unknown | Unknown | 11.89 | 125.02 | 75.38 | 129.65 | 0 | 0 | 1.72 | 0.78 |
| 197 | Negative | Unknown | Unknown | Unknown | 9.13 | 150.06 | 17.08 | 28.35 | 0.01 | 0.03 | 1.66 | 0.73 |
| 221 | Negative | Unknown | Unknown | Unknown | 12.38 | 162.02 | 43.11 | 81.18 | 0 | 0 | 1.88 | 0.91 |
| 237 | Negative | Unknown | Unknown | Unknown | 10.47 | 167.04 | 7.36 | 12.26 | 0.01 | 0.02 | 1.67 | 0.74 |
| 287 | Negative | Unknown | Unknown | L-Tyrosine | 6.87 | 180.07 | 74.97 | 134.94 | 0.01 | 0.04 | 1.8 | 0.85 |
| 322 | Negative | Unknown | D-(-)-Quinic acid; LC-ESI-QTOF; MS2; CE | Quinic acid | 5.18 | 191.06 | 6753.04 | 12377.94 | 0.01 | 0.04 | 1.83 | 0.87 |
| 335 | Negative | Unknown | Unknown | Unknown | 4.7 | 195.05 | 102.23 | 176.19 | 0 | 0.01 | 1.72 | 0.79 |
| 336 | Negative | Unknown | 1726059 - 40.0 eV | Galactonic acid | 4.98 | 195.05 | 559.81 | 1292.16 | 0 | 0.02 | 2.31 | 1.21 |
| 414 | Negative | Unknown | Unknown | Unknown | 5.09 | 220.08 | 395.67 | 1025.7 | 0 | 0 | 2.59 | 1.37 |
| 425 | Negative | Unknown | Unknown | Unknown | 2.9 | 224.09 | 45.35 | 115.01 | 0.01 | 0.01 | 2.54 | 1.34 |
| 429 | Negative | Unknown | Unknown | Unknown | 4.89 | 227 | 53.7 | 89.44 | 0 | 0.02 | 1.67 | 0.74 |
| 508 | Negative | Unknown | Unknown | Unknown | 4.99 | 253.09 | 72.82 | 182.99 | 0 | 0.01 | 2.51 | 1.33 |
| 528 | Negative | Unknown | Unknown | Unknown | 4.53 | 260.02 | 75.19 | 136.47 | 0 | 0.01 | 1.82 | 0.86 |
| 536 | Negative | Unknown | Unknown | Unknown | 10.69 | 263.02 | 65.82 | 103.89 | 0 | 0.01 | 1.58 | 0.66 |
| 544 | Negative | Unknown | Unknown | Unknown | 9.02 | 267.03 | 200.07 | 339.1 | 0.01 | 0.03 | 1.69 | 0.76 |
| 604 | Negative | Unknown | Unknown | Unknown | 2.94 | 287.02 | 30.47 | 94.34 | 0.01 | 0.04 | 3.1 | 1.63 |
| 622 | Negative | Unknown | Unknown | Unknown | 9.39 | 293.09 | 270.47 | 466.67 | 0.01 | 0.03 | 1.73 | 0.79 |
| 662 | Negative | Unknown | Unknown | Unknown | 12.4 | 307.12 | 205.07 | 330.74 | 0.01 | 0.03 | 1.61 | 0.69 |
| 688 | Negative | Unknown | Unknown | Unknown | 3.51 | 315.08 | 13.81 | 50.08 | 0 | 0.02 | 3.63 | 1.86 |
| 704 | Negative | Unknown | Unknown | Unknown | 12.47 | 321.1 | 514.21 | 1126.01 | 0 | 0 | 2.19 | 1.13 |
| 731 | Negative | Unknown | Unknown | Unknown | 3.53 | 331.05 | 97.27 | 262.34 | 0.01 | 0.05 | 2.7 | 1.43 |
| 741 | Negative | Unknown | Unknown | Unknown | 4.68 | 334.13 | 11.45 | 21.02 | 0 | 0 | 1.84 | 0.88 |
| 749 | Negative | Unknown | Unknown | Unknown | 9.35 | 337.11 | 152.03 | 248.11 | 0 | 0.01 | 1.63 | 0.71 |
| 772 | Negative | Unknown | Unknown | Unknown | 11.38 | 343.15 | 48.44 | 103.6 | 0 | 0 | 2.14 | 1.1 |
| 776 | Negative | Unknown | Unknown | Unknown | 2.89 | 344.96 | 132.69 | 265.58 | 0.02 | 0.05 | 2 | 1 |
| 787 | Negative | Unknown | Unknown | Unknown | 12.68 | 349.19 | 135.45 | 295.56 | 0.01 | 0.03 | 2.18 | 1.13 |
| 798 | Negative | Unknown | Unknown | Unknown | 11.38 | 353.18 | 81.34 | 184.05 | 0 | 0.01 | 2.26 | 1.18 |
| 802 | Negative | Unknown | Unknown | Unknown | 9.85 | 355.05 | 37.5 | 70 | 0 | 0.01 | 1.87 | 0.9 |
| 826 | Negative | Unknown | Unknown | Unknown | 12.55 | 365.05 | 69.51 | 116.9 | 0.01 | 0.02 | 1.68 | 0.75 |
| 829 | Negative | Unknown | Unknown | Unknown | 11.46 | 365.14 | 137.07 | 247.62 | 0 | 0.01 | 1.81 | 0.85 |
| 839 | Negative | Unknown | Unknown | Unknown | 9.08 | 371.1 | 158.97 | 265.01 | 0.01 | 0.02 | 1.67 | 0.74 |
| 843 | Negative | Unknown | Unknown | Unknown | 9.03 | 373.08 | 3535.57 | 5654.89 | 0 | 0.01 | 1.6 | 0.68 |
| 866 | Negative | Unknown | Unknown | Unknown | 10.32 | 379.16 | 258.68 | 426.1 | 0.01 | 0.02 | 1.65 | 0.72 |
| 874 | Negative | Unknown | Unknown | Unknown | 5.17 | 383.12 | 65.12 | 177.47 | 0.01 | 0.03 | 2.73 | 1.45 |
| 888 | Negative | Unknown | Unknown | Unknown | 11.68 | 387.09 | 51.25 | 93.17 | 0 | 0 | 1.82 | 0.86 |
| 889 | Negative | Unknown | Unknown | Unknown | 9.14 | 387.1 | 588.64 | 991.05 | 0.01 | 0.02 | 1.68 | 0.75 |
| 891 | Negative | Unknown | alpha,alpha-Trehalose - 40.0 eV | Unknown | 4.98 | 387.12 | 1949.15 | 3256.92 | 0.01 | 0.04 | 1.67 | 0.74 |
| 893 | Negative | Unknown | Unknown | Unknown | 9.39 | 387.13 | 1073.43 | 1945.77 | 0.01 | 0.02 | 1.81 | 0.86 |
| 917 | Negative | Unknown | Unknown | Unknown | 11.54 | 393.18 | 78.41 | 157.94 | 0.01 | 0.02 | 2.01 | 1.01 |
| 934 | Negative | Unknown | Unknown | Unknown | 9.03 | 403.08 | 41.92 | 95.15 | 0 | 0.02 | 2.27 | 1.18 |
| 935 | Negative | Unknown | Unknown | PubChem:(46222441) | 8.49 | 403.09 | 144.79 | 296.08 | 0.02 | 0.04 | 2.04 | 1.03 |
| 942 | Negative | Unknown | Unknown | Unknown | 9.03 | 405.05 | 41.7 | 71.12 | 0 | 0.01 | 1.71 | 0.77 |
| 960 | Negative | Unknown | Unknown | COCONUT:(CNP0159349 CNP0218833);Natural Products:(UNPD202200);PubChem:(38363079 38363084 45360328 44715457 125416071 125416072 125416073 125416074);SuperNatural:(SN00032625 SN00030299 SN00032624 SN00030300 SN00032623 SN00032622);ZINC bio:(ZINC31169353 ZINC31169357 ZINC35454681 ZINC35454685 ZINC35454688 ZINC35454690);Training Set | 11.89 | 413.15 | 4009.66 | 7535.58 | 0 | 0.01 | 1.88 | 0.91 |
| 961 | Negative | Unknown | Unknown | COCONUT:(CNP0159349 CNP0218833);Natural Products:(UNPD202200);PubChem:(38363079 38363084 45360328 44715457 125416071 125416072 125416073 125416074);SuperNatural:(SN00032625 SN00030299 SN00032624 SN00030300 SN00032623 SN00032622);ZINC bio:(ZINC31169353 ZINC31169357 ZINC35454681 ZINC35454685 ZINC35454688 ZINC35454690);Training Set | 11.05 | 413.15 | 60.28 | 112.55 | 0 | 0.01 | 1.87 | 0.9 |
| 962 | Negative | Unknown | Unknown | Unknown | 10.55 | 413.15 | 35.24 | 61.29 | 0 | 0 | 1.74 | 0.8 |
| 966 | Negative | Unknown | Unknown | Unknown | 5.2 | 415.14 | 306.35 | 671.59 | 0 | 0.01 | 2.19 | 1.13 |
| 972 | Negative | Unknown | Unknown | Unknown | 9.08 | 417.11 | 159.29 | 266.93 | 0 | 0.01 | 1.68 | 0.74 |
| 988 | Negative | Unknown | Unknown | Unknown | 9.15 | 421.08 | 84.13 | 160.65 | 0 | 0.01 | 1.91 | 0.93 |
| 1013 | Negative | Unknown | Unknown | Unknown | 9.2 | 429.14 | 15.87 | 32.41 | 0.01 | 0.01 | 2.04 | 1.03 |
| 1018 | Negative | Unknown | Unknown | Unknown | 12.59 | 431.1 | 42.92 | 78.13 | 0.01 | 0.03 | 1.82 | 0.86 |
| 1034 | Negative | Unknown | Unknown | Unknown | 12.71 | 435.19 | 937.41 | 1936.53 | 0 | 0.01 | 2.07 | 1.05 |
| 1061 | Negative | Unknown | Unknown | Unknown | 9.4 | 447.11 | 17.13 | 34.28 | 0.02 | 0.04 | 2 | 1 |
| 1064 | Negative | Unknown | Unknown | Unknown | 9.81 | 447.15 | 458.97 | 782.87 | 0.02 | 0.04 | 1.71 | 0.77 |
| 1065 | Negative | Unknown | Unknown | Unknown | 9.15 | 447.15 | 267.19 | 492.42 | 0.01 | 0.02 | 1.84 | 0.88 |
| 1070 | Negative | Unknown | Unknown | Unknown | 12.31 | 449.2 | 106.14 | 190.03 | 0 | 0 | 1.79 | 0.84 |
| 1071 | Negative | Unknown | Unknown | Unknown | 10.98 | 449.2 | 50.47 | 85.95 | 0 | 0.01 | 1.7 | 0.77 |
| 1079 | Negative | Unknown | Unknown | Unknown | 12.94 | 451.22 | 366.56 | 964.78 | 0 | 0.02 | 2.63 | 1.4 |
| 1085 | Negative | Unknown | Unknown | Unknown | 11.01 | 453.2 | 45.33 | 121.38 | 0 | 0.02 | 2.68 | 1.42 |
| 1088 | Negative | Unknown | Unknown | Unknown | 9.93 | 455.12 | 120.04 | 186.29 | 0.01 | 0.04 | 1.55 | 0.63 |
| 1089 | Negative | Unknown | Unknown | Unknown | 9.47 | 455.12 | 547.17 | 960.46 | 0.01 | 0.03 | 1.76 | 0.81 |
| 1092 | Negative | Unknown | Unknown | Unknown | 12.86 | 456.15 | 83.74 | 129.58 | 0 | 0.01 | 1.55 | 0.63 |
| 1094 | Negative | Unknown | Unknown | Unknown | 12.51 | 457.17 | 268.4 | 454.43 | 0 | 0.01 | 1.69 | 0.76 |
| 1105 | Negative | Unknown | Unknown | Unknown | 9.49 | 461.13 | 36.49 | 64.02 | 0.01 | 0.04 | 1.75 | 0.81 |
| 1113 | Negative | Unknown | Unknown | Unknown | 9.54 | 463.14 | 139.78 | 232.06 | 0.01 | 0.04 | 1.66 | 0.73 |
| 1119 | Negative | Unknown | Unknown | Unknown | 13.13 | 465.25 | 86.96 | 164.57 | 0.01 | 0.02 | 1.89 | 0.92 |
| 1123 | Negative | Unknown | Unknown | Unknown | 9.11 | 467.05 | 30.3 | 54.66 | 0 | 0.01 | 1.8 | 0.85 |
| 1125 | Negative | Unknown | Unknown | Unknown | 12.77 | 467.21 | 416.56 | 807.86 | 0.01 | 0.01 | 1.94 | 0.96 |
| 1135 | Negative | Unknown | Unknown | Unknown | 10.42 | 472.1 | 35.86 | 59.37 | 0.02 | 0.03 | 1.66 | 0.73 |
| 1137 | Negative | Unknown | Unknown | Unknown | 10.34 | 473.17 | 69.22 | 112.64 | 0.01 | 0.04 | 1.63 | 0.7 |
| 1142 | Negative | Unknown | Unknown | Unknown | 11.9 | 476.14 | 54.78 | 111.44 | 0 | 0.01 | 2.03 | 1.02 |
| 1146 | Negative | Unknown | Unknown | Unknown | 12.64 | 477.14 | 43.37 | 70.95 | 0.02 | 0.05 | 1.64 | 0.71 |
| 1151 | Negative | Unknown | Unknown | Unknown | 10.32 | 479.09 | 42.79 | 92.43 | 0.02 | 0.04 | 2.16 | 1.11 |
| 1158 | Negative | Unknown | Unknown | Unknown | 12.51 | 482.19 | 58.08 | 88.28 | 0.01 | 0.03 | 1.52 | 0.6 |
| 1161 | Negative | Unknown | Unknown | Unknown | 10.12 | 483.21 | 69.55 | 155.71 | 0 | 0.02 | 2.24 | 1.16 |
| 1191 | Negative | Unknown | Unknown | Unknown | 10.04 | 493.16 | 4.59 | 13.26 | 0.01 | 0.03 | 2.89 | 1.53 |
| 1193 | Negative | Unknown | Unknown | Unknown | 13.21 | 493.23 | 213.84 | 430.17 | 0.01 | 0.03 | 2.01 | 1.01 |
| 1206 | Negative | Unknown | Unknown | Unknown | 5.32 | 503.16 | 2704.38 | 5838.8 | 0 | 0.01 | 2.16 | 1.11 |
| 1207 | Negative | Unknown | Unknown | Unknown | 11.97 | 503.17 | 36.05 | 78.3 | 0 | 0.01 | 2.17 | 1.12 |
| 1214 | Negative | Unknown | Unknown | Unknown | 12.58 | 507.21 | 1545.3 | 3292.72 | 0 | 0 | 2.13 | 1.09 |
| 1220 | Negative | Unknown | Unknown | Unknown | 12.77 | 511.11 | 99.32 | 185.49 | 0 | 0.01 | 1.87 | 0.9 |
| 1225 | Negative | Unknown | Unknown | Unknown | 11.88 | 513.07 | 661.15 | 1442.13 | 0.01 | 0.03 | 2.18 | 1.13 |
| 1230 | Negative | Unknown | Unknown | Unknown | 4.96 | 515.12 | 25.1 | 61.98 | 0 | 0.01 | 2.47 | 1.3 |
| 1242 | Negative | Unknown | Unknown | Unknown | 13.28 | 519.24 | 52.95 | 103.99 | 0.01 | 0.04 | 1.96 | 0.97 |
| 1249 | Negative | Unknown | Unknown | Unknown | 9.23 | 523.17 | 48.24 | 81.29 | 0.01 | 0.04 | 1.69 | 0.75 |
| 1258 | Negative | Unknown | Unknown | Unknown | 10.36 | 533.16 | 46.32 | 89.14 | 0 | 0 | 1.92 | 0.94 |
| 1259 | Negative | Unknown | Unknown | Unknown | 9.76 | 533.17 | 261.98 | 505.07 | 0.01 | 0.01 | 1.93 | 0.95 |
| 1260 | Negative | Unknown | Unknown | Unknown | 5.16 | 533.17 | 509.46 | 1484.26 | 0 | 0 | 2.91 | 1.54 |
| 1268 | Negative | Unknown | Unknown | Unknown | 13.45 | 537.25 | 131.66 | 224.45 | 0.01 | 0.03 | 1.7 | 0.77 |
| 1310 | Negative | Unknown | Unknown | Unknown | 9.81 | 561.22 | 40.3 | 65.14 | 0.01 | 0.01 | 1.62 | 0.69 |
| 1311 | Negative | Unknown | Unknown | Unknown | 5.1 | 562.2 | 41.51 | 127.99 | 0 | 0.01 | 3.08 | 1.62 |
| 1329 | Negative | Unknown | Unknown | Unknown | 11.28 | 575.2 | 58.16 | 100.75 | 0 | 0 | 1.73 | 0.79 |
| 1339 | Negative | Unknown | Unknown | Unknown | 10.63 | 589.18 | 43.81 | 69.09 | 0.01 | 0.02 | 1.58 | 0.66 |
| 1348 | Negative | Unknown | Unknown | Unknown | 5.32 | 601.13 | 264.42 | 568.59 | 0.01 | 0.04 | 2.15 | 1.1 |
| 1356 | Negative | Unknown | Unknown | Unknown | 5.2 | 607.21 | 29.37 | 107.1 | 0.01 | 0.02 | 3.65 | 1.87 |
| 1370 | Negative | Unknown | Unknown | Unknown | 5.32 | 621.19 | 52.46 | 111.66 | 0.01 | 0.02 | 2.13 | 1.09 |
| 1390 | Negative | Unknown | Unknown | Unknown | 5.49 | 637.18 | 721.09 | 1538.39 | 0 | 0.02 | 2.13 | 1.09 |
| 1396 | Negative | Unknown | Unknown | Unknown | 13.58 | 641.19 | 5.57 | 9.07 | 0.01 | 0.03 | 1.63 | 0.71 |
| 1399 | Negative | Unknown | Unknown | Unknown | 13.05 | 643.2 | 85.85 | 137.1 | 0 | 0 | 1.6 | 0.68 |
| 1414 | Negative | Unknown | Unknown | Unknown | 13.19 | 661.18 | 147.5 | 227.05 | 0.01 | 0.02 | 1.54 | 0.62 |
| 1415 | Negative | Unknown | Unknown | Unknown | 12.63 | 661.18 | 54.26 | 129.41 | 0 | 0 | 2.39 | 1.25 |
| 1432 | Negative | Unknown | Unknown | Unknown | 8.03 | 668.2 | 29.69 | 69.56 | 0.02 | 0.04 | 2.34 | 1.23 |
| 1446 | Negative | Unknown | Unknown | Unknown | 13.56 | 675.19 | 188.99 | 320.54 | 0 | 0 | 1.7 | 0.76 |
| 1447 | Negative | Unknown | Unknown | Unknown | 13.27 | 675.19 | 759.05 | 1199.5 | 0 | 0.01 | 1.58 | 0.66 |
| 1451 | Negative | Unknown | Unknown | Unknown | 5.5 | 679.22 | 21.19 | 88.37 | 0 | 0.02 | 4.17 | 2.06 |
| 1458 | Negative | Unknown | Unknown | Unknown | 5.09 | 683.23 | 3001.75 | 7380.36 | 0 | 0.03 | 2.46 | 1.3 |
| 1461 | Negative | Unknown | Unknown | Unknown | 13.26 | 685.18 | 216.15 | 371.79 | 0 | 0 | 1.72 | 0.78 |
| 1466 | Negative | Unknown | Unknown | Unknown | 11.25 | 687.19 | 102.71 | 209.85 | 0 | 0 | 2.04 | 1.03 |
| 1467 | Negative | Unknown | Unknown | Unknown | 13.05 | 687.19 | 1336.43 | 2259.57 | 0 | 0 | 1.69 | 0.76 |
| 1486 | Negative | Unknown | Unknown | Unknown | 12.6 | 697.24 | 108.02 | 222.64 | 0 | 0.01 | 2.06 | 1.04 |
| 1492 | Negative | Unknown | Unknown | Unknown | 12.87 | 703.19 | 158.86 | 246.8 | 0.01 | 0.02 | 1.55 | 0.64 |
| 1494 | Negative | Unknown | Unknown | Unknown | 10.01 | 703.26 | 30.6 | 65.33 | 0.01 | 0.02 | 2.14 | 1.09 |
| 1497 | Negative | Unknown | Unknown | Unknown | 10.27 | 707.18 | 147.9 | 244.72 | 0.01 | 0.02 | 1.65 | 0.73 |
| 1507 | Negative | Unknown | Unknown | Unknown | 11.88 | 711.3 | 25.52 | 49.32 | 0 | 0 | 1.93 | 0.95 |
| 1539 | Negative | Unknown | Unknown | Unknown | 13.05 | 733.2 | 58.43 | 92.88 | 0 | 0.01 | 1.59 | 0.67 |
| 1547 | Negative | Unknown | Unknown | Unknown | 13.27 | 738.19 | 87.08 | 136.79 | 0.01 | 0.02 | 1.57 | 0.65 |
| 1610 | Negative | Unknown | Unknown | Unknown | 5.09 | 781.2 | 60.68 | 147.66 | 0.01 | 0.05 | 2.43 | 1.28 |
| 1611 | Negative | Unknown | Unknown | Unknown | 12.88 | 781.22 | 53.35 | 88.52 | 0 | 0.01 | 1.66 | 0.73 |
| 1623 | Negative | Unknown | Unknown | Unknown | 10.41 | 795.29 | 19.72 | 43.56 | 0.01 | 0.03 | 2.21 | 1.14 |
| 1633 | Negative | Unknown | Unknown | Unknown | 13.05 | 805.22 | 83.74 | 132.19 | 0 | 0 | 1.58 | 0.66 |
| 1635 | Negative | Unknown | Unknown | Unknown | 11.08 | 808.21 | 67.59 | 158.86 | 0.01 | 0.02 | 2.35 | 1.23 |
| 1636 | Negative | Unknown | Unknown | Unknown | 12.57 | 808.21 | 44.65 | 79.7 | 0.03 | 0.05 | 1.79 | 0.84 |
| 1664 | Negative | Unknown | Unknown | Unknown | 10.03 | 826.3 | 26.33 | 47.14 | 0.02 | 0.05 | 1.79 | 0.84 |
| 1691 | Negative | Unknown | Unknown | Unknown | 12.58 | 845.45 | 54.65 | 87.43 | 0.02 | 0.04 | 1.6 | 0.68 |
| 1697 | Negative | Unknown | Unknown | Unknown | 12.64 | 857.25 | 83.52 | 155.23 | 0 | 0.01 | 1.86 | 0.89 |
| 1730 | Negative | Unknown | Unknown | Unknown | 13.33 | 943.29 | 226.06 | 416.94 | 0.01 | 0.02 | 1.84 | 0.88 |
| 1764 | Negative | Unknown | Unknown | Unknown | 14.65 | 985.61 | 16.25 | 43.33 | 0.01 | 0.04 | 2.67 | 1.42 |
| 1788 | Negative | Unknown | Unknown | Unknown | 14.69 | 1033.54 | 20.62 | 43.43 | 0.01 | 0.03 | 2.11 | 1.07 |
| 1808 | Negative | Unknown | Unknown | Unknown | 13.25 | 1105.34 | 58.88 | 94.58 | 0.01 | 0.04 | 1.61 | 0.68 |
| 1809 | Negative | Unknown | Unknown | Unknown | 13.09 | 1109.41 | 64.05 | 140.99 | 0 | 0 | 2.2 | 1.14 |
| 1825 | Negative | Unknown | Unknown | Unknown | 13.04 | 1375.39 | 37.25 | 89.75 | 0 | 0 | 2.41 | 1.27 |
| 180 | Positive | Unknown | Unknown | Unknown | 5.03 | 110.07 | 115.79 | 298.11 | 0 | 0.02 | 2.57 | 1.36 |
| 216 | Positive | Unknown | Unknown | Unknown | 4.88 | 120.07 | 32.56 | 54.98 | 0.01 | 0.04 | 1.69 | 0.76 |
| 273 | Positive | Unknown | Unknown | Unknown | 4.7 | 138.05 | 29.62 | 58.68 | 0.01 | 0.02 | 1.98 | 0.99 |
| 274 | Positive | Unknown | Unknown | 2-Aminobenzoic acid | 4.97 | 138.05 | 650.74 | 1316.18 | 0.01 | 0.02 | 2.02 | 1.02 |
| 296 | Positive | Unknown | Unknown | Unknown | 11.88 | 145.05 | 67.62 | 114.2 | 0 | 0.01 | 1.69 | 0.76 |
| 308 | Positive | Unknown | Unknown | Unknown | 9.66 | 149.12 | 28.11 | 68.45 | 0 | 0.02 | 2.44 | 1.28 |
| 343 | Positive | Unknown | Unknown | Unknown | 3.69 | 160.1 | 32.95 | 58.4 | 0.01 | 0.03 | 1.77 | 0.83 |
| 352 | Positive | Unknown | Unknown | Unknown | 4.96 | 163.06 | 115.47 | 229.01 | 0 | 0.05 | 1.98 | 0.99 |
| 353 | Positive | Unknown | Unknown | Unknown | 4.86 | 163.06 | 342.25 | 638.95 | 0 | 0.04 | 1.87 | 0.9 |
| 354 | Positive | Unknown | Unknown | Unknown | 11.87 | 163.06 | 95.1 | 165.21 | 0 | 0.01 | 1.74 | 0.8 |
| 363 | Positive | Unknown | Unknown | Sabiden | 10.5 | 166.09 | 810.86 | 1308.36 | 0 | 0.03 | 1.61 | 0.69 |
| 386 | Positive | Unknown | Unknown | Unknown | 4.99 | 176.09 | 120.45 | 266.88 | 0 | 0 | 2.22 | 1.15 |
| 405 | Positive | Unknown | Unknown | Unknown | 5.03 | 181.05 | 40.14 | 84.83 | 0 | 0.01 | 2.11 | 1.08 |
| 422 | Positive | Unknown | Unknown | Unknown | 3.54 | 185.04 | 26.28 | 51.47 | 0.01 | 0.03 | 1.96 | 0.97 |
| 423 | Positive | Unknown | Unknown | Unknown | 5.02 | 185.09 | 42.34 | 70.69 | 0.01 | 0.03 | 1.67 | 0.74 |
| 426 | Positive | Unknown | Unknown | Unknown | 5 | 186.08 | 195.77 | 366.07 | 0 | 0 | 1.87 | 0.9 |
| 445 | Positive | Unknown | Unknown | Unknown | 4.76 | 192.09 | 77.68 | 170.14 | 0.01 | 0.02 | 2.19 | 1.13 |
| 466 | Positive | Unknown | Unknown | Unknown | 4.72 | 199.06 | 42.52 | 87.68 | 0 | 0.03 | 2.06 | 1.04 |
| 490 | Positive | Unknown | Unknown | Unknown | 4.94 | 204.09 | 653.25 | 1377.17 | 0 | 0.01 | 2.11 | 1.08 |
| 509 | Positive | Unknown | Unknown | Unknown | 5.02 | 211.11 | 46.61 | 81.81 | 0.01 | 0.04 | 1.76 | 0.81 |
| 518 | Positive | Unknown | Unknown | Unknown | 11.31 | 215.14 | 70.94 | 107.73 | 0.01 | 0.05 | 1.52 | 0.6 |
| 535 | Positive | Unknown | Unknown | Unknown | 5.02 | 221.11 | 66.25 | 110.46 | 0.01 | 0.03 | 1.67 | 0.74 |
| 538 | Positive | Unknown | Unknown | N-Acetylgalactosamine | 4.99 | 222.1 | 3804.84 | 8875.24 | 0 | 0 | 2.33 | 1.22 |
| 587 | Positive | Unknown | Unknown | Unknown | 4.84 | 235.09 | 69.1 | 116.87 | 0.01 | 0.03 | 1.69 | 0.76 |
| 597 | Positive | Unknown | Unknown | Unknown | 3.45 | 238.56 | 15.55 | 44.66 | 0 | 0.03 | 2.87 | 1.52 |
| 613 | Positive | Unknown | Unknown | Unknown | 6.03 | 244.08 | 66.88 | 134.31 | 0.02 | 0.03 | 2.01 | 1.01 |
| 639 | Positive | Unknown | Unknown | Unknown | 4.88 | 249.11 | 127.11 | 214.5 | 0 | 0.02 | 1.69 | 0.75 |
| 698 | Positive | Unknown | Unknown | Unknown | 11.48 | 267.1 | 41.02 | 75.46 | 0 | 0 | 1.84 | 0.88 |
| 707 | Positive | Unknown | Unknown | Unknown | 5.02 | 269.11 | 1259.2 | 2216.33 | 0.01 | 0.04 | 1.76 | 0.82 |
| 713 | Positive | Unknown | Unknown | Unknown | 10.93 | 270.13 | 572.2 | 1065.14 | 0 | 0 | 1.86 | 0.9 |
| 718 | Positive | Unknown | Unknown | Unknown | 5.14 | 272.07 | 74.24 | 170.69 | 0 | 0.01 | 2.3 | 1.2 |
| 720 | Positive | Unknown | Unknown | Unknown | 5.33 | 272.07 | 74.96 | 156.89 | 0.01 | 0.04 | 2.09 | 1.07 |
| 737 | Positive | Unknown | Unknown | Unknown | 5.03 | 277.14 | 47.59 | 94.01 | 0.01 | 0.03 | 1.98 | 0.98 |
| 759 | Positive | Unknown | Unknown | Unknown | 9.23 | 284.08 | 26.68 | 63.02 | 0 | 0.02 | 2.36 | 1.24 |
| 779 | Positive | Unknown | Unknown | Unknown | 2.92 | 289.03 | 22.89 | 66.64 | 0.02 | 0.05 | 2.91 | 1.54 |
| 799 | Positive | Unknown | Unknown | Unknown | 4.78 | 293.14 | 36.93 | 146.78 | 0 | 0.01 | 3.97 | 1.99 |
| 807 | Positive | Unknown | Unknown | Unknown | 9.4 | 295.1 | 62.11 | 109.32 | 0.01 | 0.03 | 1.76 | 0.82 |
| 817 | Positive | Unknown | Unknown | Unknown | 12.29 | 298.1 | 48.87 | 86.34 | 0 | 0.04 | 1.77 | 0.82 |
| 835 | Positive | Unknown | Unknown | Unknown | 4.74 | 305.13 | 63.59 | 129.55 | 0 | 0.02 | 2.04 | 1.03 |
| 844 | Positive | Unknown | Unknown | Unknown | 11.87 | 307.1 | 145.62 | 258.07 | 0 | 0.01 | 1.77 | 0.83 |
| 864 | Positive | Unknown | Unknown | Unknown | 14.74 | 313.27 | 60.83 | 113.71 | 0.01 | 0.02 | 1.87 | 0.9 |
| 886 | Positive | Unknown | Unknown | Unknown | 5.02 | 323.15 | 22264.91 | 39969.24 | 0.01 | 0.04 | 1.8 | 0.84 |
| 908 | Positive | Unknown | Unknown | Unknown | 5.02 | 332.07 | 80.58 | 175.9 | 0.01 | 0.03 | 2.18 | 1.13 |
| 923 | Positive | Unknown | Unknown | Asn-agam | 4.61 | 336.14 | 7.66 | 13.42 | 0.03 | 0.05 | 1.75 | 0.81 |
| 946 | Positive | Unknown | Unknown | Unknown | 17.81 | 347.08 | 2.63 | 1.3 | 0.01 | 0.04 | 0.5 | -1.01 |
| 947 | Positive | Unknown | Unknown | Unknown | 5.01 | 347.1 | 28.05 | 70.64 | 0.01 | 0.02 | 2.52 | 1.33 |
| 973 | Positive | Unknown | Unknown | Unknown | 5.22 | 353.09 | 30.92 | 65.03 | 0.01 | 0.04 | 2.1 | 1.07 |
| 974 | Positive | Unknown | Unknown | Unknown | 5.5 | 353.09 | 35.21 | 82.76 | 0 | 0.01 | 2.35 | 1.23 |
| 990 | Positive | Unknown | Spectral Match to D-(+)-Trehalose from NIST14 | Unknown | 4.81 | 360.15 | 40.17 | 105.34 | 0 | 0.03 | 2.62 | 1.39 |
| 993 | Positive | Unknown | Unknown | Unknown | 4.92 | 362.1 | 140.06 | 284.41 | 0 | 0.02 | 2.03 | 1.02 |
| 1000 | Positive | Dihexose (Maltose/Sucrose) | Unknown | Unknown | 4.92 | 365.11 | 123.01 | 247.26 | 0 | 0.02 | 2.01 | 1.01 |
| 1026 | Positive | Unknown | Unknown | Unknown | 5.02 | 376.06 | 32.45 | 71.82 | 0.01 | 0.03 | 2.21 | 1.15 |
| 1045 | Positive | Unknown | Unknown | Unknown | 4.91 | 381.08 | 37.34 | 81.12 | 0 | 0.04 | 2.17 | 1.12 |
| 1059 | Positive | Unknown | Unknown | Unknown | 4.92 | 385.09 | 19.29 | 48.46 | 0 | 0.02 | 2.51 | 1.33 |
| 1072 | Positive | Unknown | Unknown | Unknown | 10.91 | 390.1 | 36.84 | 70.32 | 0.03 | 0.05 | 1.91 | 0.93 |
| 1089 | Positive | Unknown | Unknown | Unknown | 11.87 | 397.15 | 183.44 | 288.61 | 0.01 | 0.03 | 1.57 | 0.65 |
| 1111 | Positive | Unknown | Unknown | Unknown | 9.38 | 406.17 | 226.51 | 385.6 | 0.01 | 0.02 | 1.7 | 0.77 |
| 1174 | Positive | Unknown | Unknown | Unknown | 11.87 | 432.19 | 222.83 | 362.44 | 0.01 | 0.03 | 1.63 | 0.7 |
| 1187 | Positive | Unknown | Unknown | Unknown | 11.87 | 437.14 | 217.74 | 389.54 | 0 | 0.01 | 1.79 | 0.84 |
| 1241 | Positive | Unknown | Unknown | Unknown | 11.88 | 453.12 | 57.99 | 97.38 | 0.01 | 0.03 | 1.68 | 0.75 |
| 1255 | Positive | Unknown | Unknown | Unknown | 12.86 | 458.17 | 78.68 | 139.13 | 0 | 0.01 | 1.77 | 0.82 |
| 1280 | Positive | Unknown | Unknown | Unknown | 9.8 | 466.19 | 135.08 | 228.86 | 0.01 | 0.05 | 1.69 | 0.76 |
| 1336 | Positive | Unknown | Unknown | Unknown | 14.68 | 487.27 | 62.81 | 139.42 | 0.01 | 0.02 | 2.22 | 1.15 |
| 1397 | Positive | Unknown | Unknown | Unknown | 12.57 | 509.22 | 185.32 | 286.65 | 0.01 | 0.04 | 1.55 | 0.63 |
| 1409 | Positive | Unknown | Unknown | Unknown | 13.06 | 515.15 | 41.45 | 65.24 | 0 | 0 | 1.57 | 0.65 |
| 1430 | Positive | Unknown | Melezitose | Unknown | 5.14 | 522.2 | 20.53 | 75.18 | 0 | 0 | 3.66 | 1.87 |
| 1436 | Positive | Unknown | Unknown | Unknown | 5.15 | 524.15 | 5.41 | 59.91 | 0 | 0.01 | 11.07 | 3.47 |
| 1445 | Positive | Unknown | Unknown | Unknown | 5.17 | 527.16 | 20.64 | 69.46 | 0 | 0 | 3.36 | 1.75 |
| 1461 | Positive | Unknown | Unknown | Unknown | 4.92 | 533.16 | 53.45 | 197.84 | 0 | 0.03 | 3.7 | 1.89 |
| 1578 | Positive | Unknown | Unknown | Unknown | 4.43 | 577.18 | 26.55 | 53.57 | 0.02 | 0.03 | 2.02 | 1.01 |
| 1776 | Positive | Unknown | Unknown | Unknown | 13.19 | 663.19 | 152.74 | 244.75 | 0 | 0.01 | 1.6 | 0.68 |
| 1807 | Positive | Unknown | Unknown | Unknown | 13.28 | 677.21 | 845.27 | 1365.83 | 0 | 0.01 | 1.62 | 0.69 |
| 1808 | Positive | Unknown | Unknown | Unknown | 13.57 | 677.21 | 150.68 | 248.09 | 0 | 0.01 | 1.65 | 0.72 |
| 1834 | Positive | Unknown | Unknown | Unknown | 13.06 | 689.21 | 139.98 | 215.93 | 0.01 | 0.02 | 1.54 | 0.63 |
| 1879 | Positive | Unknown | Unknown | Unknown | 4.92 | 704.21 | 10.77 | 68.17 | 0.01 | 0.03 | 6.33 | 2.66 |
| 1934 | Positive | Unknown | Unknown | Unknown | 12.88 | 726.26 | 44.01 | 76 | 0.01 | 0.04 | 1.73 | 0.79 |
| 1995 | Positive | Unknown | Unknown | Unknown | 11.45 | 751.19 | 16.74 | 35.69 | 0.02 | 0.05 | 2.13 | 1.09 |
| 2033 | Positive | Unknown | Unknown | Unknown | 14.76 | 772.59 | 31.28 | 69.8 | 0.01 | 0.03 | 2.23 | 1.16 |
| 2045 | Positive | Unknown | Spectral Match to 1,2-Dilinoleoyl-sn-glycero-3-phosphocholine from NIST14 | Unknown | 15.21 | 782.57 | 130.59 | 320.48 | 0.02 | 0.04 | 2.45 | 1.3 |
| 2138 | Positive | Unknown | Unknown | Unknown | 14.38 | 916.51 | 45.4 | 83.98 | 0.01 | 0.02 | 1.85 | 0.89 |
| 2152 | Positive | Unknown | Unknown | Unknown | 14.68 | 934.65 | 35.41 | 86.5 | 0.01 | 0.04 | 2.44 | 1.29 |
| 2165 | Positive | Unknown | Unknown | Unknown | 13.34 | 962.33 | 117.44 | 191.73 | 0.01 | 0.02 | 1.63 | 0.71 |
| 2166 | Positive | Unknown | Unknown | Unknown | 14.65 | 963.6 | 17.34 | 46.5 | 0.01 | 0.04 | 2.68 | 1.42 |

## Table S6: Differentially accumulated compounds in the A compartment of black oat grown with intraspecific neighbors (BO-BO/A) compared to black oat grown alone (BO-0/A) in the black grass experimental set (G). Data are from the second 24-hour methanolic re-exudation extract. Compounds were identified using multiple confidence levels according to the Schymanski scale: Level 1 identification was based on matching to authentic standards (annotation "w/o MS2" indicates no confident MS2 spectral match); Level 2a identification was obtained from spectral library matching via MS-FINDER and GNPS; Level 2b identification was predicted using MS-FINDER and SIRIUS CSI:FingerID. Statistical significance was determined by Welch's t-test (FDR-corrected p < 0.05) and |log2 fold change| > 0.6.

| Alignment ID | Ionization Mode | Level 1 Identification | Level 2a Identification | Level 2b Identification | Average Rt(min) | Average Mz | BO-0/A | BO-BO/A | p.value | fdr adjusted p.value | Fold Change | Log2 fold change |
| --- | --- | --- | --- | --- | --- | --- | --- | --- | --- | --- | --- | --- |
| 183 | Negative | w/o MS2:N-acetyl-D-mannosamine | Unknown | N-Acetylgalactosamine | 4.91 | 220.08 | 223.99 | 375.82 | 0.01 | 0.02 | 1.68 | 0.75 |
| 246 | Negative | Unknown | Unknown | Unknown | 9.05 | 252.02 | 405.65 | 687.86 | 0.02 | 0.05 | 1.7 | 0.76 |
| 254 | Negative | Unknown | Unknown | Unknown | 3.06 | 257.02 | 16.11 | 34.54 | 0.02 | 0.03 | 2.14 | 1.1 |
| 263 | Negative | Unknown | Unknown | Unknown | 2.94 | 261 | 328.54 | 623.99 | 0.01 | 0.03 | 1.9 | 0.93 |
| 276 | Negative | Unknown | Unknown | Unknown | 9.1 | 267.02 | 253.95 | 436.81 | 0.01 | 0.04 | 1.72 | 0.78 |
| 374 | Negative | Unknown | Unknown | Unknown | 8.92 | 311.07 | 63.11 | 140.61 | 0 | 0.01 | 2.23 | 1.16 |
| 398 | Negative | Unknown | Unknown | Unknown | 12.62 | 321.1 | 247.44 | 461.58 | 0.01 | 0.02 | 1.87 | 0.9 |
| 464 | Negative | Unknown | Unknown | Unknown | 10.12 | 351.13 | 1980.91 | 3178.27 | 0.01 | 0.04 | 1.6 | 0.68 |
| 473 | Negative | Unknown | Unknown | Unknown | 9.96 | 355.05 | 33.69 | 67.54 | 0 | 0.01 | 2 | 1 |
| 476 | Negative | Unknown | Unknown | Unknown | 5.17 | 355.09 | 74.91 | 126.66 | 0.01 | 0.02 | 1.69 | 0.76 |
| 519 | Negative | Unknown | Unknown | Unknown | 9.11 | 373.08 | 2062.29 | 3317.03 | 0.02 | 0.04 | 1.61 | 0.69 |
| 542 | Negative | Unknown | Unknown | Unknown | 9.95 | 384.99 | 29.36 | 48.48 | 0.01 | 0.02 | 1.65 | 0.72 |
| 545 | Negative | Unknown | Unknown | Unknown | 10.04 | 385.11 | 79.96 | 126.43 | 0.01 | 0.02 | 1.58 | 0.66 |
| 546 | Negative | Unknown | Unknown | Unknown | 10.49 | 385.11 | 433.4 | 664.15 | 0.01 | 0.04 | 1.53 | 0.62 |
| 552 | Negative | Unknown | Unknown | Unknown | 11.91 | 387.09 | 50.09 | 86.12 | 0.02 | 0.04 | 1.72 | 0.78 |
| 565 | Negative | Unknown | Unknown | Unknown | 2.93 | 390.96 | 107.06 | 203.19 | 0.01 | 0.03 | 1.9 | 0.92 |
| 592 | Negative | Unknown | Unknown | Unknown | 9.12 | 405.05 | 29.84 | 50.57 | 0.01 | 0.03 | 1.69 | 0.76 |
| 644 | Negative | Unknown | Unknown | Licoagroside B | 9.88 | 431.12 | 11.79 | 18.82 | 0.02 | 0.03 | 1.6 | 0.68 |
| 645 | Negative | Unknown | Unknown | Unknown | 9.48 | 431.12 | 205.27 | 383.41 | 0 | 0.02 | 1.87 | 0.9 |
| 650 | Negative | Unknown | Unknown | Unknown | 9.47 | 433.14 | 758.22 | 1204.67 | 0.01 | 0.02 | 1.59 | 0.67 |
| 667 | Negative | Unknown | Unknown | Unknown | 10.75 | 442.09 | 199.92 | 321.23 | 0.01 | 0.02 | 1.61 | 0.68 |
| 670 | Negative | Unknown | Unknown | Unknown | 10.75 | 444.11 | 82.42 | 132 | 0.01 | 0.03 | 1.6 | 0.68 |
| 682 | Negative | Unknown | Unknown | Unknown | 10.12 | 451.05 | 186.98 | 334.03 | 0.01 | 0.03 | 1.79 | 0.84 |
| 713 | Negative | Unknown | Unknown | Unknown | 9.35 | 461.13 | 10.57 | 16.11 | 0.03 | 0.05 | 1.52 | 0.61 |
| 716 | Negative | Unknown | Unknown | Unknown | 10.66 | 461.17 | 278.15 | 438.39 | 0.01 | 0.02 | 1.58 | 0.66 |
| 720 | Negative | Unknown | Unknown | Unknown | 9.62 | 463.14 | 149.4 | 269.25 | 0 | 0.01 | 1.8 | 0.85 |
| 739 | Negative | Unknown | Unknown | Unknown | 10.37 | 472.1 | 33.23 | 66.73 | 0.01 | 0.03 | 2.01 | 1.01 |
| 762 | Negative | Unknown | Unknown | Unknown | 10.5 | 485.04 | 44.24 | 75.6 | 0.01 | 0.03 | 1.71 | 0.77 |
| 788 | Negative | Unknown | Unknown | PubChem:(117768116) | 12.81 | 498.18 | 55.3 | 93.9 | 0.03 | 0.05 | 1.7 | 0.76 |
| 796 | Negative | Unknown | Unknown | Unknown | 5.07 | 503.16 | 1575.3 | 3175.05 | 0.02 | 0.04 | 2.02 | 1.01 |
| 797 | Negative | Unknown | Unknown | Unknown | 12.14 | 503.18 | 65.5 | 132.89 | 0.01 | 0.02 | 2.03 | 1.02 |
| 808 | Negative | Unknown | Unknown | Unknown | 12.69 | 507.21 | 1056.05 | 1828.34 | 0.01 | 0.02 | 1.73 | 0.79 |
| 814 | Negative | Unknown | Unknown | Unknown | 13.13 | 509.22 | 701.73 | 1091.87 | 0.01 | 0.05 | 1.56 | 0.64 |
| 824 | Negative | Unknown | Unknown | Unknown | 4.76 | 515.13 | 63.18 | 156.72 | 0 | 0.01 | 2.48 | 1.31 |
| 856 | Negative | Unknown | Unknown | Unknown | 9.86 | 533.17 | 162.46 | 280.03 | 0.02 | 0.04 | 1.72 | 0.79 |
| 858 | Negative | Unknown | Unknown | Unknown | 4.97 | 533.17 | 148.61 | 388.62 | 0.01 | 0.05 | 2.62 | 1.39 |
| 862 | Negative | Unknown | Unknown | Unknown | 5.32 | 535.15 | 11.46 | 32 | 0.01 | 0.03 | 2.79 | 1.48 |
| 868 | Negative | Unknown | Unknown | Unknown | 4.84 | 537.17 | 95.41 | 194.5 | 0.03 | 0.04 | 2.04 | 1.03 |
| 895 | Negative | Unknown | Unknown | Unknown | 12.98 | 552.19 | 100.77 | 157.06 | 0.02 | 0.04 | 1.56 | 0.64 |
| 916 | Negative | Unknown | Unknown | Unknown | 9.26 | 573.15 | 115.71 | 214.08 | 0 | 0 | 1.85 | 0.89 |
| 918 | Negative | Unknown | Unknown | Unknown | 11.43 | 575.2 | 39.83 | 61.61 | 0.02 | 0.04 | 1.55 | 0.63 |
| 923 | Negative | Unknown | Unknown | Unknown | 9.64 | 583.2 | 111.18 | 190.77 | 0 | 0.02 | 1.72 | 0.78 |
| 927 | Negative | Unknown | Unknown | Unknown | 12.98 | 589.15 | 73.57 | 125.52 | 0.01 | 0.02 | 1.71 | 0.77 |
| 957 | Negative | Unknown | Unknown | Unknown | 14.7 | 619.42 | 68.44 | 143.1 | 0.01 | 0.04 | 2.09 | 1.06 |
| 980 | Negative | Unknown | Unknown | Unknown | 5.32 | 651.16 | 40.59 | 77.32 | 0.01 | 0.04 | 1.9 | 0.93 |
| 993 | Negative | Unknown | Unknown | Unknown | 8.13 | 668.2 | 20.67 | 41.05 | 0.01 | 0.01 | 1.99 | 0.99 |
| 1012 | Negative | Unknown | Unknown | Unknown | 15.32 | 685.48 | 33.58 | 80.42 | 0.01 | 0.03 | 2.39 | 1.26 |
| 1016 | Negative | Unknown | Unknown | Unknown | 11.39 | 687.19 | 70.65 | 119.58 | 0.01 | 0.02 | 1.69 | 0.76 |
| 1024 | Negative | Unknown | Unknown | PubChem:(91847146) | 5.06 | 695.22 | 32.46 | 108.82 | 0.02 | 0.05 | 3.35 | 1.75 |
| 1064 | Negative | Unknown | Unknown | Unknown | 12.04 | 725.32 | 22.61 | 36.5 | 0.02 | 0.04 | 1.61 | 0.69 |
| 1099 | Negative | Unknown | Unknown | Unknown | 11.52 | 747.16 | 44.4 | 102.19 | 0 | 0.02 | 2.3 | 1.2 |
| 1104 | Negative | Unknown | Unknown | Unknown | 11.52 | 749.17 | 53.16 | 113.53 | 0 | 0.02 | 2.14 | 1.09 |
| 1141 | Negative | Unknown | Unknown | Unknown | 10.98 | 779.19 | 13.77 | 35.26 | 0.01 | 0.03 | 2.56 | 1.36 |
| 1150 | Negative | Unknown | Unknown | Unknown | 13.33 | 787.26 | 32.18 | 52.17 | 0.01 | 0.03 | 1.62 | 0.7 |
| 1163 | Negative | Unknown | Unknown | Unknown | 13.06 | 803.2 | 2.52 | 4.36 | 0 | 0.01 | 1.73 | 0.79 |
| 1169 | Negative | Unknown | Unknown | Unknown | 13.24 | 805.28 | 36.77 | 65.91 | 0.01 | 0.02 | 1.79 | 0.84 |
| 1190 | Negative | Unknown | Unknown | Unknown | 5.06 | 825.3 | 25.81 | 89.21 | 0.01 | 0.04 | 3.46 | 1.79 |
| 1209 | Negative | Unknown | Unknown | Unknown | 12.65 | 845.45 | 58.49 | 105.9 | 0.02 | 0.04 | 1.81 | 0.86 |
| 1305 | Negative | Unknown | Unknown | Unknown | 13.17 | 1109.4 | 84.31 | 144.13 | 0.02 | 0.05 | 1.71 | 0.77 |
| 1328 | Negative | Unknown | Unknown | Unknown | 13.12 | 1375.39 | 36.63 | 70.19 | 0 | 0.01 | 1.92 | 0.94 |
| 96 | Positive | Unknown | Unknown | Unknown | 3.69 | 85.03 | 242.86 | 408.31 | 0.01 | 0.03 | 1.68 | 0.75 |
| 97 | Positive | Unknown | Unknown | Unknown | 3.3 | 85.03 | 160.7 | 313.11 | 0.01 | 0.03 | 1.95 | 0.96 |
| 161 | Positive | Unknown | Choline; LC-ESI-QTOF; MS2; CE | Unknown | 3.57 | 104.11 | 153.19 | 354.16 | 0.02 | 0.04 | 2.31 | 1.21 |
| 213 | Positive | Unknown | Unknown | Unknown | 4.86 | 120.07 | 29.12 | 55.05 | 0 | 0.01 | 1.89 | 0.92 |
| 220 | Positive | Unknown | Unknown | Unknown | 6.7 | 123.04 | 175.8 | 294.66 | 0.02 | 0.05 | 1.68 | 0.75 |
| 230 | Positive | Unknown | Unknown | Unknown | 9.6 | 127.04 | 26.28 | 42.08 | 0.01 | 0.03 | 1.6 | 0.68 |
| 269 | Positive | Unknown | Unknown | Unknown | 6.72 | 136.08 | 378.75 | 660.71 | 0.01 | 0.03 | 1.74 | 0.8 |
| 301 | Positive | Unknown | Unknown | Unknown | 3.7 | 145.05 | 77.72 | 158.15 | 0.01 | 0.02 | 2.03 | 1.02 |
| 306 | Positive | Unknown | Unknown | Unknown | 5.16 | 146.09 | 62.85 | 137.74 | 0.01 | 0.03 | 2.19 | 1.13 |
| 311 | Positive | Glutamine | L-Glutamine; LC-ESI-QTOF; MS2; CE | L-Glutamine | 3.22 | 147.08 | 508.91 | 1151.84 | 0 | 0.01 | 2.26 | 1.18 |
| 326 | Positive | Unknown | Unknown | Unknown | 10.62 | 152.07 | 411.53 | 722.78 | 0.02 | 0.03 | 1.76 | 0.81 |
| 330 | Positive | Unknown | Unknown | Unknown | 5.43 | 154.02 | 64.09 | 104.72 | 0.01 | 0.04 | 1.63 | 0.71 |
| 335 | Positive | Unknown | Unknown | Unknown | 9.89 | 155.07 | 33.2 | 62.01 | 0.01 | 0.03 | 1.87 | 0.9 |
| 363 | Positive | Unknown | Unknown | Unknown | 10.72 | 162.05 | 30.26 | 74.14 | 0.02 | 0.04 | 2.45 | 1.29 |
| 451 | Positive | Unknown | Unknown | Unknown | 3.63 | 185.04 | 46.21 | 87.65 | 0.02 | 0.04 | 1.9 | 0.92 |
| 454 | Positive | Unknown | Unknown | Unknown | 5.01 | 186.08 | 210.18 | 436.29 | 0 | 0.01 | 2.08 | 1.05 |
| 465 | Positive | Unknown | Unknown | Unknown | 3.7 | 191.04 | 765.07 | 1393.5 | 0.02 | 0.05 | 1.82 | 0.87 |
| 469 | Positive | Unknown | Unknown | Unknown | 4.74 | 192.09 | 84 | 143.15 | 0.01 | 0.01 | 1.7 | 0.77 |
| 492 | Positive | Unknown | Unknown | Unknown | 3.63 | 200.04 | 2863.28 | 5986.76 | 0.02 | 0.05 | 2.09 | 1.06 |
| 509 | Positive | Unknown | Unknown | Unknown | 4.96 | 204.09 | 346.96 | 686.27 | 0.01 | 0.03 | 1.98 | 0.98 |
| 559 | Positive | Unknown | Unknown | N-Acetylgalactosamine | 4.99 | 222.1 | 2845.58 | 5754.06 | 0 | 0.01 | 2.02 | 1.02 |
| 577 | Positive | Unknown | Unknown | Unknown | 6.05 | 226.07 | 19.15 | 44.09 | 0 | 0.01 | 2.3 | 1.2 |
| 578 | Positive | Unknown | Unknown | Unknown | 3.84 | 226.11 | 553.34 | 1688.08 | 0 | 0.01 | 3.05 | 1.61 |
| 662 | Positive | Unknown | Unknown | Unknown | 4.85 | 249.11 | 102.03 | 197.05 | 0.02 | 0.05 | 1.93 | 0.95 |
| 673 | Positive | Unknown | Unknown | Unknown | 3.45 | 252.14 | 40.03 | 104.74 | 0 | 0.01 | 2.62 | 1.39 |
| 711 | Positive | Unknown | Unknown | Unknown | 4.88 | 262.09 | 58.74 | 128.87 | 0.01 | 0.03 | 2.19 | 1.13 |
| 723 | Positive | Unknown | Unknown | Unknown | 9.56 | 265.07 | 76.29 | 136.48 | 0.01 | 0.02 | 1.79 | 0.84 |
| 770 | Positive | Unknown | Unknown | Unknown | 5.04 | 277.14 | 48.53 | 114.33 | 0.01 | 0.04 | 2.36 | 1.24 |
| 784 | Positive | Unknown | Unknown | Unknown | 3.76 | 281.07 | 93.53 | 222.23 | 0.02 | 0.05 | 2.38 | 1.25 |
| 785 | Positive | Unknown | Unknown | Unknown | 4.88 | 281.07 | 29.48 | 56.69 | 0.02 | 0.04 | 1.92 | 0.94 |
| 800 | Positive | Unknown | Unknown | Unknown | 9.33 | 286.09 | 51.68 | 106.12 | 0.01 | 0.04 | 2.05 | 1.04 |
| 809 | Positive | Unknown | Unknown | Unknown | 9.16 | 288.09 | 640.51 | 1256.53 | 0.01 | 0.05 | 1.96 | 0.97 |
| 813 | Positive | Unknown | 2-methyl-3-[(2S,3R,4S,5S,6R)-3,4,5-trihydroxy-6-(hydroxymethyl)oxan-2-yl]oxypyran-4-one | maltol beta-D-glucopyranoside | 9.13 | 289.09 | 122.98 | 231.93 | 0.02 | 0.03 | 1.89 | 0.92 |
| 814 | Positive | Unknown | Unknown | Unknown | 4.38 | 289.09 | 272.59 | 598.87 | 0.01 | 0.01 | 2.2 | 1.14 |
| 836 | Positive | Unknown | Unknown | Unknown | 3.68 | 295.01 | 52.35 | 101.92 | 0.02 | 0.04 | 1.95 | 0.96 |
| 860 | Positive | Unknown | Unknown | Unknown | 11.44 | 301.56 | 29.29 | 68.44 | 0.01 | 0.04 | 2.34 | 1.22 |
| 874 | Positive | Unknown | Unknown | Unknown | 12.08 | 307.1 | 265.37 | 416.25 | 0.02 | 0.05 | 1.57 | 0.65 |
| 875 | Positive | Unknown | Unknown | Unknown | 12.96 | 307.1 | 202.74 | 334.27 | 0.01 | 0.04 | 1.65 | 0.72 |
| 888 | Positive | Unknown | Unknown | Unknown | 11.44 | 311.58 | 33.42 | 82.81 | 0 | 0.03 | 2.48 | 1.31 |
| 897 | Positive | Unknown | Unknown | Unknown | 10.16 | 314.12 | 46.67 | 80.02 | 0.02 | 0.04 | 1.71 | 0.78 |
| 910 | Positive | Unknown | Unknown | Unknown | 15.38 | 319.19 | 92.28 | 276.54 | 0.01 | 0.02 | 3 | 1.58 |
| 923 | Positive | Unknown | Unknown | Unknown | 5 | 323.22 | 365.65 | 3602.45 | 0 | 0.02 | 9.85 | 3.3 |
| 1025 | Positive | Unknown | Unknown | Unknown | 4.88 | 362.1 | 99.6 | 207.93 | 0.02 | 0.05 | 2.09 | 1.06 |
| 1062 | Positive | Unknown | Unknown | Unknown | 11.45 | 375.09 | 126.83 | 314.77 | 0.01 | 0.03 | 2.48 | 1.31 |
| 1065 | Positive | Unknown | Unknown | Unknown | 11.45 | 376.1 | 137.93 | 337.05 | 0.01 | 0.04 | 2.44 | 1.29 |
| 1072 | Positive | Unknown | Unknown | Unknown | 15.33 | 377.74 | 74.9 | 182.36 | 0.01 | 0.04 | 2.43 | 1.28 |
| 1102 | Positive | Unknown | Unknown | Unknown | 10.94 | 390.1 | 37.89 | 110.33 | 0.01 | 0.03 | 2.91 | 1.54 |
| 1120 | Positive | Unknown | Unknown | Unknown | 15.34 | 397.76 | 27.24 | 77.44 | 0.01 | 0.02 | 2.84 | 1.51 |
| 1123 | Positive | Unknown | Unknown | Unknown | 15.39 | 398.76 | 179.2 | 462.86 | 0.02 | 0.05 | 2.58 | 1.37 |
| 1139 | Positive | Unknown | Unknown | Unknown | 10.47 | 404.15 | 142.25 | 224.59 | 0.02 | 0.05 | 1.58 | 0.66 |
| 1148 | Positive | Unknown | Unknown | Unknown | 15.35 | 410.76 | 54.15 | 148.65 | 0.01 | 0.02 | 2.75 | 1.46 |
| 1170 | Positive | Unknown | Unknown | Unknown | 9.95 | 420.19 | 23.74 | 37.26 | 0.01 | 0.03 | 1.57 | 0.65 |
| 1173 | Positive | Unknown | Unknown | Unknown | 9.14 | 422.11 | 231.64 | 441.61 | 0.01 | 0.05 | 1.91 | 0.93 |
| 1186 | Positive | Unknown | Unknown | Unknown | 5.43 | 427.07 | 29.91 | 49.02 | 0.02 | 0.05 | 1.64 | 0.71 |
| 1202 | Positive | Unknown | Spectral Match to Isovitexin from NIST14 | Unknown | 12.71 | 433.11 | 26.55 | 43.95 | 0.02 | 0.05 | 1.66 | 0.73 |
| 1203 | Positive | Unknown | Unknown | Unknown | 9.89 | 433.13 | 78.91 | 142.49 | 0.01 | 0.01 | 1.81 | 0.85 |
| 1205 | Positive | Unknown | Unknown | Unknown | 10.69 | 434.2 | 194 | 296.91 | 0.01 | 0.04 | 1.53 | 0.61 |
| 1206 | Positive | Unknown | Unknown | Unknown | 9.64 | 436.18 | 67.64 | 135.83 | 0 | 0.01 | 2.01 | 1.01 |
| 1226 | Positive | Unknown | Unknown | Unknown | 10.73 | 444.11 | 409.1 | 691.99 | 0.01 | 0.02 | 1.69 | 0.76 |
| 1235 | Positive | Unknown | Unknown | Unknown | 9.14 | 446.12 | 64.64 | 128.29 | 0.01 | 0.03 | 1.98 | 0.99 |
| 1293 | Positive | Unknown | Unknown | Unknown | 9.89 | 466.19 | 147.91 | 277.34 | 0.01 | 0.02 | 1.88 | 0.91 |
| 1314 | Positive | Unknown | Unknown | Unknown | 10.36 | 474.12 | 54.45 | 129.54 | 0 | 0.01 | 2.38 | 1.25 |
| 1322 | Positive | Unknown | Unknown | Unknown | 12.17 | 476.21 | 40.25 | 88.75 | 0 | 0.01 | 2.21 | 1.14 |
| 1327 | Positive | Unknown | Unknown | Unknown | 15.36 | 478.33 | 168.81 | 418.99 | 0.01 | 0.04 | 2.48 | 1.31 |
| 1337 | Positive | Unknown | Unknown | Unknown | 12.16 | 481.17 | 32.36 | 68.6 | 0.01 | 0.02 | 2.12 | 1.08 |
| 1350 | Positive | Unknown | Unknown | Unknown | 14.73 | 487.27 | 42.88 | 92.08 | 0 | 0.02 | 2.15 | 1.1 |
| 1365 | Positive | Unknown | Unknown | Unknown | 12.7 | 491.21 | 120.61 | 209.03 | 0.01 | 0.03 | 1.73 | 0.79 |
| 1390 | Positive | Unknown | Unknown | Unknown | 15.3 | 502.33 | 105.17 | 242.22 | 0.02 | 0.05 | 2.3 | 1.2 |
| 1455 | Positive | Unknown | Unknown | Unknown | 12.69 | 531.2 | 141.44 | 216.82 | 0.03 | 0.04 | 1.53 | 0.62 |
| 1762 | Positive | Unknown | Unknown | Unknown | 13.28 | 663.19 | 124.81 | 211.79 | 0.01 | 0.04 | 1.7 | 0.76 |
| 1773 | Positive | Unknown | Unknown | Unknown | 8.14 | 670.22 | 44.34 | 109.23 | 0 | 0.01 | 2.46 | 1.3 |
| 1787 | Positive | Unknown | Unknown | Unknown | 15.19 | 676.49 | 115.63 | 240.15 | 0.02 | 0.04 | 2.08 | 1.05 |
| 1788 | Positive | Unknown | Unknown | Unknown | 13.36 | 677.21 | 733.18 | 1165.72 | 0.01 | 0.04 | 1.59 | 0.67 |
| 1815 | Positive | Unknown | Unknown | Unknown | 13.14 | 689.21 | 133.71 | 209.94 | 0.01 | 0.03 | 1.57 | 0.65 |
| 1869 | Positive | Unknown | Unknown | Unknown | 12.7 | 716.28 | 80.54 | 160.09 | 0.01 | 0.03 | 1.99 | 0.99 |
| 1894 | Positive | Unknown | Unknown | Unknown | 12.95 | 726.26 | 43.26 | 82.55 | 0 | 0.02 | 1.91 | 0.93 |
| 1920 | Positive | Unknown | Unknown | Unknown | 15.15 | 738.51 | 72.55 | 176.69 | 0.02 | 0.04 | 2.44 | 1.28 |
| 1951 | Positive | Unknown | Unknown | Unknown | 15.28 | 756.55 | 102.85 | 260.93 | 0.02 | 0.04 | 2.54 | 1.34 |
| 1976 | Positive | Unknown | Unknown | Unknown | 14.82 | 772.59 | 30.04 | 63.64 | 0.02 | 0.05 | 2.12 | 1.08 |
| 2021 | Positive | Unknown | Unknown | Unknown | 14.73 | 814.64 | 25.47 | 52.18 | 0.01 | 0.02 | 2.05 | 1.03 |
| 2087 | Positive | Unknown | Unknown | Unknown | 14.73 | 934.65 | 34.07 | 72.07 | 0.01 | 0.03 | 2.12 | 1.08 |
| 2097 | Positive | Unknown | Unknown | Unknown | 14.77 | 955.58 | 19.99 | 50.19 | 0.02 | 0.05 | 2.51 | 1.33 |

## Table S7: Differentially accumulated compounds in the A compartment of black oat grown with redroot pigweed as interspecific neighbor (BO-P/A) compared to black oat grown alone (BO-0/A) in the redroot pigweed experimental set (P). Data are from the second 24-hour methanolic re-exudation extract. Compounds were identified using multiple confidence levels according to the Schymanski scale: Level 1 identification was based on matching to authentic standards (annotation "w/o MS2" indicates no confident MS2 spectral match); Level 2a identification was obtained from spectral library matching via MS-FINDER and GNPS; Level 2b identification was predicted using MS-FINDER and SIRIUS CSI:FingerID. Statistical significance was determined by Welch's t-test (FDR-corrected p < 0.05) and |log2 fold change| > 0.6.

| Alignment ID | Ionization Mode | Level 1 Identification | Level 2a Identification | Level 2b Identification | Average Rt(min) | Average Mz | BO-0/A | BO-P/A | p.value | fdr adjusted p.value | Fold Change | Log2 fold change |
| --- | --- | --- | --- | --- | --- | --- | --- | --- | --- | --- | --- | --- |
| 2 | Negative | Unknown | Unknown | Unknown | 11.89 | 57.03 | 58.06 | 121.54 | 0.01 | 0.02 | 2.09 | 1.07 |
| 4 | Negative | Unknown | Unknown | Unknown | 11.89 | 59.01 | 160.23 | 310.51 | 0.01 | 0.04 | 1.94 | 0.95 |
| 69 | Negative | Unknown | Unknown | Unknown | 11.89 | 99.04 | 146.18 | 352.85 | 0 | 0.01 | 2.41 | 1.27 |
| 70 | Negative | Unknown | Unknown | Unknown | 3.74 | 99.04 | 5.33 | 33.93 | 0.03 | 0.05 | 6.36 | 2.67 |
| 129 | Negative | Unknown | Unknown | Unknown | 12.46 | 121 | 100.04 | 176.63 | 0.02 | 0.03 | 1.77 | 0.82 |
| 136 | Negative | Unknown | Unknown | Unknown | 11.89 | 125.02 | 75.38 | 154.32 | 0.01 | 0.03 | 2.05 | 1.03 |
| 229 | Negative | Unknown | Unknown | Unknown | 3.98 | 165.04 | 24.63 | 43.47 | 0.03 | 0.05 | 1.76 | 0.82 |
| 335 | Negative | Unknown | Unknown | Unknown | 4.7 | 195.05 | 102.23 | 220.16 | 0.03 | 0.04 | 2.15 | 1.11 |
| 392 | Negative | Unknown | Unknown | Unknown | 13.52 | 215.03 | 107.13 | 193.72 | 0.02 | 0.04 | 1.81 | 0.85 |
| 414 | Negative | Unknown | Unknown | Unknown | 5.09 | 220.08 | 395.67 | 1088.52 | 0 | 0.01 | 2.75 | 1.46 |
| 425 | Negative | Unknown | Unknown | Unknown | 2.9 | 224.09 | 45.35 | 146.89 | 0 | 0.01 | 3.24 | 1.7 |
| 508 | Negative | Unknown | Unknown | Unknown | 4.99 | 253.09 | 72.82 | 211.17 | 0 | 0.01 | 2.9 | 1.54 |
| 528 | Negative | Unknown | Unknown | Unknown | 4.53 | 260.02 | 75.19 | 158.25 | 0.03 | 0.05 | 2.1 | 1.07 |
| 682 | Negative | Unknown | Unknown | Unknown | 5.17 | 313.11 | 33.77 | 68.59 | 0 | 0.01 | 2.03 | 1.02 |
| 704 | Negative | Unknown | Unknown | Unknown | 12.47 | 321.1 | 514.21 | 985.1 | 0.02 | 0.03 | 1.92 | 0.94 |
| 741 | Negative | Unknown | Unknown | Unknown | 4.68 | 334.13 | 11.45 | 24.72 | 0.03 | 0.04 | 2.16 | 1.11 |
| 744 | Negative | Unknown | Unknown | Unknown | 10.87 | 335.14 | 105.81 | 243.47 | 0.02 | 0.04 | 2.3 | 1.2 |
| 843 | Negative | Unknown | Unknown | Unknown | 9.03 | 373.08 | 3535.57 | 6537.33 | 0.01 | 0.02 | 1.85 | 0.89 |
| 888 | Negative | Unknown | Unknown | Unknown | 11.68 | 387.09 | 51.25 | 109.91 | 0.02 | 0.04 | 2.14 | 1.1 |
| 889 | Negative | Unknown | Unknown | Unknown | 9.14 | 387.1 | 588.64 | 1011.53 | 0.02 | 0.05 | 1.72 | 0.78 |
| 917 | Negative | Unknown | Unknown | Unknown | 11.54 | 393.18 | 78.41 | 151.18 | 0.02 | 0.03 | 1.93 | 0.95 |
| 935 | Negative | Unknown | Unknown | PubChem:(46222441) | 8.49 | 403.09 | 144.79 | 307.92 | 0.02 | 0.04 | 2.13 | 1.09 |
| 960 | Negative | Unknown | Unknown | COCONUT:(CNP0159349 CNP0218833);Natural Products:(UNPD202200);PubChem:(38363079 38363084 45360328 44715457 125416071 125416072 125416073 125416074);SuperNatural:(SN00032625 SN00030299 SN00032624 SN00030300 SN00032623 SN00032622);ZINC bio:(ZINC31169353 ZINC31169357 ZINC35454681 ZINC35454685 ZINC35454688 ZINC35454690);Training Set | 11.89 | 413.15 | 4009.66 | 9883.57 | 0.01 | 0.01 | 2.46 | 1.3 |
| 961 | Negative | Unknown | Unknown | COCONUT:(CNP0159349 CNP0218833);Natural Products:(UNPD202200);PubChem:(38363079 38363084 45360328 44715457 125416071 125416072 125416073 125416074);SuperNatural:(SN00032625 SN00030299 SN00032624 SN00030300 SN00032623 SN00032622);ZINC bio:(ZINC31169353 ZINC31169357 ZINC35454681 ZINC35454685 ZINC35454688 ZINC35454690);Training Set | 11.05 | 413.15 | 60.28 | 143.89 | 0.01 | 0.01 | 2.39 | 1.26 |
| 962 | Negative | Unknown | Unknown | Unknown | 10.55 | 413.15 | 35.24 | 68.79 | 0.03 | 0.05 | 1.95 | 0.96 |
| 966 | Negative | Unknown | Unknown | Unknown | 5.2 | 415.14 | 306.35 | 650.84 | 0.01 | 0.04 | 2.12 | 1.09 |
| 972 | Negative | Unknown | Unknown | Unknown | 9.08 | 417.11 | 159.29 | 269.2 | 0.01 | 0.03 | 1.69 | 0.76 |
| 988 | Negative | Unknown | Unknown | Unknown | 9.15 | 421.08 | 84.13 | 166.39 | 0.01 | 0.02 | 1.98 | 0.98 |
| 991 | Negative | Unknown | Unknown | Unknown | 10.05 | 421.16 | 50.93 | 89.64 | 0.02 | 0.04 | 1.76 | 0.82 |
| 1034 | Negative | Unknown | Unknown | Unknown | 12.71 | 435.19 | 937.41 | 1993.15 | 0.01 | 0.02 | 2.13 | 1.09 |
| 1061 | Negative | Unknown | Unknown | Unknown | 9.4 | 447.11 | 17.13 | 39.42 | 0.01 | 0.02 | 2.3 | 1.2 |
| 1070 | Negative | Unknown | Unknown | Unknown | 12.31 | 449.2 | 106.14 | 171.73 | 0.02 | 0.05 | 1.62 | 0.69 |
| 1089 | Negative | Unknown | Unknown | Unknown | 9.47 | 455.12 | 547.17 | 1022.45 | 0.01 | 0.04 | 1.87 | 0.9 |
| 1092 | Negative | Unknown | Unknown | Unknown | 12.86 | 456.15 | 83.74 | 139.17 | 0.01 | 0.04 | 1.66 | 0.73 |
| 1123 | Negative | Unknown | Unknown | Unknown | 9.11 | 467.05 | 30.3 | 64.1 | 0.01 | 0.03 | 2.12 | 1.08 |
| 1125 | Negative | Unknown | Unknown | Unknown | 12.77 | 467.21 | 416.56 | 812.94 | 0.01 | 0.01 | 1.95 | 0.96 |
| 1149 | Negative | Unknown | Unknown | Unknown | 10.87 | 479.05 | 27.3 | 73.18 | 0.02 | 0.05 | 2.68 | 1.42 |
| 1214 | Negative | Unknown | Unknown | Unknown | 12.58 | 507.21 | 1545.3 | 2691.83 | 0.02 | 0.03 | 1.74 | 0.8 |
| 1230 | Negative | Unknown | Unknown | Unknown | 4.96 | 515.12 | 25.1 | 71.12 | 0.02 | 0.05 | 2.83 | 1.5 |
| 1242 | Negative | Unknown | Unknown | Unknown | 13.28 | 519.24 | 52.95 | 103.77 | 0.01 | 0.04 | 1.96 | 0.97 |
| 1255 | Negative | Unknown | Unknown | Unknown | 13.66 | 529.26 | 317.57 | 111.68 | 0.02 | 0.04 | 0.35 | -1.51 |
| 1258 | Negative | Unknown | Unknown | Unknown | 10.36 | 533.16 | 46.32 | 77.9 | 0.02 | 0.04 | 1.68 | 0.75 |
| 1259 | Negative | Unknown | Unknown | Unknown | 9.76 | 533.17 | 261.98 | 497.17 | 0.01 | 0.03 | 1.9 | 0.92 |
| 1311 | Negative | Unknown | Unknown | Unknown | 5.1 | 562.2 | 41.51 | 131.41 | 0.01 | 0.03 | 3.17 | 1.66 |
| 1329 | Negative | Unknown | Unknown | Unknown | 11.28 | 575.2 | 58.16 | 138.13 | 0.01 | 0.03 | 2.38 | 1.25 |
| 1339 | Negative | Unknown | Unknown | Unknown | 10.63 | 589.18 | 43.81 | 93.32 | 0.01 | 0.03 | 2.13 | 1.09 |
| 1410 | Negative | Unknown | Unknown | Unknown | 14.72 | 653.51 | 15.89 | 31.36 | 0.02 | 0.05 | 1.97 | 0.98 |
| 1415 | Negative | Unknown | Unknown | Unknown | 12.63 | 661.18 | 54.26 | 115.99 | 0 | 0.01 | 2.14 | 1.1 |
| 1428 | Negative | Unknown | Unknown | Unknown | 5.66 | 665.21 | 506.86 | 1049.35 | 0.01 | 0.02 | 2.07 | 1.05 |
| 1467 | Negative | Unknown | Unknown | Unknown | 13.05 | 687.19 | 1336.43 | 2084.86 | 0.02 | 0.04 | 1.56 | 0.64 |
| 1486 | Negative | Unknown | Unknown | Unknown | 12.6 | 697.24 | 108.02 | 300.95 | 0 | 0.01 | 2.79 | 1.48 |
| 1507 | Negative | Unknown | Unknown | Unknown | 11.88 | 711.3 | 25.52 | 66.18 | 0.01 | 0.01 | 2.59 | 1.38 |
| 1547 | Negative | Unknown | Unknown | Unknown | 13.27 | 738.19 | 87.08 | 135.25 | 0.03 | 0.05 | 1.55 | 0.64 |
| 1571 | Negative | Unknown | Unknown | Unknown | 11.7 | 749.17 | 51.35 | 119.39 | 0.02 | 0.03 | 2.32 | 1.22 |
| 1610 | Negative | Unknown | Unknown | Unknown | 5.09 | 781.2 | 60.68 | 194.28 | 0 | 0.05 | 3.2 | 1.68 |
| 1635 | Negative | Unknown | Unknown | Unknown | 11.08 | 808.21 | 67.59 | 158.4 | 0.02 | 0.03 | 2.34 | 1.23 |
| 1636 | Negative | Unknown | Unknown | Unknown | 12.57 | 808.21 | 44.65 | 98.7 | 0.01 | 0.03 | 2.21 | 1.14 |
| 1691 | Negative | Unknown | Unknown | Unknown | 12.58 | 845.45 | 54.65 | 97.66 | 0.02 | 0.03 | 1.79 | 0.84 |
| 1697 | Negative | Unknown | Unknown | Unknown | 12.64 | 857.25 | 83.52 | 133.85 | 0.02 | 0.05 | 1.6 | 0.68 |
| 1730 | Negative | Unknown | Unknown | Unknown | 13.33 | 943.29 | 226.06 | 450.79 | 0 | 0.02 | 1.99 | 1 |
| 1808 | Negative | Unknown | Unknown | Unknown | 13.25 | 1105.34 | 58.88 | 94.5 | 0.01 | 0.04 | 1.61 | 0.68 |
| 1809 | Negative | Unknown | Unknown | Unknown | 13.09 | 1109.41 | 64.05 | 119.66 | 0.01 | 0.02 | 1.87 | 0.9 |
| 1825 | Negative | Unknown | Unknown | Unknown | 13.04 | 1375.39 | 37.25 | 73.63 | 0.01 | 0.01 | 1.98 | 0.98 |
| 35 | Positive | Unknown | Unknown | Unknown | 2.96 | 66.99 | 29.23 | 51.07 | 0 | 0.02 | 1.75 | 0.81 |
| 216 | Positive | Unknown | Unknown | Unknown | 4.88 | 120.07 | 32.56 | 72.71 | 0.01 | 0.04 | 2.23 | 1.16 |
| 274 | Positive | Unknown | Unknown | 2-Aminobenzoic acid | 4.97 | 138.05 | 650.74 | 1509.55 | 0.01 | 0.02 | 2.32 | 1.21 |
| 386 | Positive | Unknown | Unknown | Unknown | 4.99 | 176.09 | 120.45 | 203.22 | 0 | 0 | 1.69 | 0.75 |
| 387 | Positive | Unknown | Unknown | Unknown | 3.72 | 176.09 | 102.02 | 199.12 | 0.01 | 0.04 | 1.95 | 0.96 |
| 422 | Positive | Unknown | Unknown | Unknown | 3.54 | 185.04 | 26.28 | 62.02 | 0.01 | 0.03 | 2.36 | 1.24 |
| 445 | Positive | Unknown | Unknown | Unknown | 4.76 | 192.09 | 77.68 | 157.42 | 0.02 | 0.04 | 2.03 | 1.02 |
| 490 | Positive | Unknown | Unknown | Unknown | 4.94 | 204.09 | 653.25 | 1400.84 | 0 | 0.01 | 2.14 | 1.1 |
| 538 | Positive | Unknown | Unknown | N-Acetylgalactosamine | 4.99 | 222.1 | 3804.84 | 7088.53 | 0 | 0 | 1.86 | 0.9 |
| 613 | Positive | Unknown | Unknown | Unknown | 6.03 | 244.08 | 66.88 | 148.61 | 0 | 0.01 | 2.22 | 1.15 |
| 625 | Positive | Unknown | Unknown | Unknown | 5.07 | 246.09 | 155.2 | 409.9 | 0 | 0.02 | 2.64 | 1.4 |
| 639 | Positive | Unknown | Unknown | Unknown | 4.88 | 249.11 | 127.11 | 288.35 | 0 | 0.01 | 2.27 | 1.18 |
| 713 | Positive | Unknown | Unknown | Unknown | 10.93 | 270.13 | 572.2 | 993.35 | 0 | 0.01 | 1.74 | 0.8 |
| 718 | Positive | Unknown | Unknown | Unknown | 5.14 | 272.07 | 74.24 | 185.26 | 0.01 | 0.02 | 2.5 | 1.32 |
| 720 | Positive | Unknown | Unknown | Unknown | 5.33 | 272.07 | 74.96 | 187.19 | 0 | 0.01 | 2.5 | 1.32 |
| 799 | Positive | Unknown | Unknown | Unknown | 4.78 | 293.14 | 36.93 | 89.92 | 0.02 | 0.04 | 2.44 | 1.28 |
| 807 | Positive | Unknown | Unknown | Unknown | 9.4 | 295.1 | 62.11 | 114.03 | 0.02 | 0.04 | 1.84 | 0.88 |
| 946 | Positive | Unknown | Unknown | Unknown | 17.81 | 347.08 | 2.63 | 1.11 | 0.01 | 0.03 | 0.42 | -1.24 |
| 973 | Positive | Unknown | Unknown | Unknown | 5.22 | 353.09 | 30.92 | 84.58 | 0.01 | 0.03 | 2.74 | 1.45 |
| 974 | Positive | Unknown | Unknown | Unknown | 5.5 | 353.09 | 35.21 | 102.44 | 0.01 | 0.02 | 2.91 | 1.54 |
| 993 | Positive | Unknown | Unknown | Unknown | 4.92 | 362.1 | 140.06 | 310.92 | 0.01 | 0.04 | 2.22 | 1.15 |
| 1000 | Positive | Dihexose (Maltose/Sucrose) | Unknown | Unknown | 4.92 | 365.11 | 123.01 | 243.34 | 0.01 | 0.04 | 1.98 | 0.98 |
| 1045 | Positive | Unknown | Unknown | Unknown | 4.91 | 381.08 | 37.34 | 87.6 | 0 | 0.04 | 2.35 | 1.23 |
| 1430 | Positive | Unknown | Melezitose | Unknown | 5.14 | 522.2 | 20.53 | 83.62 | 0 | 0 | 4.07 | 2.03 |
| 1436 | Positive | Unknown | Unknown | Unknown | 5.15 | 524.15 | 5.41 | 89.06 | 0.01 | 0.02 | 16.45 | 4.04 |
| 1445 | Positive | Unknown | Unknown | Unknown | 5.17 | 527.16 | 20.64 | 70.2 | 0 | 0 | 3.4 | 1.77 |
| 1461 | Positive | Unknown | Unknown | Unknown | 4.92 | 533.16 | 53.45 | 239.8 | 0.01 | 0.03 | 4.49 | 2.17 |
| 1879 | Positive | Unknown | Unknown | Unknown | 4.92 | 704.21 | 10.77 | 91.55 | 0 | 0.03 | 8.5 | 3.09 |
| 2049 | Positive | Unknown | Unknown | Unknown | 14.02 | 786.44 | 104.64 | 231.98 | 0.02 | 0.04 | 2.22 | 1.15 |
| 2138 | Positive | Unknown | Unknown | Unknown | 14.38 | 916.51 | 45.4 | 80.8 | 0.02 | 0.04 | 1.78 | 0.83 |
| 2148 | Positive | Unknown | Unknown | Unknown | 14.28 | 930.48 | 61.44 | 159.72 | 0.01 | 0.01 | 2.6 | 1.38 |

## Table S8: Differentially accumulated compounds in the A compartment of black oat grown with black grass as interspecific neighbor (BO-G/A) compared to black oat grown alone (BO-0/A) in the black grass experimental set (G). Data are from the second 24-hour methanolic re-exudation extract. Compounds were identified using multiple confidence levels according to the Schymanski scale: Level 1 identification was based on matching to authentic standards (annotation "w/o MS2" indicates no confident MS2 spectral match); Level 2a identification was obtained from spectral library matching via MS-FINDER and GNPS; Level 2b identification was predicted using MS-FINDER and SIRIUS CSI:FingerID. Statistical significance was determined by Welch's t-test (FDR-corrected p < 0.05) and |log2 fold change| > 0.6.

| Alignment ID | Ionization Mode | Level 1 Identification | Level 2a Identification | Level 2b Identification | Average Rt(min) | Average Mz | BO-0/A | BO-G/A | p.value | fdr adjusted p.value | Fold Change | Log2 fold change |
| --- | --- | --- | --- | --- | --- | --- | --- | --- | --- | --- | --- | --- |
| 139 | Negative | Unknown | Unknown | Unknown | 4.81 | 195.05 | 411.33 | 653.32 | 0.02 | 0.04 | 1.59 | 0.67 |
| 173 | Negative | Unknown | Unknown | Unknown | 13.69 | 215.03 | 43.4 | 76.9 | 0.01 | 0.02 | 1.77 | 0.83 |
| 183 | Negative | w/o MS2:N-acetyl-D-mannosamine | Unknown | N-Acetylgalactosamine | 4.91 | 220.08 | 223.99 | 476.15 | 0.01 | 0.02 | 2.13 | 1.09 |
| 233 | Negative | Unknown | Unknown | Unknown | 12.39 | 245.09 | 37.17 | 63.77 | 0.02 | 0.05 | 1.72 | 0.78 |
| 246 | Negative | Unknown | Unknown | Unknown | 9.05 | 252.02 | 405.65 | 706.89 | 0.02 | 0.05 | 1.74 | 0.8 |
| 254 | Negative | Unknown | Unknown | Unknown | 3.06 | 257.02 | 16.11 | 30.91 | 0.02 | 0.03 | 1.92 | 0.94 |
| 276 | Negative | Unknown | Unknown | Unknown | 9.1 | 267.02 | 253.95 | 453.71 | 0.01 | 0.03 | 1.79 | 0.84 |
| 300 | Negative | Unknown | Unknown | Unknown | 13.39 | 279.01 | 186.7 | 314.47 | 0.01 | 0.02 | 1.68 | 0.75 |
| 312 | Negative | Unknown | Unknown | Unknown | 9.95 | 285.06 | 187.08 | 296.33 | 0.02 | 0.04 | 1.58 | 0.66 |
| 398 | Negative | Unknown | Unknown | Unknown | 12.62 | 321.1 | 247.44 | 495.59 | 0.01 | 0.02 | 2 | 1 |
| 431 | Negative | Unknown | Unknown | Unknown | 11.02 | 335.13 | 56.15 | 118.29 | 0 | 0.01 | 2.11 | 1.08 |
| 436 | Negative | Unknown | Unknown | Unknown | 9.44 | 337.11 | 99.28 | 167.43 | 0.01 | 0.03 | 1.69 | 0.75 |
| 449 | Negative | Unknown | Unknown | Unknown | 9.53 | 343.1 | 150.07 | 288.49 | 0 | 0.02 | 1.92 | 0.94 |
| 451 | Negative | Unknown | Unknown | Unknown | 9.15 | 344.1 | 92.21 | 148.84 | 0.02 | 0.05 | 1.61 | 0.69 |
| 473 | Negative | Unknown | Unknown | Unknown | 9.96 | 355.05 | 33.69 | 62.38 | 0.01 | 0.03 | 1.85 | 0.89 |
| 476 | Negative | Unknown | Unknown | Unknown | 5.17 | 355.09 | 74.91 | 138.6 | 0.01 | 0.02 | 1.85 | 0.89 |
| 519 | Negative | Unknown | Unknown | Unknown | 9.11 | 373.08 | 2062.29 | 3302.24 | 0.02 | 0.05 | 1.6 | 0.68 |
| 520 | Negative | Unknown | Unknown | Unknown | 8.63 | 373.08 | 63.79 | 109.82 | 0.02 | 0.05 | 1.72 | 0.78 |
| 552 | Negative | Unknown | Unknown | Unknown | 11.91 | 387.09 | 50.09 | 113.78 | 0 | 0 | 2.27 | 1.18 |
| 592 | Negative | Unknown | Unknown | Unknown | 9.12 | 405.05 | 29.84 | 50.93 | 0.01 | 0.02 | 1.71 | 0.77 |
| 603 | Negative | Unknown | Unknown | Unknown | 11.18 | 413.15 | 49.05 | 99.12 | 0 | 0.01 | 2.02 | 1.01 |
| 604 | Negative | Unknown | Unknown | Unknown | 12.07 | 413.15 | 2788.35 | 5095.02 | 0.01 | 0.03 | 1.83 | 0.87 |
| 629 | Negative | Unknown | Unknown | Unknown | 9.47 | 423.11 | 64.82 | 117.97 | 0.01 | 0.02 | 1.82 | 0.86 |
| 633 | Negative | Unknown | Unknown | Unknown | 12.91 | 427.16 | 244.22 | 400.56 | 0.01 | 0.02 | 1.64 | 0.71 |
| 645 | Negative | Unknown | Unknown | Unknown | 9.48 | 431.12 | 205.27 | 446.97 | 0 | 0.02 | 2.18 | 1.12 |
| 650 | Negative | Unknown | Unknown | Unknown | 9.47 | 433.14 | 758.22 | 1573.34 | 0 | 0 | 2.08 | 1.05 |
| 659 | Negative | Unknown | Unknown | Unknown | 9.05 | 439.05 | 17.44 | 27.19 | 0.02 | 0.04 | 1.56 | 0.64 |
| 675 | Negative | Unknown | Unknown | Unknown | 9.94 | 447.15 | 54.82 | 89.92 | 0.02 | 0.04 | 1.64 | 0.71 |
| 680 | Negative | Unknown | Unknown | Unknown | 9.46 | 450.13 | 91.61 | 201.53 | 0 | 0.01 | 2.2 | 1.14 |
| 686 | Negative | Unknown | Unknown | Unknown | 13.02 | 451.22 | 127.53 | 253.89 | 0.02 | 0.04 | 1.99 | 0.99 |
| 691 | Negative | Unknown | Unknown | Unknown | 11.12 | 453.2 | 33.92 | 68.82 | 0.01 | 0.04 | 2.03 | 1.02 |
| 713 | Negative | Unknown | Unknown | Unknown | 9.35 | 461.13 | 10.57 | 18.5 | 0.02 | 0.04 | 1.75 | 0.81 |
| 714 | Negative | Unknown | Unknown | Unknown | 9.67 | 461.13 | 27.73 | 53.7 | 0.03 | 0.05 | 1.94 | 0.95 |
| 718 | Negative | Unknown | Unknown | Unknown | 10.56 | 462.19 | 1.25 | 2.83 | 0.02 | 0.04 | 2.28 | 1.19 |
| 720 | Negative | Unknown | Unknown | Unknown | 9.62 | 463.14 | 149.4 | 244.64 | 0.02 | 0.05 | 1.64 | 0.71 |
| 721 | Negative | Unknown | Unknown | Unknown | 9.08 | 463.15 | 163.38 | 299.65 | 0.01 | 0.03 | 1.83 | 0.88 |
| 739 | Negative | Unknown | Unknown | Unknown | 10.37 | 472.1 | 33.23 | 58.38 | 0.02 | 0.03 | 1.76 | 0.81 |
| 747 | Negative | Unknown | Unknown | Unknown | 12.08 | 476.14 | 318.64 | 638.46 | 0.01 | 0.02 | 2 | 1 |
| 788 | Negative | Unknown | Unknown | PubChem:(117768116) | 12.81 | 498.18 | 55.3 | 100.33 | 0.02 | 0.04 | 1.81 | 0.86 |
| 799 | Negative | Unknown | Unknown | Unknown | 9.05 | 505.05 | 64.95 | 111.65 | 0.02 | 0.03 | 1.72 | 0.78 |
| 800 | Negative | Unknown | Unknown | Unknown | 9.47 | 505.07 | 59.66 | 122.22 | 0.01 | 0.02 | 2.05 | 1.03 |
| 801 | Negative | Unknown | Unknown | Unknown | 9.71 | 505.16 | 9.27 | 18.39 | 0.02 | 0.03 | 1.98 | 0.99 |
| 802 | Negative | Unknown | Unknown | KEGG Mine | 9.47 | 505.16 | 98.74 | 184.89 | 0 | 0.01 | 1.87 | 0.9 |
| 808 | Negative | Unknown | Unknown | Unknown | 12.69 | 507.21 | 1056.05 | 1767.1 | 0.02 | 0.04 | 1.67 | 0.74 |
| 824 | Negative | Unknown | Unknown | Unknown | 4.76 | 515.13 | 63.18 | 132.11 | 0.02 | 0.04 | 2.09 | 1.06 |
| 836 | Negative | Unknown | Unknown | Unknown | 10.12 | 521.12 | 10.96 | 27.67 | 0.02 | 0.05 | 2.52 | 1.34 |
| 846 | Negative | Unknown | Unknown | Unknown | 13.7 | 525.19 | 17.57 | 31.8 | 0.02 | 0.05 | 1.81 | 0.86 |
| 856 | Negative | Unknown | Unknown | Unknown | 9.86 | 533.17 | 162.46 | 280.06 | 0.02 | 0.05 | 1.72 | 0.79 |
| 857 | Negative | Unknown | Unknown | Unknown | 12.15 | 533.17 | 18.47 | 31.93 | 0.02 | 0.04 | 1.73 | 0.79 |
| 860 | Negative | Unknown | Unknown | Unknown | 9.11 | 535.06 | 47.22 | 87.01 | 0.01 | 0.04 | 1.84 | 0.88 |
| 862 | Negative | Unknown | Unknown | Unknown | 5.32 | 535.15 | 11.46 | 37.74 | 0.01 | 0.03 | 3.29 | 1.72 |
| 868 | Negative | Unknown | Unknown | Unknown | 4.84 | 537.17 | 95.41 | 230.42 | 0.01 | 0.02 | 2.42 | 1.27 |
| 905 | Negative | Unknown | Unknown | Unknown | 4.89 | 562.2 | 38.27 | 100.17 | 0.02 | 0.04 | 2.62 | 1.39 |
| 916 | Negative | Unknown | Unknown | Unknown | 9.26 | 573.15 | 115.71 | 246.56 | 0 | 0 | 2.13 | 1.09 |
| 918 | Negative | Unknown | Unknown | Unknown | 11.43 | 575.2 | 39.83 | 73.26 | 0.01 | 0.02 | 1.84 | 0.88 |
| 993 | Negative | Unknown | Unknown | Unknown | 8.13 | 668.2 | 20.67 | 47.15 | 0.01 | 0.01 | 2.28 | 1.19 |
| 1016 | Negative | Unknown | Unknown | Unknown | 11.39 | 687.19 | 70.65 | 121.72 | 0.01 | 0.02 | 1.72 | 0.78 |
| 1026 | Negative | Unknown | Unknown | PubChem:(101607589) | 12.71 | 697.23 | 58.61 | 116.66 | 0.01 | 0.04 | 1.99 | 0.99 |
| 1064 | Negative | Unknown | Unknown | Unknown | 12.04 | 725.32 | 22.61 | 42.11 | 0.01 | 0.03 | 1.86 | 0.9 |
| 1068 | Negative | Unknown | Unknown | Unknown | 13 | 727.21 | 54.05 | 109.22 | 0 | 0.03 | 2.02 | 1.02 |
| 1072 | Negative | Unknown | Unknown | Unknown | 13.03 | 729.2 | 5.33 | 9.27 | 0.02 | 0.04 | 1.74 | 0.8 |
| 1163 | Negative | Unknown | Unknown | Unknown | 13.06 | 803.2 | 2.52 | 4.19 | 0.01 | 0.02 | 1.66 | 0.73 |
| 1193 | Negative | Unknown | Unknown | Unknown | 12.07 | 827.3 | 60.25 | 238.15 | 0.01 | 0.02 | 3.95 | 1.98 |
| 1211 | Negative | Unknown | Unknown | Unknown | 11.33 | 849.09 | 1.22 | 1.94 | 0.02 | 0.04 | 1.59 | 0.67 |
| 1243 | Negative | Unknown | Unknown | Unknown | 13.41 | 943.29 | 222.65 | 367.3 | 0.02 | 0.05 | 1.65 | 0.72 |
| 1328 | Negative | Unknown | Unknown | Unknown | 13.12 | 1375.39 | 36.63 | 68.56 | 0.01 | 0.03 | 1.87 | 0.9 |
| 161 | Positive | Unknown | Choline; LC-ESI-QTOF; MS2; CE | Unknown | 3.57 | 104.11 | 153.19 | 337.9 | 0.01 | 0.02 | 2.21 | 1.14 |
| 233 | Positive | Unknown | Unknown | Unknown | 12.09 | 127.04 | 80.4 | 143.23 | 0.01 | 0.03 | 1.78 | 0.83 |
| 335 | Positive | Unknown | Unknown | Unknown | 9.89 | 155.07 | 33.2 | 59.39 | 0.01 | 0.03 | 1.79 | 0.84 |
| 369 | Positive | Unknown | Unknown | Unknown | 12.09 | 163.06 | 252.5 | 433.76 | 0.02 | 0.04 | 1.72 | 0.78 |
| 383 | Positive | Unknown | Unknown | Unknown | 9.72 | 167.07 | 9.12 | 15.19 | 0.01 | 0.03 | 1.67 | 0.74 |
| 389 | Positive | Unknown | Unknown | Adermine | 9.11 | 170.08 | 24.9 | 46.13 | 0.02 | 0.04 | 1.85 | 0.89 |
| 469 | Positive | Unknown | Unknown | Unknown | 4.74 | 192.09 | 84 | 182.51 | 0 | 0.01 | 2.17 | 1.12 |
| 509 | Positive | Unknown | Unknown | Unknown | 4.96 | 204.09 | 346.96 | 713.02 | 0.02 | 0.04 | 2.06 | 1.04 |
| 559 | Positive | Unknown | Unknown | N-Acetylgalactosamine | 4.99 | 222.1 | 2845.58 | 6688.03 | 0.01 | 0.02 | 2.35 | 1.23 |
| 577 | Positive | Unknown | Unknown | Unknown | 6.05 | 226.07 | 19.15 | 51.08 | 0 | 0 | 2.67 | 1.42 |
| 644 | Positive | Unknown | Unknown | Unknown | 6.05 | 244.08 | 62.03 | 136.9 | 0 | 0.01 | 2.21 | 1.14 |
| 673 | Positive | Unknown | Unknown | Unknown | 3.45 | 252.14 | 40.03 | 77.36 | 0.02 | 0.04 | 1.93 | 0.95 |
| 840 | Positive | Unknown | Unknown | Unknown | 9.47 | 295.1 | 77.02 | 155.01 | 0.01 | 0.03 | 2.01 | 1.01 |
| 874 | Positive | Unknown | Unknown | Unknown | 12.08 | 307.1 | 265.37 | 466.97 | 0.01 | 0.03 | 1.76 | 0.82 |
| 1110 | Positive | Unknown | Unknown | Unknown | 9.42 | 394.13 | 166.65 | 361.76 | 0.01 | 0.04 | 2.17 | 1.12 |
| 1119 | Positive | Unknown | Unknown | Unknown | 12.08 | 397.15 | 256.67 | 453.72 | 0.01 | 0.04 | 1.77 | 0.82 |
| 1141 | Positive | Unknown | Unknown | Unknown | 9.46 | 406.17 | 246.51 | 440.51 | 0.01 | 0.03 | 1.79 | 0.84 |
| 1197 | Positive | Unknown | Unknown | Unknown | 12.08 | 432.19 | 303.7 | 537.6 | 0.01 | 0.03 | 1.77 | 0.82 |
| 1206 | Positive | Unknown | Unknown | Unknown | 9.64 | 436.18 | 67.64 | 113.19 | 0.02 | 0.04 | 1.67 | 0.74 |
| 1347 | Positive | Unknown | Unknown | Unknown | 12.86 | 486.25 | 62.03 | 133.72 | 0.01 | 0.02 | 2.16 | 1.11 |
| 1365 | Positive | Unknown | Unknown | Unknown | 12.7 | 491.21 | 120.61 | 212.06 | 0.01 | 0.03 | 1.76 | 0.81 |
| 1773 | Positive | Unknown | Unknown | Unknown | 8.14 | 670.22 | 44.34 | 102.92 | 0.01 | 0.02 | 2.32 | 1.21 |
| 1869 | Positive | Unknown | Unknown | Unknown | 12.7 | 716.28 | 80.54 | 156.11 | 0.01 | 0.03 | 1.94 | 0.95 |

## Table S9: Number of compounds identified to each confidence level for redroot pigweed second methanolic extract samples

| Ionization Mode | Annotation | Number Of Compounds |
| --- | --- | --- |
| Negative | Total Compounds | 952 |
| Negative | Total Fragmented | 846 |
| Negative | Confidence Level 3 | 566 |
| Negative | Confidence Level 2 | 56 |
| Negative | Confidence Level 2b | 37 |
| Negative | Confidence Level 2a | 28 |
| Negative | Confidence Level 1 | 12 |
| Positive | Total Compounds | 597 |
| Positive | Total Fragmented | 514 |
| Positive | Confidence Level 3 | 292 |
| Positive | Confidence Level 2 | 45 |
| Positive | Confidence Level 2b | 34 |
| Positive | Confidence Level 2a | 22 |
| Positive | Confidence Level 1 | 8 |

## Table S10: Number of compounds identified to each confidence level for blackgrass second methanolic extract samples

| Ionization Mode | Annotation | Number Of Compounds |
| --- | --- | --- |
| Negative | Total Compounds | 674 |
| Negative | Total Fragmented | 581 |
| Negative | Confidence Level 3 | 438 |
| Negative | Confidence Level 2 | 30 |
| Negative | Confidence Level 2b | 25 |
| Negative | Confidence Level 2a | 13 |
| Negative | Confidence Level 1 | 15 |
| Positive | Total Compounds | 620 |
| Positive | Total Fragmented | 521 |
| Positive | Confidence Level 3 | 297 |
| Positive | Confidence Level 2 | 45 |
| Positive | Confidence Level 2b | 37 |
| Positive | Confidence Level 2a | 17 |
| Positive | Confidence Level 1 | 8 |

## Table S11: Complete list of compounds identified in the second 24-hour methanolic re-exudation extract from the redroot pigweed experimental set (P). Compounds were annotated according to the Schymanski confidence scale: Level 1 based on matching retention time and MS2 spectra to authentic standards; Level 2a from spectral library matches via MS-FINDER and GNPS; Level 2b from in silico predictions using MS-FINDER and SIRIUS CSI:FingerID.

| **Ionization Mode** | **Alignment ID** | **Average Rt(min)** | **Average Mz** | **Level 1 Identification** | **Level 2a Identification** | **Level 2b Identification** |
| --- | --- | --- | --- | --- | --- | --- |
| Negative | 0 | 7.501 | 57.034 | Unknown | NA | Propylene glycol |
| Negative | 17 | 5.499 | 71.01338 | Unknown | NA | L-Lactic acid |
| Negative | 124 | 3.011 | 117.019 | Succinic acid | SUCCINIC ACID | NA |
| Negative | 141 | 3.164 | 128.0351 | Pyroglutamic acid | NA | Pyroglutamic acid |
| Negative | 146 | 7.474 | 129.0189 | Unknown | Mesaconic acid; LC-ESI-QTOF; MS2; CE | NA |
| Negative | 156 | 2.913 | 131.046 | Asparagine | NA | L-Asparagine |
| Negative | 157 | 3.138 | 132.0299 | Aspartic Acid | Aspartate; LC-ESI-ITFT; MS2; CE 85.0 eV; [M-H]- | L-Aspartic acid |
| Negative | 160 | 5.48 | 133.0141 | Malic acid PGC | D-(+)-Malic acid | L-Malic acid |
| Negative | 167 | 4.275 | 135.0291 | Threonic Acid | NA | NA |
| Negative | 173 | 13.505 | 137.0237 | Salicylic acid | P-HYDROXYBENZOIC ACID | NA |
| Negative | 186 | 3.161 | 145.0616 | Glutamine | Glutamine; LC-ESI-ITFT; MS2; CE 75.0 eV; [M-H]- | L-Glutamine |
| Negative | 189 | 3.56 | 146.0455 | Glutamic Acid | L-Glutamic acid; LC-ESI-QTOF; MS2; CE | L-Glutamic acid |
| Negative | 194 | 4.886 | 149.0449 | Unknown | NA | D-Xylose |
| Negative | 200 | 13.502 | 153.0187 | Unknown | 3,4-DIHYDROXYBENZOIC ACID | NA |
| Negative | 258 | 4.89 | 173.0088 | Unknown | DEHYDROASCORBIC ACID - 40.0 eV | NA |
| Negative | 260 | 5.592 | 173.0446 | Shikimic acid PGC | NA | Shikimic acid |
| Negative | 283 | 5.312 | 179.0552 | Unknown | NA | myo-Inositol |
| Negative | 287 | 6.866 | 180.0659 | Unknown | NA | L-Tyrosine |
| Negative | 291 | 3.415 | 181.0707 | Unknown | NA | Galactitol |
| Negative | 315 | 8.979 | 191.019 | Unknown | NA | Citric acid |
| Negative | 317 | 7.472 | 191.0193 | Unknown | NA | Citric acid |
| Negative | 318 | 6.392 | 191.0194 | Isocitric acid PGC | ISOCITRIC ACID | Citric acid |
| Negative | 322 | 5.178 | 191.056 | Unknown | D-(-)-Quinic acid; LC-ESI-QTOF; MS2; CE | Quinic acid |
| Negative | 336 | 4.98 | 195.0526 | Unknown | 1726059 - 40.0 eV | Galactonic acid |
| Negative | 370 | 10.741 | 206.0819 | Unknown | NA | Afalanina |
| Negative | 407 | 11.677 | 219.0766 | Unknown | 5-HYDROXY-TRYPTOPHAN | NA |
| Negative | 764 | 5.097 | 341.1107 | Dihexose (Maltose/Sucrose) | SUCROSE | Sucrose |
| Negative | 837 | 9.828 | 371.0973 | Unknown | NA | COCONUT:(CNP0115847 CNP0131848);PubChem:(45783079 51693489 51693491 101793099 133556366);SuperNatural:(SN00033299 SN00033300);ZINC bio:(ZINC35457686 ZINC35457690);additional;Training Set |
| Negative | 838 | 10.416 | 371.0978 | Unknown | (2S,3S,4S,5R,6R)-6-(3-benzoyloxy-2-hydroxypropoxy)-3,4,5-trihydroxyoxane-2-carboxylic acid | COCONUT:(CNP0115847 CNP0131848);PubChem:(45783079 51693489 51693491 101793099 133556366);SuperNatural:(SN00033299 SN00033300);ZINC bio:(ZINC35457686 ZINC35457690);additional;Training Set |
| Negative | 881 | 13.386 | 385.0921 | Unknown | 8-5'-Benzofuran-diferulic acid | NA |
| Negative | 891 | 4.976 | 387.1164 | Unknown | alpha,alpha-Trehalose - 40.0 eV | NA |
| Negative | 915 | 13.199 | 393.1745 | Unknown | NA | PubChem:(134883503) |
| Negative | 928 | 9.758 | 399.0923 | Unknown | NA | COCONUT:(CNP0170379);PubChem:(51136464);SuperNatural:(SN00039051);ZINC bio:(ZINC49180912);additional;Training Set |
| Negative | 935 | 8.486 | 403.0902 | Unknown | NA | PubChem:(46222441) |
| Negative | 960 | 11.888 | 413.145 | Unknown | NA | COCONUT:(CNP0159349 CNP0218833);Natural Products:(UNPD202200);PubChem:(38363079 38363084 45360328 44715457 125416071 125416072 125416073 125416074);SuperNatural:(SN00032625 SN00030299 SN00032624 SN00030300 SN00032623 SN00032622);ZINC bio:(ZINC31169353 ZINC31169357 ZINC35454681 ZINC35454685 ZINC35454688 ZINC35454690);Training Set |
| Negative | 961 | 11.055 | 413.1454 | Unknown | NA | COCONUT:(CNP0159349 CNP0218833);Natural Products:(UNPD202200);PubChem:(38363079 38363084 45360328 44715457 125416071 125416072 125416073 125416074);SuperNatural:(SN00032625 SN00030299 SN00032624 SN00030300 SN00032623 SN00032622);ZINC bio:(ZINC31169353 ZINC31169357 ZINC35454681 ZINC35454685 ZINC35454688 ZINC35454690);Training Set |
| Negative | 1033 | 9.105 | 435.0597 | Unknown | NA | PubChem:(102531776) |
| Negative | 1048 | 13.76 | 439.2544 | Unknown | NA | PubChem:(52919663 75954071) |
| Negative | 1103 | 9.189 | 460.1461 | Unknown | NA | PubChem:(53359647) |
| Negative | 1263 | 12.06 | 535.1809 | Unknown | NA | PubChem:(89189281) |
| Negative | 1271 | 5.322 | 539.139 | Unknown | RAFFINOSE CollisionEnergy:102040 | NA |
| Negative | 1305 | 12.951 | 557.1855 | Unknown | NA | 3-[[4,6-bis[bis(2-carboxyethyl)amino]-1,3,5-triazin-2-yl]-(2-carboxyethyl)amino]propanoic acid |
| Negative | 1364 | 10.413 | 613.2341 | Unknown | NA | PubChem:(130251683) |
| Negative | 1449 | 13.876 | 677.3527 | Unknown | NA | COCONUT:(CNP0090331) |
| Negative | 1464 | 15.17 | 686.4767 | Unknown | PE(16:1_16:1) - (2-aminoethoxy)[2,3-di[hexadec-9-enoyloxy]propoxy]phosphinic acid | NA |
| Negative | 1471 | 15.264 | 688.4916 | Unknown | PE(16:0/16:1); [M-H]- C37H71N1O8P1 | NA |
| Negative | 1499 | 14.523 | 707.4866 | Unknown | PG(15:0/16:0); [M-H]- C37H72O10P1 | NA |
| Negative | 1508 | 15.071 | 711.4915 | Unknown | NA | benzyl N-[(2S)-6-amino-1-[3-[4-[3-[[(2S)-6-amino-2-(phenylmethoxycarbonylamino)hexanoyl]amino]propylamino]butylamino]propylamino]hexan-2-yl]carbamate |
| Negative | 1514 | 15.299 | 714.5074 | Unknown | PE(16:1/18:1); [M-H]- C39H73N1O8P1 | NA |
| Negative | 1523 | 14.496 | 719.4865 | Unknown | Massbank:LQB00238 PG 32:1 | NA |
| Negative | 1560 | 14.5 | 745.5029 | Unknown | PG(16:0/18:2); [M-H]- C40H74O10P1 | NA |
| Negative | 1565 | 14.531 | 747.5177 | Unknown | Massbank:LQB00241 PG 34:1 | NA |
| Negative | 1606 | 14.503 | 771.517 | Unknown | PG(18:1/18:2); [M-H]- C42H76O10P1 | NA |
| Negative | 1607 | 14.536 | 773.5337 | Unknown | Massbank:LQB00261 PG 36:2 | NA |
| Negative | 1678 | 14.2 | 833.5185 | Unknown | Massbank:LQB00679 PI 34:2 | NA |
| Negative | 1781 | 13.508 | 1019.505 | Unknown | NA | PubChem:(10581887) |
| Positive | 15 | 3.022 | 58.0649 | Unknown | NA | 2-Methylaziridine |
| Positive | 102 | 6.954 | 86.09585 | Unknown | Piperidine; LC-ESI-QTOF; MS2; CE | Piperidine |
| Positive | 162 | 3.5 | 104.1073 | Unknown | Choline; LC-ESI-QTOF; MS2; CE | NA |
| Positive | 201 | 3.171 | 116.0708 | Proline PGC | Proline; LC-ESI-ITFT; MS2; CE 85.0 eV; [M+H]+ | NA |
| Positive | 213 | 3.155 | 118.0865 | Unknown | Betaine; LC-ESI-QTOF; MS2; CE | NA |
| Positive | 241 | 3.133 | 130.0499 | Pyroglutamic acid | L-5-Oxoproline | Pyroglutamic acid |
| Positive | 254 | 2.909 | 133.0612 | Asparagine | NA | agedoite |
| Positive | 260 | 3.142 | 134.0454 | Unknown | Aspartic acid; LC-ESI-QTOF; MS2; CE | L-Aspartic acid |
| Positive | 263 | 13.345 | 134.0606 | Unknown | Massbank:TUE00032 5-Methylbenzotriazole | Acetaminophen |
| Positive | 267 | 6.513 | 136.0755 | Unknown | NA | 2-Phenylacetamide |
| Positive | 274 | 4.966 | 138.0549 | Unknown | NA | 2-Aminobenzoic acid |
| Positive | 275 | 10.436 | 138.0917 | Unknown | NA | Tyramine |
| Positive | 302 | 3.13 | 147.0765 | Glutamine | L-Glutamine; LC-ESI-QTOF; MS2; CE | L-Glutamine |
| Positive | 303 | 5.083 | 147.0771 | Unknown | NA | L-Glutamine |
| Positive | 305 | 3.51 | 148.0609 | Glutamic acid | L-Glutamic acid; LC-ESI-QTOF; MS2; CE | L-Glutamic acid |
| Positive | 316 | 10.053 | 152.0707 | Unknown | NA | Acetaminophen |
| Positive | 329 | 5.027 | 156.0766 | Histidine | L-Histidine; LC-ESI-QTOF; MS2; CE | L-Histidine |
| Positive | 347 | 10.911 | 161.06 | Unknown | NA | 6-Methylcoumarin |
| Positive | 360 | 6.556 | 165.0542 | Unknown | NA | 4-Hydroxycinnamic acid |
| Positive | 363 | 10.503 | 166.0867 | Unknown | NA | Sabiden |
| Positive | 364 | 9.565 | 166.0867 | Unknown | NA | L-Phenylalanine |
| Positive | 382 | 4.658 | 175.1079 | Arginine | NA | NA |
| Positive | 384 | 9.14 | 176.07 | Unknown | NA | Indoleacetic acid |
| Positive | 410 | 6.514 | 182.0812 | Unknown | Tyrosine; LC-ESI-ITFT; MS2; CE 45.0 eV; [M+H]+ | L-Tyrosine |
| Positive | 418 | 2.862 | 184.0731 | Unknown | NA | phosphocholine |
| Positive | 495 | 13.153 | 205.0971 | Unknown | L-Tryptophan; LC-ESI-QTOF; MS2; CE | NA |
| Positive | 530 | 8.93 | 220.1189 | Unknown | pantothenic acid CollisionEnergy:205060 | Pantothenic acid |
| Positive | 534 | 11.436 | 221.092 | Unknown | NA | 5-Hydroxy-L-tryptophan |
| Positive | 538 | 4.987 | 222.0976 | Unknown | NA | N-Acetylgalactosamine |
| Positive | 664 | 3.472 | 258.1111 | Unknown | ReSpect:PM018116 sn-Glycero-3-phosphocholine | Gliatilin (TN) |
| Positive | 923 | 4.608 | 336.1408 | Unknown | NA | Asn-agam |
| Positive | 990 | 4.806 | 360.1506 | Unknown | Spectral Match to D-(+)-Trehalose from NIST14 | NA |
| Positive | 1000 | 4.916 | 365.1062 | Dihexose (Maltose/Sucrose) | NA | NA |
| Positive | 1053 | 9.272 | 384.1148 | Unknown | NA | Succinoadenosine |
| Positive | 1149 | 9.065 | 422.1119 | Unknown | NA | PubChem:(101229455) |
| Positive | 1246 | 12.708 | 454.229 | Unknown | NA | PubChem:(117619884) |
| Positive | 1294 | 14.725 | 471.3955 | Unknown | NA | N-Arachidoyl-5-hydroxytryptamine |
| Positive | 1430 | 5.142 | 522.2022 | Unknown | Melezitose | NA |
| Positive | 1831 | 15.087 | 688.493 | Unknown | Spectral Match to 1,2-Dipalmitoleoyl-sn-glycero-3-phosphoethanolamine from NIST14 | NA |
| Positive | 1913 | 15.191 | 716.523 | Unknown | Spectral Match to 2-Linoleoyl-1-palmitoyl-sn-glycero-3-phosphoethanolamine from NIST14 | NA |
| Positive | 1997 | 14.562 | 752.5299 | Unknown | NA | Dien-microgonotropen-c |
| Positive | 2010 | 15.27 | 758.5715 | Unknown | PC(16:1/18:1); [M+H]+ C42H81N1O8P1 | NA |
| Positive | 2045 | 15.208 | 782.5713 | Unknown | Spectral Match to 1,2-Dilinoleoyl-sn-glycero-3-phosphocholine from NIST14 | NA |
| Positive | 2050 | 15.363 | 786.6017 | Unknown | PC(18:1/18:1); [M+H]+ C44H85N1O8P1 | PC(18:1(9Z)/18:1(9Z)) |
| Positive | 2061 | 14.556 | 797.5313 | Unknown | Spectral Match to 1,2-Dioleoyl-sn-glycero-3-phospho-rac-1-glycerol from NIST14 | NA |
| Positive | 2099 | 13.156 | 841.423 | Unknown | NA | COCONUT:(CNP0326190);Natural Products:(UNPD89153);PubChem:(24066892 102464927);SuperNatural:(SN00293878) |
| Positive | 2102 | 14.725 | 842.6728 | Unknown | NA | COCONUT:(CNP0277004);Natural Products:(UNPD147896);PubChem:(23427479);SuperNatural:(SN00337084) |

## Table S12: Complete list of compounds identified in the second 24-hour methanolic re-exudation extract from the black grass experimental set (G). Compounds were annotated according to the Schymanski confidence scale: Level 1 based on matching retention time and MS2 spectra to authentic standards (annotation "w/o MS2" indicates no confident spectral match; if Level 2 identification conflicts with Level 1, the Level 2 annotation should be used); Level 2a from spectral library matches via MS-FINDER and GNPS; Level 2b from in silico predictions using MS-FINDER and SIRIUS CSI:FingerID.

| **Ionization Mode** | **Alignment ID** | **Average Rt(min)** | **Average Mz** | **Level 1 Identification** | **Level 2a Identification** | **Level 2b Identification** |
| --- | --- | --- | --- | --- | --- | --- |
| Negative | 20 | 3.771 | 105.0196 | Unknown | D-(+)-Glyceric acid; LC-ESI-QTOF; MS2; CE | NA |
| Negative | 28 | 3.364 | 115.0032 | w/o MS2:Fumaric acid | NA | Fumaric acid |
| Negative | 35 | 3.086 | 117.0188 | Succinic acid | Succinic acid; LC-ESI-QTOF; MS2; CE | Succinic acid |
| Negative | 45 | 2.949 | 131.0458 | Asparagine | NA | NA |
| Negative | 47 | 3.193 | 132.0311 | Aspartic Acid | Aspartate; LC-ESI-ITFT; MS2; CE 85.0 eV; [M-H]- | NA |
| Negative | 50 | 5.307 | 133.0138 | Malic acid PGC | D-(+)-Malic acid; LC-ESI-QTOF; MS2; CE | L-Malic acid |
| Negative | 54 | 4.319 | 135.029 | Threonic Acid | NA | NA |
| Negative | 55 | 13.62 | 137.0249 | Salicylic acid | NA | 4-Hydroxybenzoic acid |
| Negative | 59 | 3.235 | 145.0622 | Glutamine | Glutamine; LC-ESI-ITFT; MS2; CE 80.0 eV; [M-H]- | L-Glutamine |
| Negative | 62 | 3.591 | 146.0466 | Glutamic Acid | L-Glutamic acid | NA |
| Negative | 88 | 5.352 | 173.0462 | Shikimic acid PGC | (-)-Shikimic acid | Shikimic acid |
| Negative | 103 | 5.103 | 179.0562 | Unknown | NA | D-Glucose |
| Negative | 125 | 3.149 | 191.019 | Citric acid | CITRIC ACID | Citric acid |
| Negative | 128 | 6.112 | 191.0208 | Isocitric acid PGC | ISOCITRIC ACID | Citric acid |
| Negative | 131 | 4.985 | 191.0559 | Unknown | D-(-)-Quinic acid; LC-ESI-QTOF; MS2; CE | Quinic acid |
| Negative | 143 | 10.801 | 197.0445 | Syringic acid | NA | NA |
| Negative | 183 | 4.91 | 220.0819 | w/o MS2:N-acetyl-D-mannosamine | NA | N-Acetylgalactosamine |
| Negative | 446 | 4.864 | 341.1099 | Dihexose (Maltose/Sucrose) | Sucrose | Sucrose |
| Negative | 498 | 5.337 | 365.0437 | Unknown | NA | [1,2,3-trihydroxy-1-[(3R,4S,5R,6R)-3,4,5-trihydroxy-6-(hydroxymethyl)oxan-2-yl]propoxy] hydrogen sulfate |
| Negative | 513 | 10.546 | 371.0975 | Unknown | NA | COCONUT:(CNP0115847 CNP0131848);PubChem:(45783079 51693489 51693491 101793099 133556366);SuperNatural:(SN00033299 SN00033300);ZINC bio:(ZINC35457686 ZINC35457690);additional;Training Set |
| Negative | 594 | 8.631 | 409.0444 | Unknown | NA | 2''-Deoxy-5''-adenylyl imidodiphosphate |
| Negative | 644 | 9.877 | 431.1203 | Unknown | NA | Licoagroside B |
| Negative | 771 | 12.986 | 489.1979 | Unknown | NA | PubChem:(135430392) |
| Negative | 788 | 12.812 | 498.1826 | Unknown | NA | PubChem:(117768116) |
| Negative | 802 | 9.467 | 505.1567 | Unknown | NA | KEGG Mine |
| Negative | 834 | 10.148 | 520.1682 | Unknown | NA | PubChem:(10075389) |
| Negative | 969 | 5.261 | 637.184 | Unknown | NA | PubChem:(54564545) |
| Negative | 1008 | 4.86 | 683.2268 | Unknown | PALATINOSE | NA |
| Negative | 1024 | 5.056 | 695.2246 | Unknown | NA | PubChem:(91847146) |
| Negative | 1026 | 12.706 | 697.2341 | Unknown | NA | PubChem:(101607589) |
| Negative | 1102 | 14.656 | 747.5154 | Unknown | [2,3-dihydroxypropoxy][3-(hexadecanoyloxy)-2-[octadec-9-enoyloxy]propoxy]phosphinic acid | NA |
| Negative | 1148 | 14.185 | 785.4107 | Unknown | NA | PubChem:(132018988) |
| Negative | 1151 | 14.721 | 787.547 | Unknown | NA | PubChem:(88641774) |
| Positive | 21 | 10.938 | 60.04413 | Unknown | NA | Acetamide |
| Positive | 26 | 2.959 | 60.08095 | Unknown | NA | Trimethylamine |
| Positive | 101 | 7.199 | 86.0963 | Unknown | Piperidine; LC-ESI-QTOF; MS2; CE | Piperidine |
| Positive | 131 | 4.87 | 97.02834 | Unknown | NA | 2-Furancarboxaldehyde |
| Positive | 161 | 3.569 | 104.1072 | Unknown | Choline; LC-ESI-QTOF; MS2; CE | NA |
| Positive | 199 | 3.247 | 116.0702 | Proline PGC | Proline; LC-ESI-ITFT; MS2; CE 85.0 eV; [M+H]+ | L-Proline |
| Positive | 231 | 9.123 | 127.0391 | Unknown | NA | 1,2,3-Trihydroxybenzene |
| Positive | 239 | 4.629 | 130.0499 | Unknown | NA | Pidolate |
| Positive | 241 | 3.221 | 130.0506 | Pyroglutamic acid | L-5-Oxoproline; LC-ESI-QTOF; MS2; CE | Pyroglutamic acid |
| Positive | 257 | 2.976 | 133.0608 | Asparagine | NA | agedoite |
| Positive | 262 | 3.231 | 134.0448 | Unknown | NA | aspartate |
| Positive | 264 | 13.451 | 134.0602 | Unknown | NA | Acetaminophen |
| Positive | 276 | 4.926 | 138.0548 | Unknown | NA | 2-Aminobenzoic acid |
| Positive | 293 | 2.955 | 143.0815 | Unknown | NA | 3-imidazol-1-ylpropane-1,2-diol |
| Positive | 296 | 5.397 | 144.0658 | Unknown | NA | Adipo-2,6-lactam |
| Positive | 309 | 6.724 | 147.0436 | Unknown | NA | Coumarin |
| Positive | 311 | 3.218 | 147.077 | Glutamine | L-Glutamine; LC-ESI-QTOF; MS2; CE | L-Glutamine |
| Positive | 313 | 3.587 | 148.061 | Glutamic acid | L-Glutamic acid; LC-ESI-QTOF; MS2; CE | L-Glutamic acid |
| Positive | 325 | 13.451 | 152.0709 | Unknown | NA | zlchem 1306 |
| Positive | 340 | 5.166 | 156.0769 | Histidine | Histidine; LC-ESI-QTOF; MS2; CE | histidin |
| Positive | 376 | 6.714 | 165.0541 | Unknown | NA | 4-Hydroxycinnamic acid |
| Positive | 379 | 10.654 | 166.086 | Unknown | NA | Sabiden |
| Positive | 380 | 9.643 | 166.0866 | Unknown | NA | L-Phenylalanine |
| Positive | 389 | 9.108 | 170.0814 | Unknown | NA | Adermine |
| Positive | 408 | 4.634 | 175.1081 | w/o MS2:Arginine | NA | N-Acetylornithine |
| Positive | 440 | 6.711 | 182.0809 | Unknown | TYROSINE | tyrosine |
| Positive | 551 | 9.024 | 220.1178 | Unknown | Massbank:LU087003 Pantothenate\|Pantothenic acid\|3-[[(2R)-2,4-dihydroxy-3,3-dimethylbutanoyl]amino]propanoic acid | vitamin B5 |
| Positive | 559 | 4.99 | 222.0974 | Unknown | NA | N-Acetylgalactosamine |
| Positive | 645 | 12.593 | 244.1657 | Unknown | NA | prolyllysine |
| Positive | 692 | 3.566 | 258.1108 | Unknown | sn-Glycero-3-phosphocholine; LC-ESI-QTOF; MS2; CE | NA |
| Positive | 716 | 10.063 | 263.1602 | Unknown | NA | Poly(ethylene glycol) ethyl ether methacrylate |
| Positive | 734 | 8.552 | 268.1044 | Unknown | NA | Adenosine |
| Positive | 745 | 10.972 | 270.1333 | Unknown | NA | COCONUT:(CNP0352172);Natural Products:(UNPD6299);PubChem:(57478618);SuperNatural:(SN00281451) |
| Positive | 757 | 13.999 | 272.2216 | Unknown | NA | acylglycine c:13 |
| Positive | 813 | 9.13 | 289.0923 | Unknown | 2-methyl-3-[(2S,3R,4S,5S,6R)-3,4,5-trihydroxy-6-(hydroxymethyl)oxan-2-yl]oxypyran-4-one | maltol beta-D-glucopyranoside |
| Positive | 894 | 8.727 | 313.0855 | Unknown | NA | Acetaminophen-2-mercapturate |
| Positive | 1003 | 10.649 | 352.2231 | Unknown | NA | PubChem:(129847081);COCONUT:(CNP0112721) |
| Positive | 1022 | 4.838 | 360.1508 | Unknown | Spectral Match to Palatinose from NIST14 | NA |
| Positive | 1032 | 4.873 | 365.106 | Dihexose (Maltose/Sucrose) | NA | NA |
| Positive | 1187 | 14.97 | 427.3861 | Unknown | NA | Lanosterin |
| Positive | 1202 | 12.715 | 433.1129 | Unknown | Spectral Match to Isovitexin from NIST14 | NA |
| Positive | 1321 | 10.161 | 476.1763 | Unknown | NA | (2R)-2-{[6-O-(beta-D-glucopyranosyl)-beta-D-glucopyranosyl]oxy}-2-phenylethanamide |
| Positive | 1813 | 15.161 | 688.4927 | Unknown | Spectral Match to 1,2-Dipalmitoleoyl-sn-glycero-3-phosphoethanolamine from NIST14 | NA |
| Positive | 1820 | 15.238 | 690.5082 | Unknown | PE-DAG (16:0/16:1) | NA |
| Positive | 1956 | 15.359 | 758.571 | Unknown | Spectral Match to 1-Hexadecanoyl-2-octadecadienoyl-sn-glycero-3-phosphocholine from NIST14 | NA |
| Positive | 1986 | 15.297 | 782.5698 | Unknown | Spectral Match to 1,2-Dilinoleoyl-sn-glycero-3-phosphocholine from NIST14 | NA |


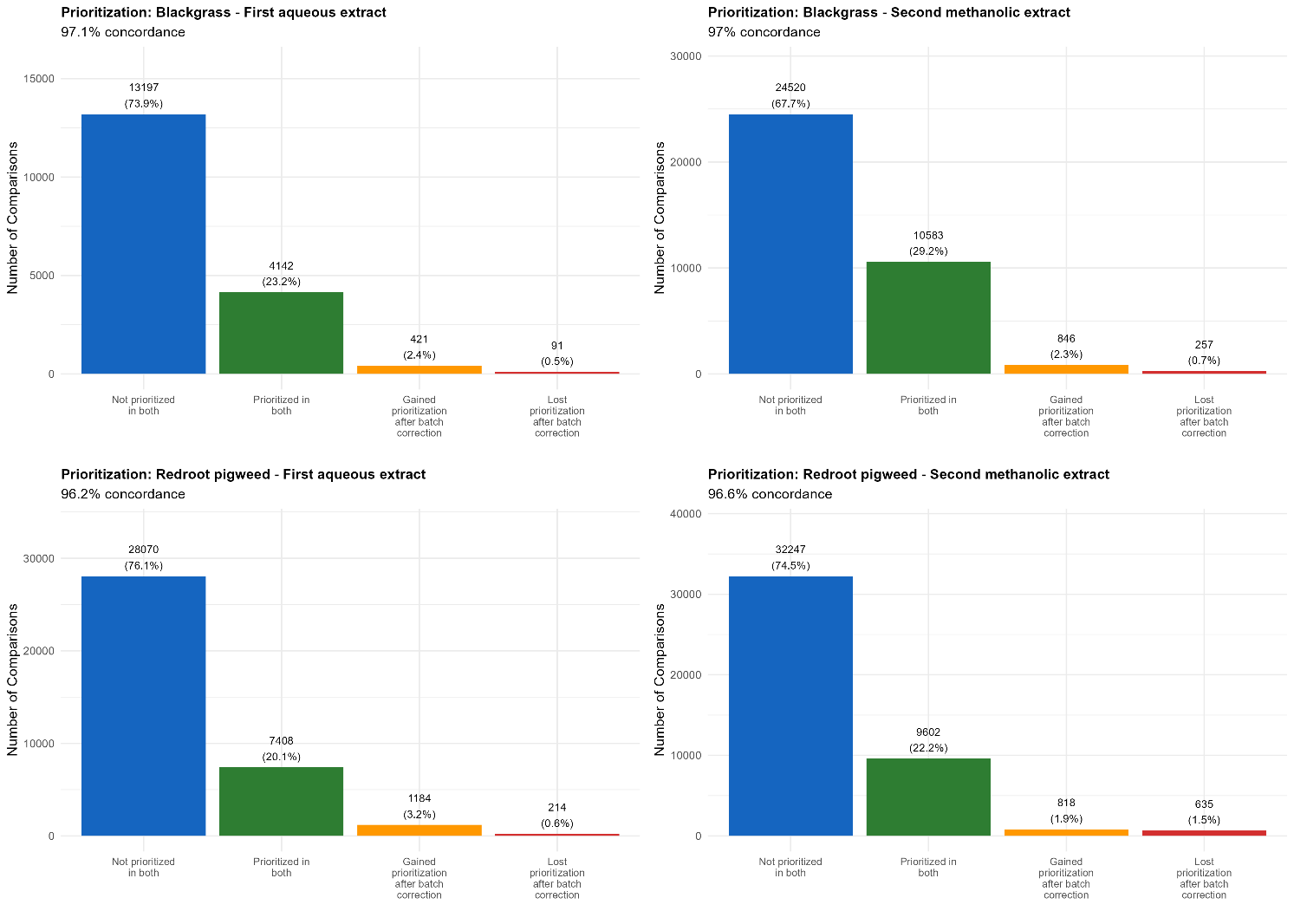


## Figure S1: Concordance of metabolite prioritization before and after batch correction using a linear mixed-effects model with batch as a random intercept. Bar plots show the number and percentage of pairwise metabolite comparisons classified as: not prioritized in both analyses (blue), prioritized in both (green), gained prioritization after batch correction (orange), or lost prioritization after correction (red).


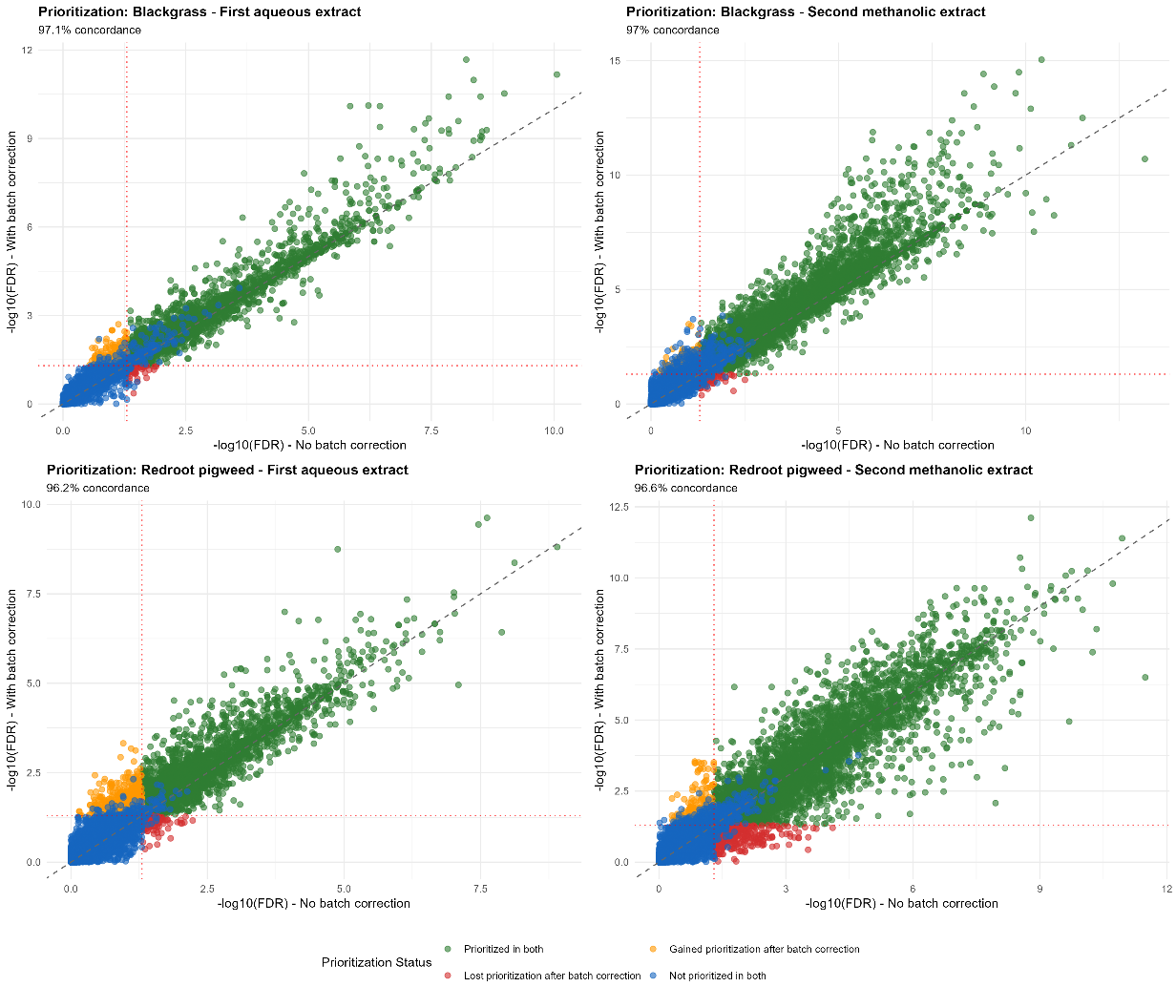


## Figure S2: Correlation of FDR-corrected p-values before and after batch correction using a linear mixed-effects model. Scatter plots show –log10(FDR) values from the original analysis (x-axis) versus after batch correction with trial as a random intercept (y-axis) for each dataset. Colors indicate prioritization status: not prioritized in either analysis (blue), prioritized in both (green), gained prioritization after correction (orange), or lost prioritization after correction (red). Blue points (not prioritized) are found in all quadrants of the plot, as metabolites may exceed the FDR significance threshold but fail to meet the fold change criterion (|log₂FC| > 0.6), thereby losing prioritization status. The dashed line represents the identity line (y = x). High correlation along the identity line indicates that batch correction has minimal impact on statistical significance rankings.


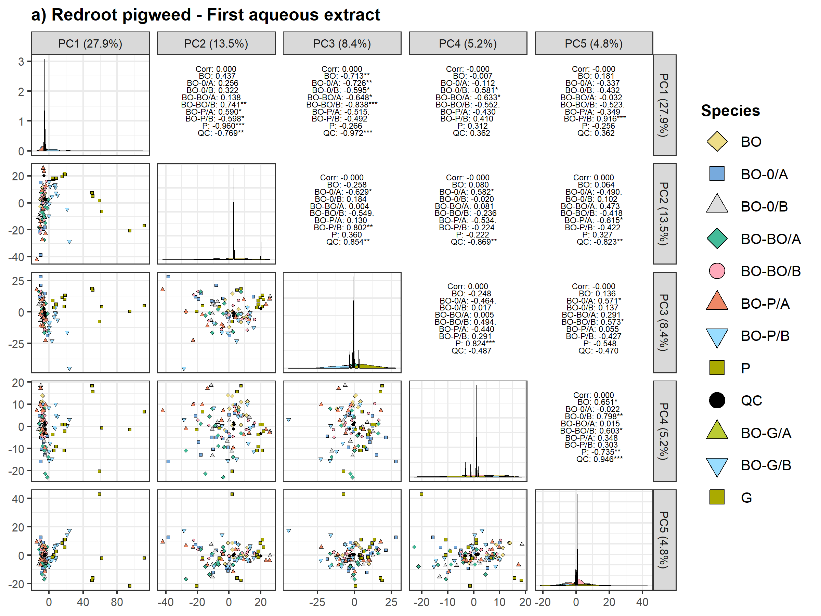

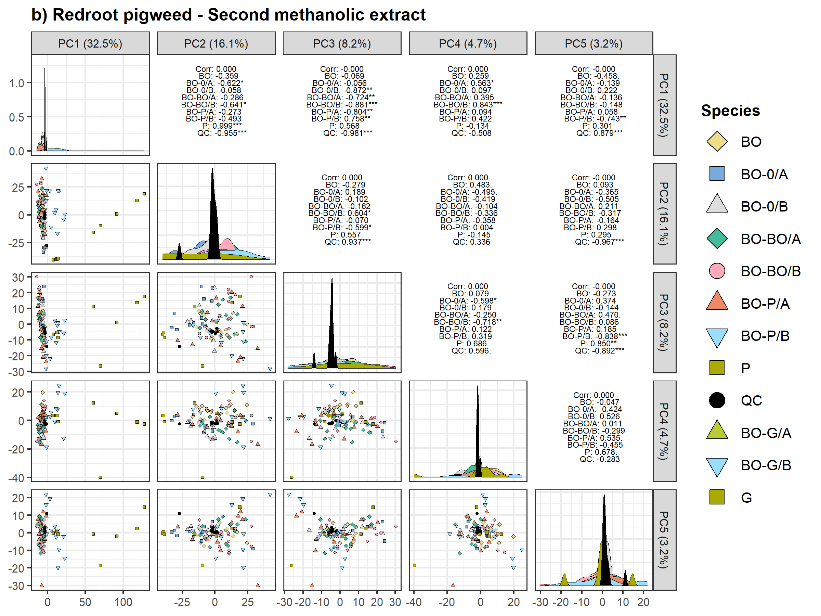

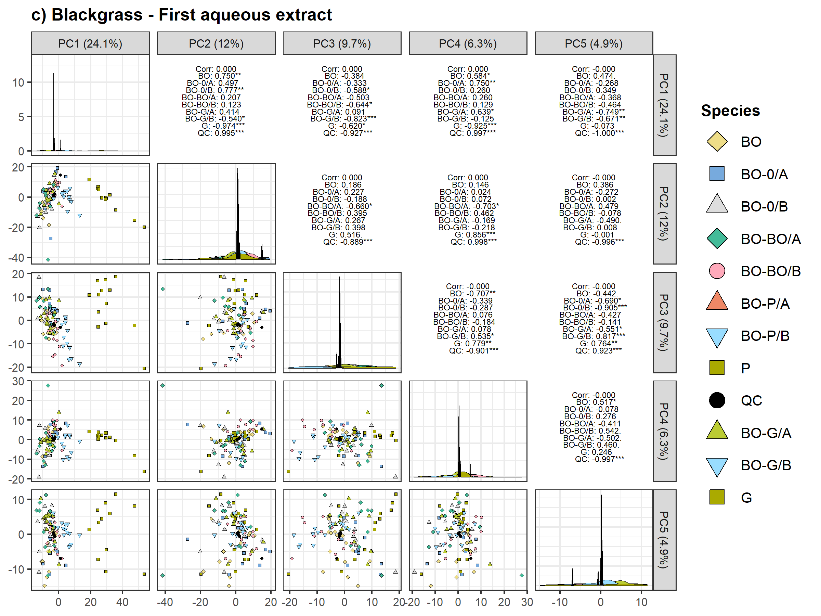

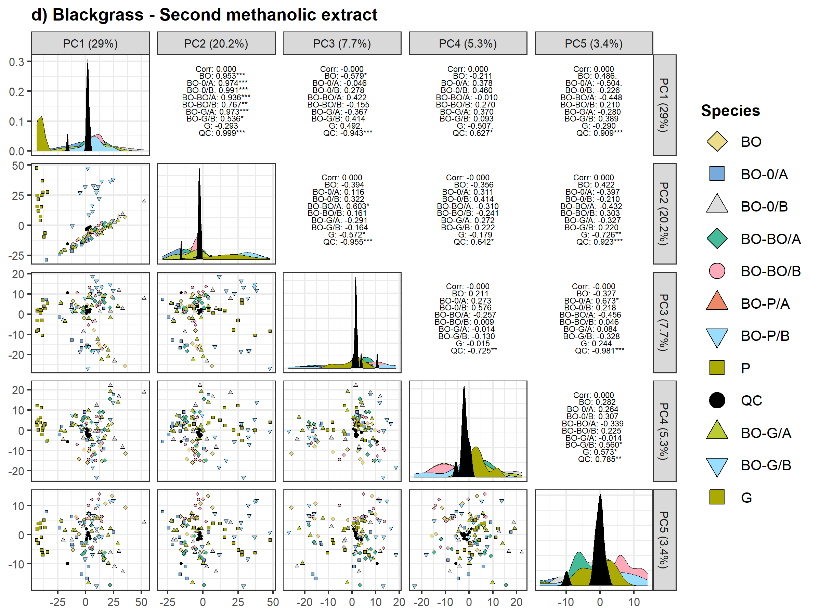


## Figure S3: PCA matrix for the first 5 components of redroot pigweed experiment aqueous extracts (a) and methanolic extracts (b) and blackgrass experiment aqueous extracts (c) and methanolic extracts (d) comparing the 3 A compartments for each experiment.


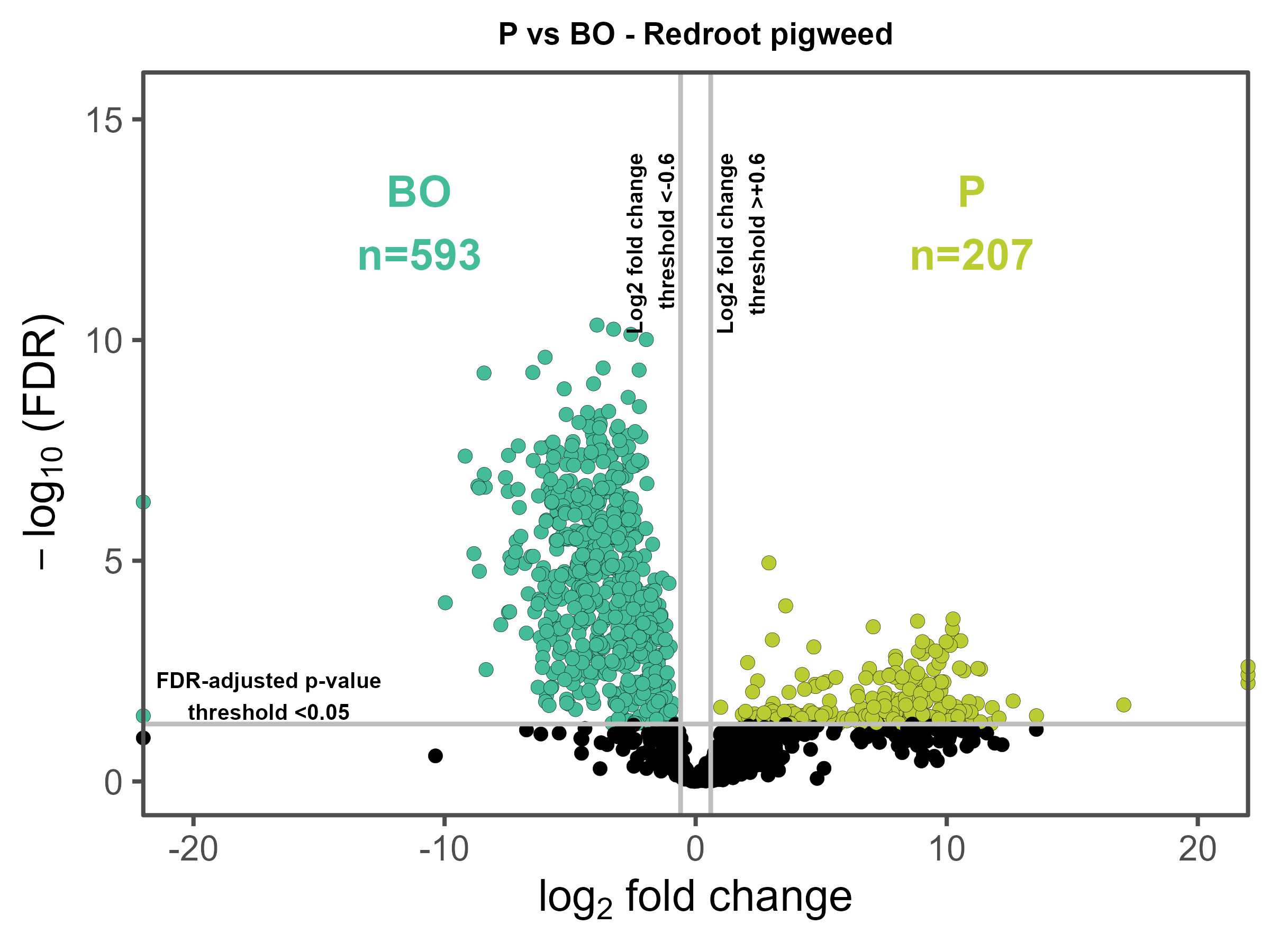


## Figure S4: Volcano plot showing differentially accumulated compounds in redroot pigweed (P) compared to black oat (BO) in the second 24-hour methanolic re-exudation extract from the redroot pigweed experimental set. Each point represents a detected compound; x-axis shows log2 fold change and y-axis shows -log10(FDR-adjusted p-value). Statistical significance was determined by Welch's t-test (FDR-corrected p < 0.05) and |log2 fold change| > 0.6.


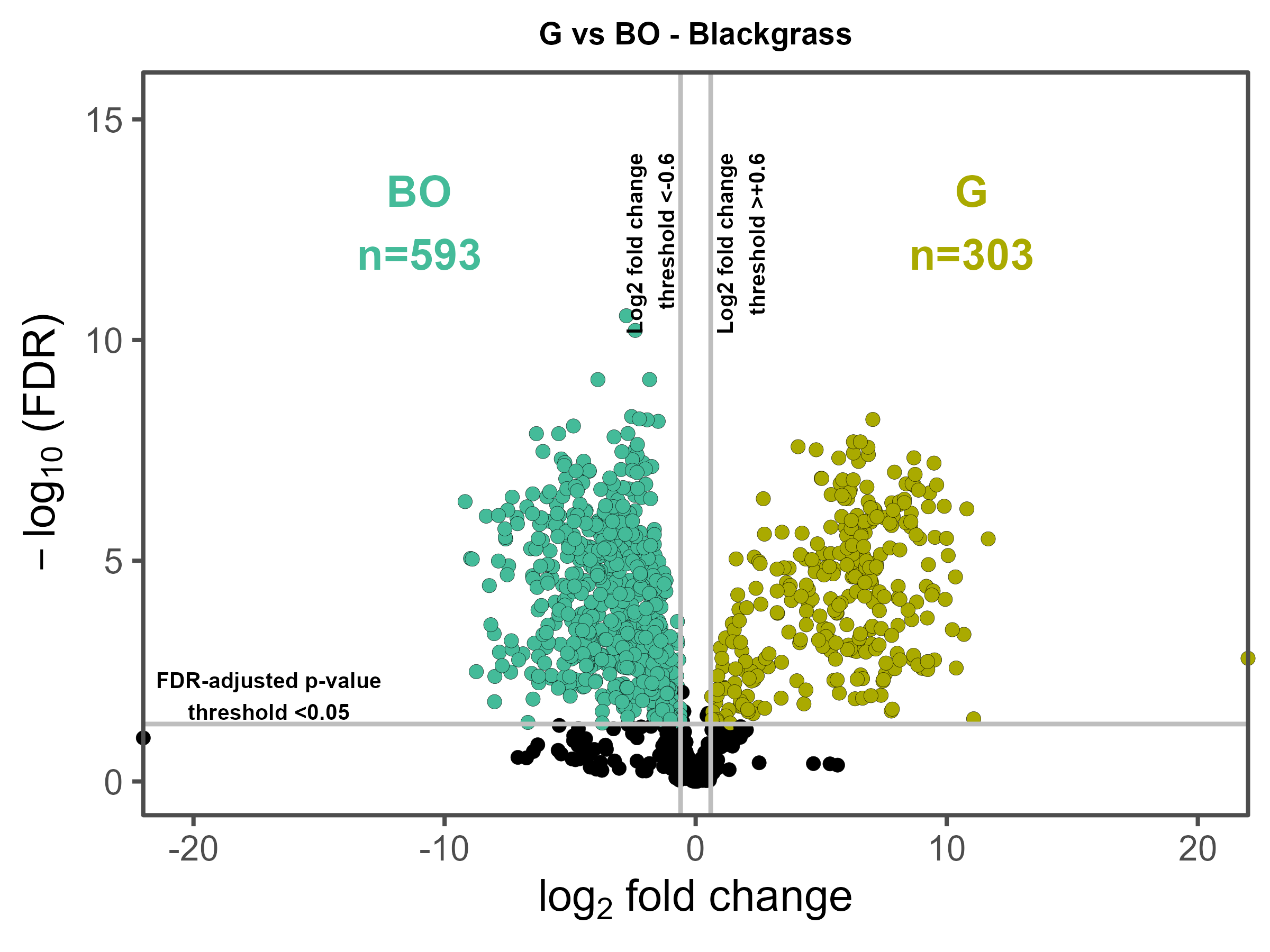


## Figure S5: Volcano plot showing differentially accumulated compounds in black grass (G) compared to black oat (BO) in the second 24-hour methanolic re-exudation extract from the black grass experimental set. Each point represents a detected compound; x-axis shows log2 fold change and y-axis shows -log10(FDR-adjusted p-value). Statistical significance was determined by Welch's t-test (FDR-corrected p < 0.05) and |log2 fold change| > 0.6.


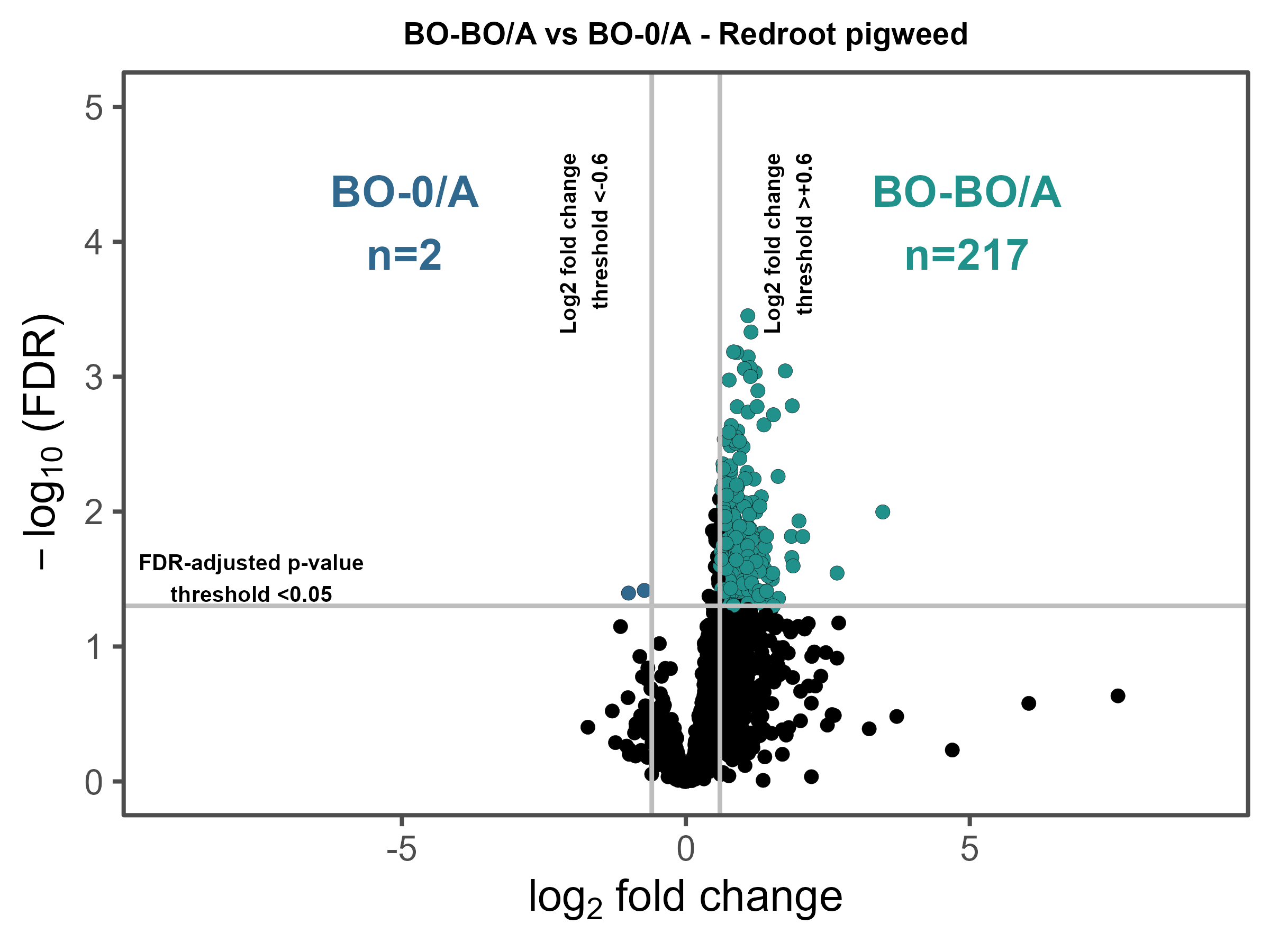


## Figure S6: Volcano plot showing differentially accumulated compounds in the A compartment of black oat grown with intraspecific neighbors (BO-BO/A) compared to black oat grown alone (BO-0/A) in the redroot pigweed experimental set. Data are from the second 24-hour methanolic re-exudation extract. Each point represents a detected compound; x-axis shows log2 fold change and y-axis shows -log10(FDR-adjusted p-value). Statistical significance was determined by Welch's t-test (FDR-corrected p < 0.05) and |log2 fold change| > 0.6.


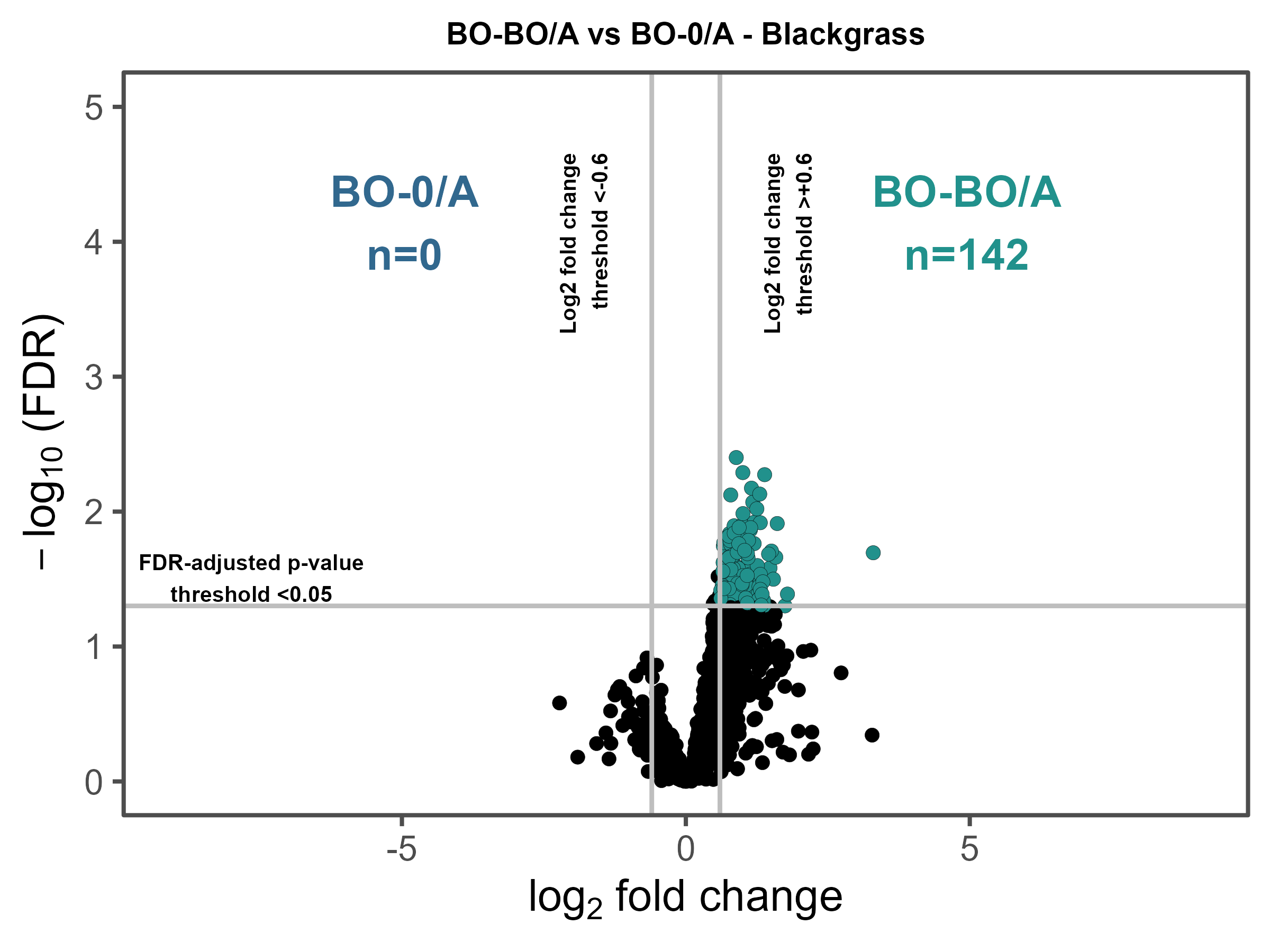


## Figure S7: Volcano plot showing differentially accumulated compounds in the A compartment of black oat grown with intraspecific neighbors (BO-BO/A) compared to black oat grown alone (BO-0/A) in the blackgrass experimental set. Data are from the second 24-hour methanolic re-exudation extract. Each point represents a detected compound; x-axis shows log2 fold change and y-axis shows -log10(FDR-adjusted p-value). Statistical significance was determined by Welch's t-test (FDR-corrected p < 0.05) and |log2 fold change| > 0.6.


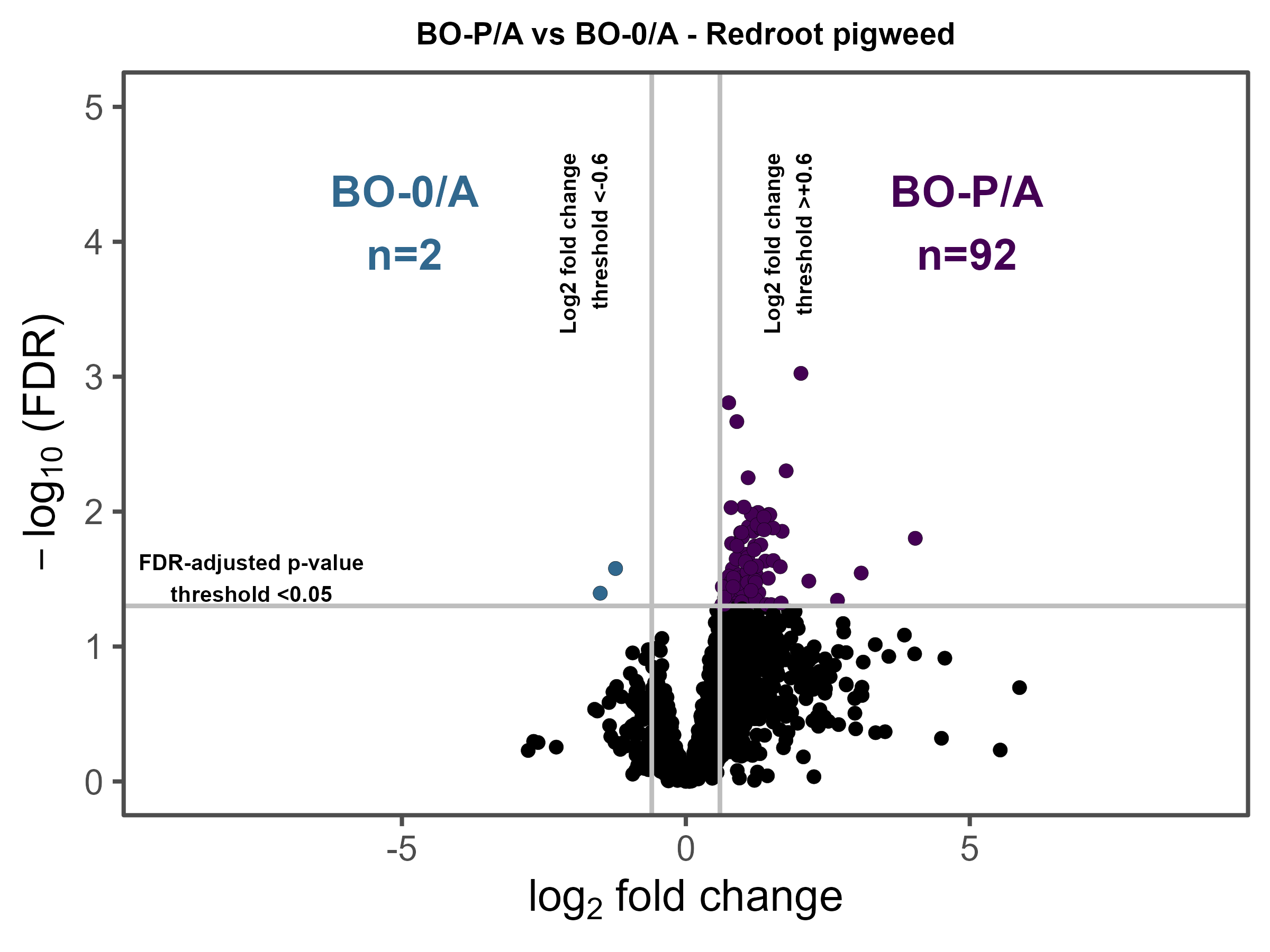


## Figure S8: Volcano plot showing differentially accumulated compounds in the A compartment of black oat grown with redroot pigweed as interspecific neighbor (BO-P/A) compared to black oat grown alone (BO-0/A) in the redroot pigweed experimental set. Data are from the second 24-hour methanolic re-exudation extract. Each point represents a detected compound; x-axis shows log2 fold change and y-axis shows -log10(FDR-adjusted p-value). Statistical significance was determined by Welch's t-test (FDR-corrected p < 0.05) and |log2 fold change| > 0.6.


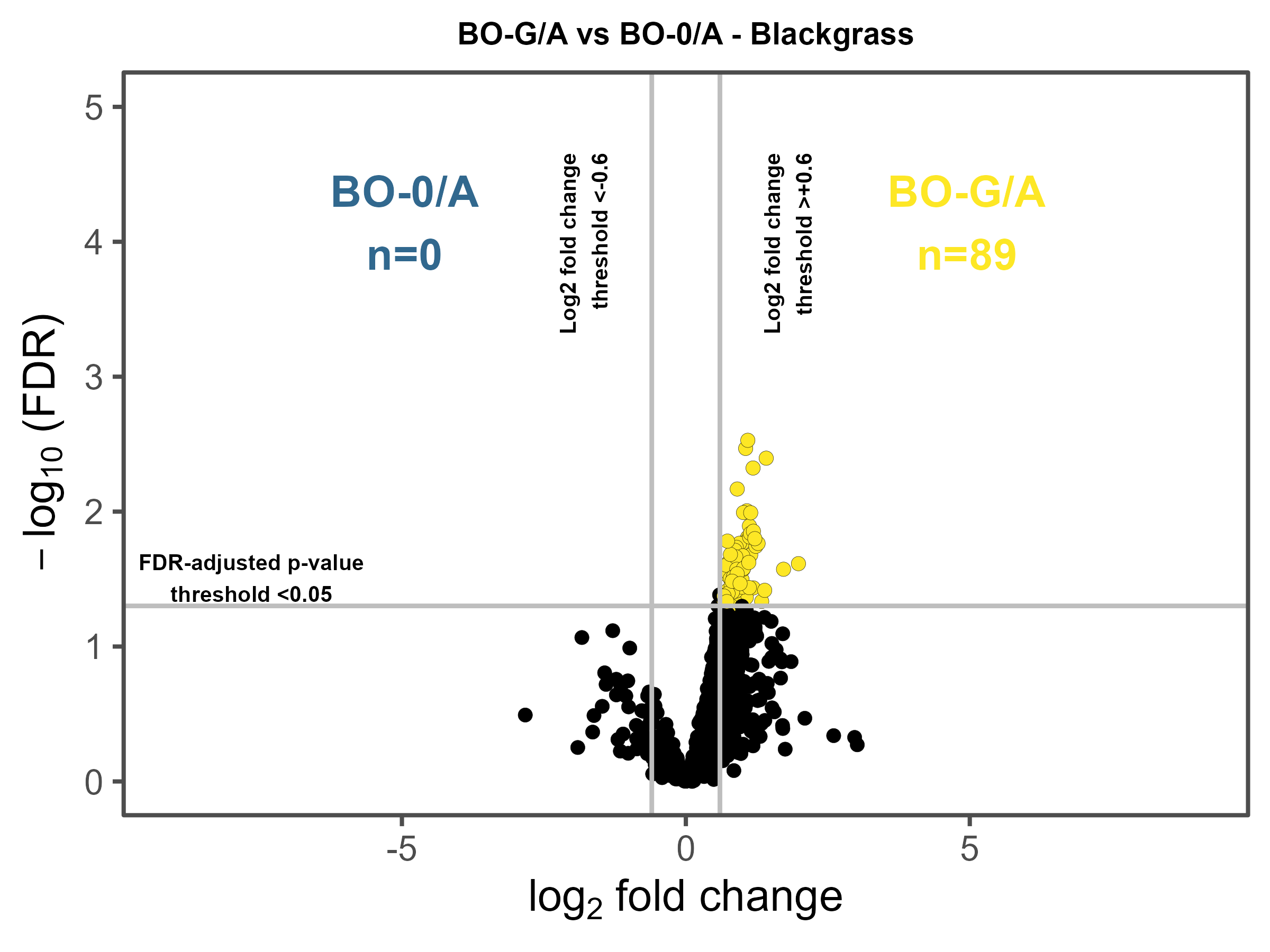


## Figure S9: Volcano plot showing differentially accumulated compounds in the A compartment of black oat grown with black grass as interspecific neighbor (BO-G/A) compared to black oat grown alone (BO-0/A) in the blackgrass experimental set. Data are from the second 24-hour methanolic re-exudation extract. Each point represents a detected compound; x-axis shows log2 fold change and y-axis shows -log10(FDR-adjusted p-value). Statistical significance was determined by Welch's t-test (FDR-corrected p < 0.05) and |log2 fold change| > 0.6.


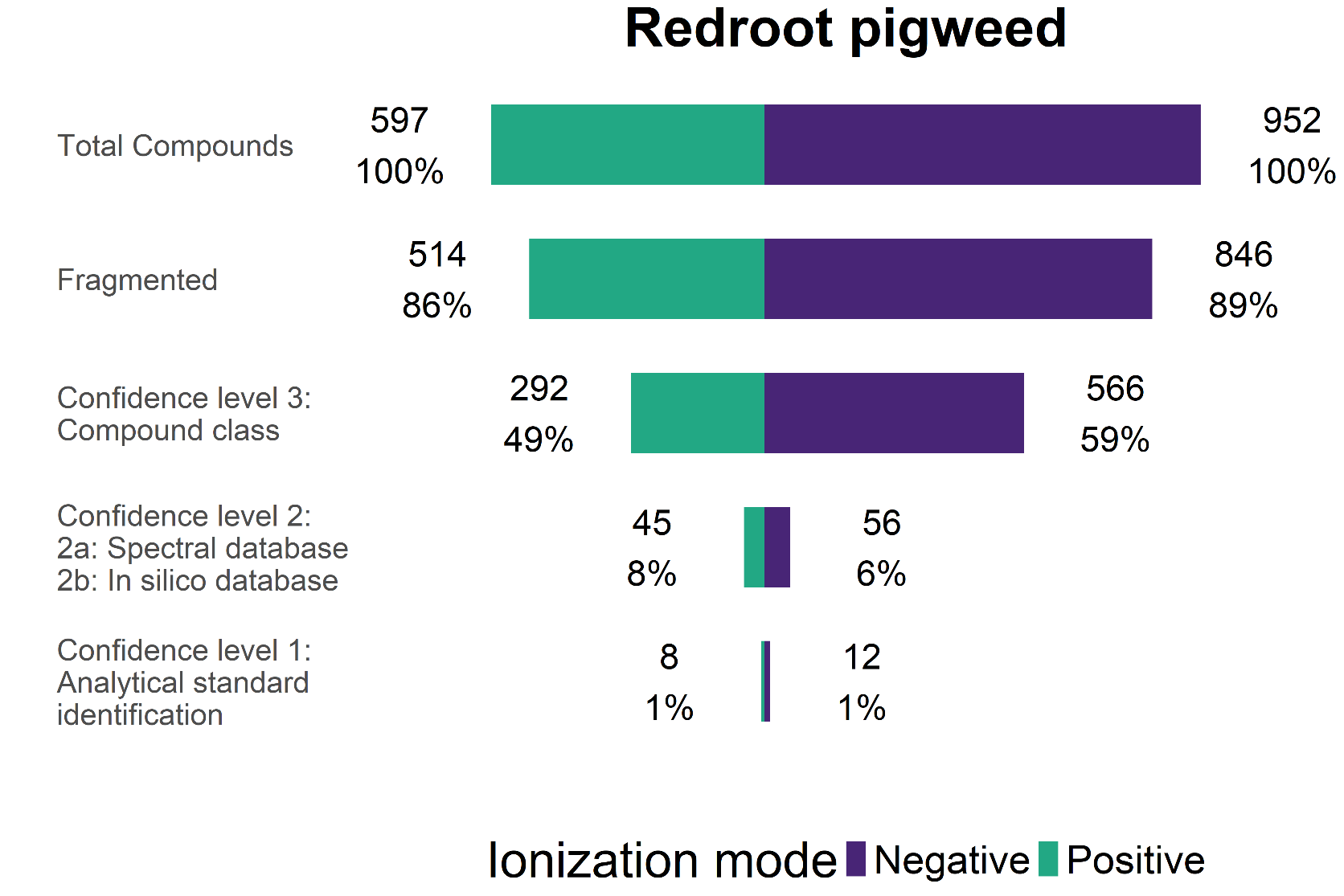


## Figure S10: Compounds were identified to different levels of confidence based on the Schymanski scale for the P experimental set from the second methanolic re-exudation extract. For confidence levels 1-3, a fragmentation pattern (MS2) is necessary for identification. Identification level 3, compound class identification, was annotated using the CANOPUS application of the SIRIUS open-source software. Identification level 2a was annotated with MS FINDER and GNPS using all available open-source libraries. 2b was annotated with MS FINDER and SIRIUS [CSI: FingerID](https://en.wikipedia.org/wiki/SIRIUS_(software)#CSI:FingerID:_Structure_database_search). Confidence level 1 was annotated with an in-house database generated from a set of authentic analytical standards of compounds known to be exudated by the roots of crops. Negative ionization mode data is shown in purple and positive ionization mode is teal.


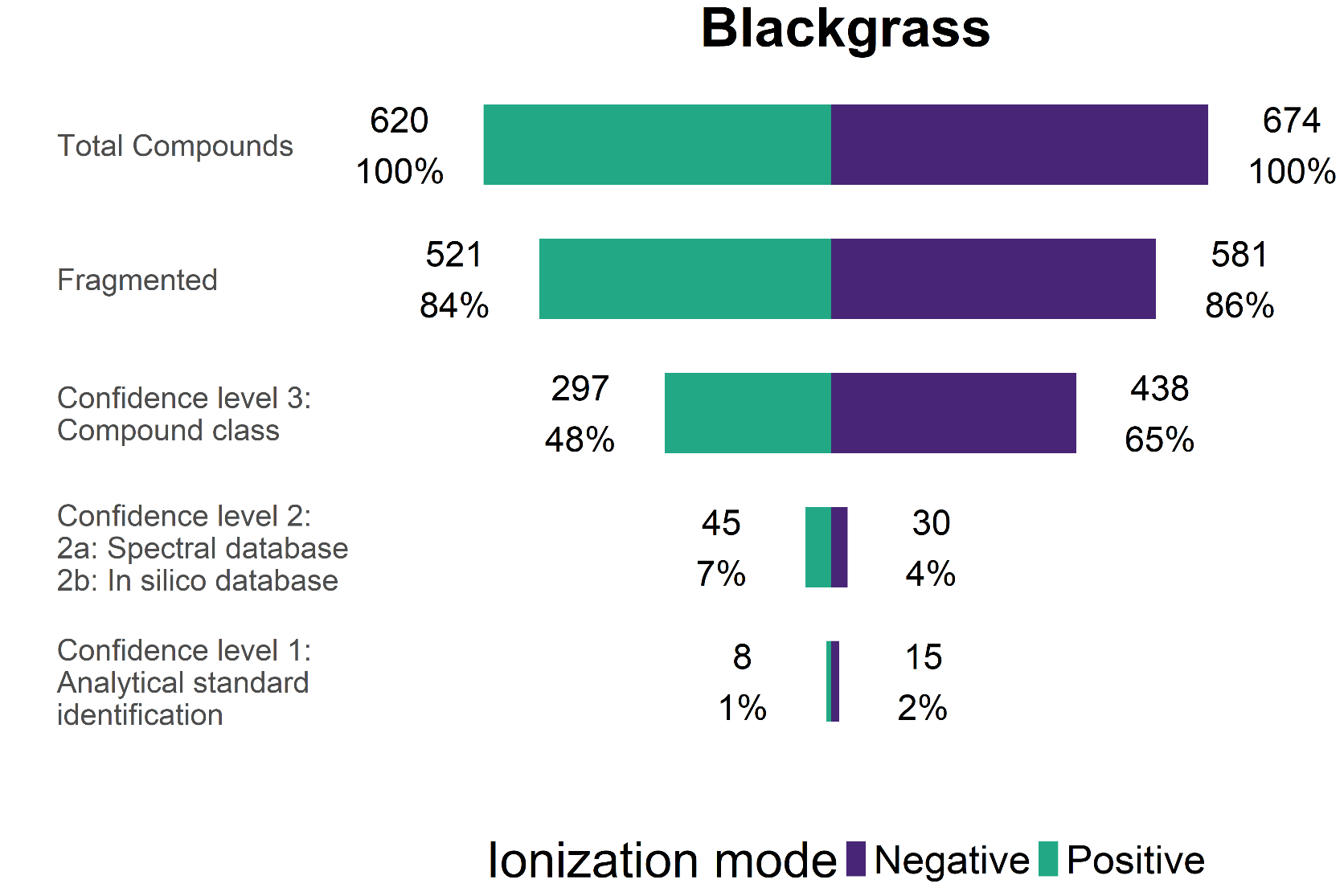


## Figure S11: Compounds were identified to different levels of confidence based on the Schymanski scale for the G experimental set from the second methanolic re-exudation extract. For confidence levels 1-3, a fragmentation pattern (MS2) is necessary for identification. Identification level 3, compound class identification, was annotated using the CANOPUS application of the SIRIUS open-source software. Identification level 2a was annotated with MS FINDER and GNPS using all available open-source libraries. 2b was annotated with MS FINDER and SIRIUS [CSI: FingerID](https://en.wikipedia.org/wiki/SIRIUS_(software)#CSI:FingerID:_Structure_database_search). Confidence level 1 was annotated with an in-house database generated from a set of authentic analytical standards of compounds known to be exudated by the roots of crops. Negative ionization mode data is shown in purple and positive ionization mode is teal.


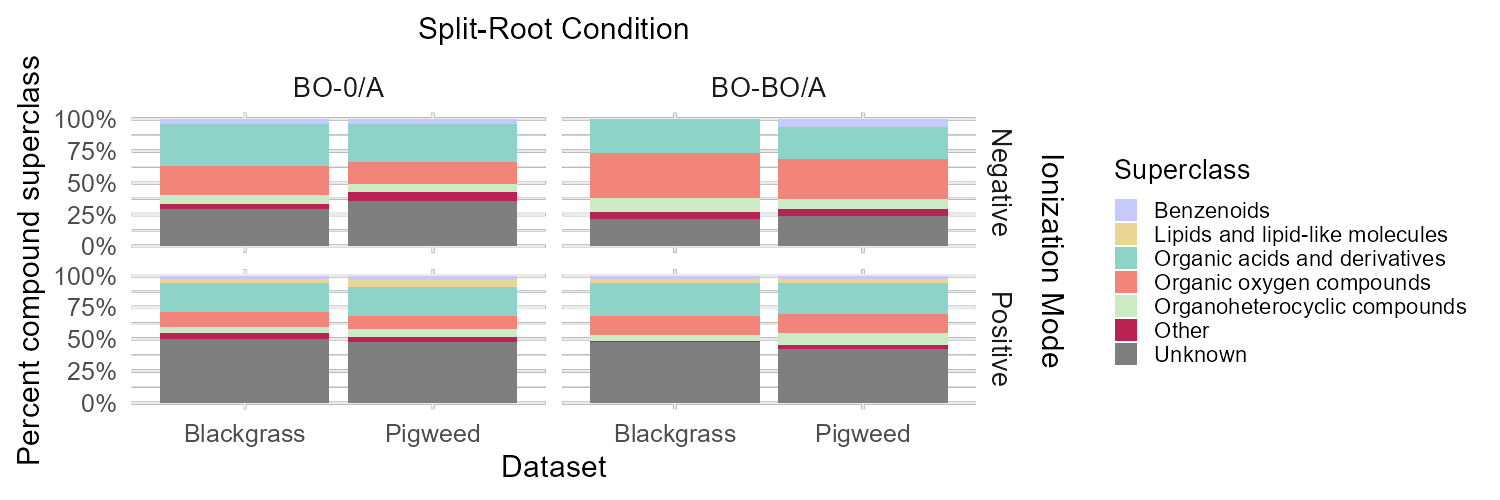


## Figure S12: Metabolite superclass distribution is consistent across independent experimental datasets. Stacked bar charts show the distribution of metabolite superclasses for two treatment comparisons shared between the blackgrass and pigweed experimental datasets. BO-0/A represents the A compartment of a split-root setup where black oat was grown alone with no neighbor in the B compartment. Compounds shown for black oat alone were those not differentially accumulated in redroot pigweed or blackgrass (i.e., not significantly different between black oat and either weed species), as determined by Welch's t-test (FDR-corrected p < 0.05) and |log2 fold change| > 0.6. BO-BO/A represents the A compartment where black oat was grown with an intraspecific neighbor (another black oat) in the B compartment. Compounds that were more accumulated by the presence of an intraspecific neighbor (BO-BO/A) were those which were significantly higher than BO-0/A using these same thresholds. Rows represent ionization modes used for LC-MS analysis (negative and positive). Compounds with significantly different accumulation between conditions were determined by Welch's t-test (FDR-corrected p < 0.05) and |log₂ fold change| > 0.6. The similar distribution of compound superclasses across datasets demonstrates reproducibility between independent experiments. Metabolites were classified using CANOPUS application of SIRIUS. Compound superclasses which comprised less than 5% of the total number of compounds for that ionization mode were binned together into the "other" category.


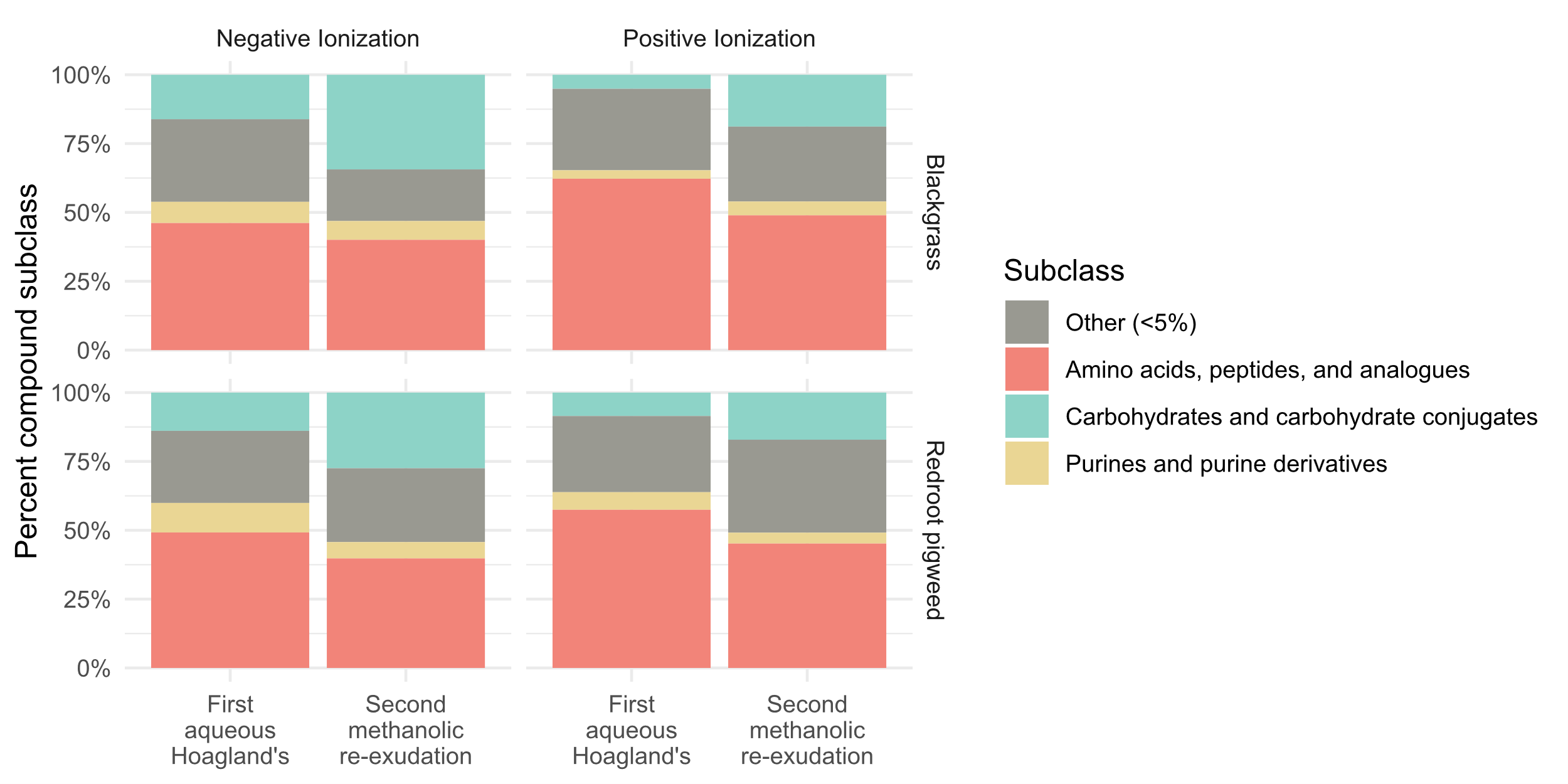


## Figure S12: Analysis from the CANOPUS function in SIRIUS which identified all possible subclasses of compounds from all sample types exported from MS DIAL compares the first aqueous two-week extraction of Hoagland’s solution to the second methanolic 24 hour re-exudation extraction. This is shown for both the P experimental set and the G experimental set in negative and positive ionization mode. Only compounds whose subclass could confidently be annotated were considered by applying a 90% probability score cutoff for the compound subclass identification. Any identification below this threshold were considered unknowns and were thus excluded. Compounds which were of a subclass which comprised less than 5% of the total number of identifiable compounds were binned into an “Other” category.


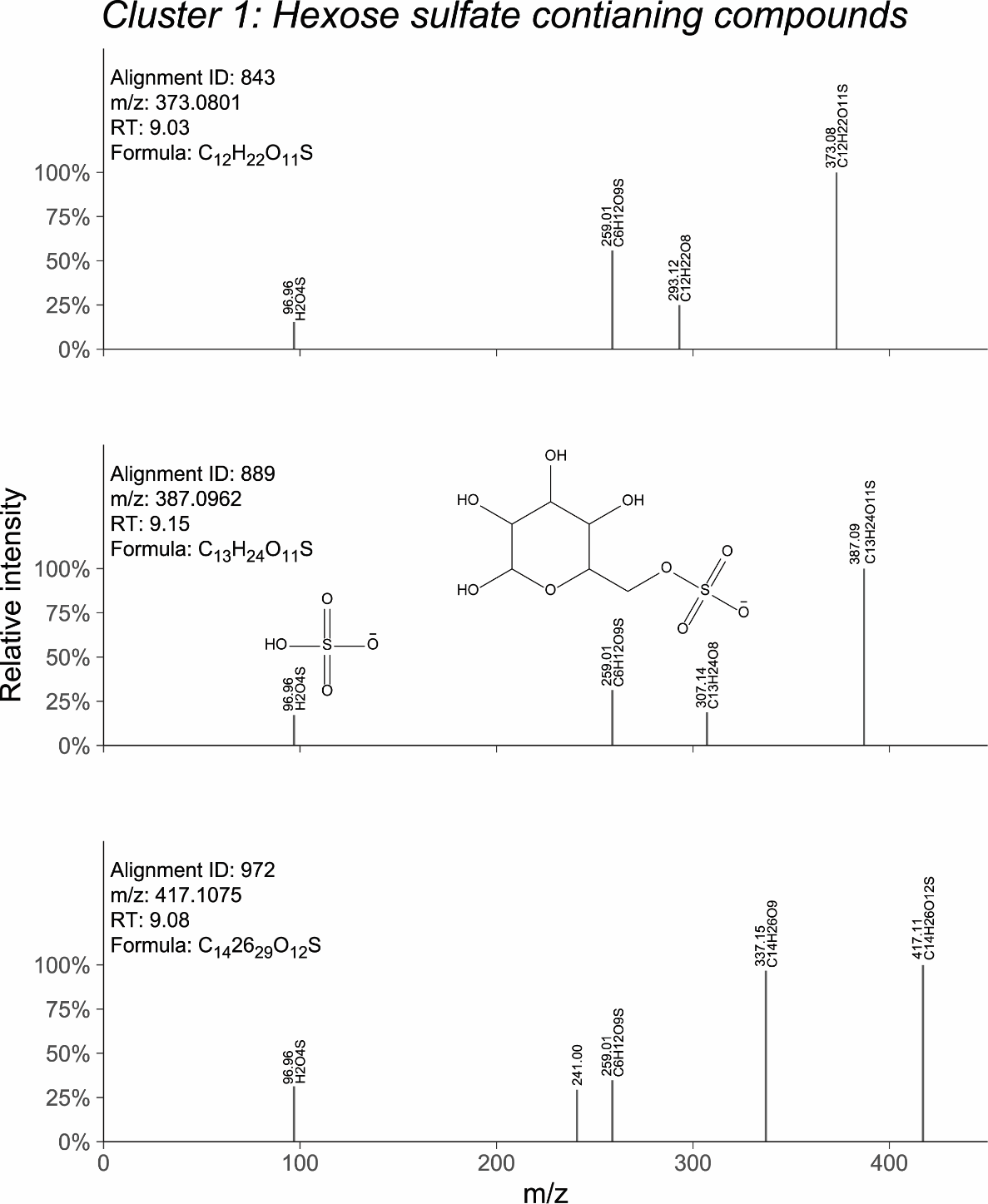


## Figure S14: Fragmentation patterns for aligned compounds of interest in cluster 1 of the molecular network highlighting the sulphate (96.96 M/z) and sulphate + hexose (259.01 M/z) fragments and SO_3_ loss (79.96 Da).


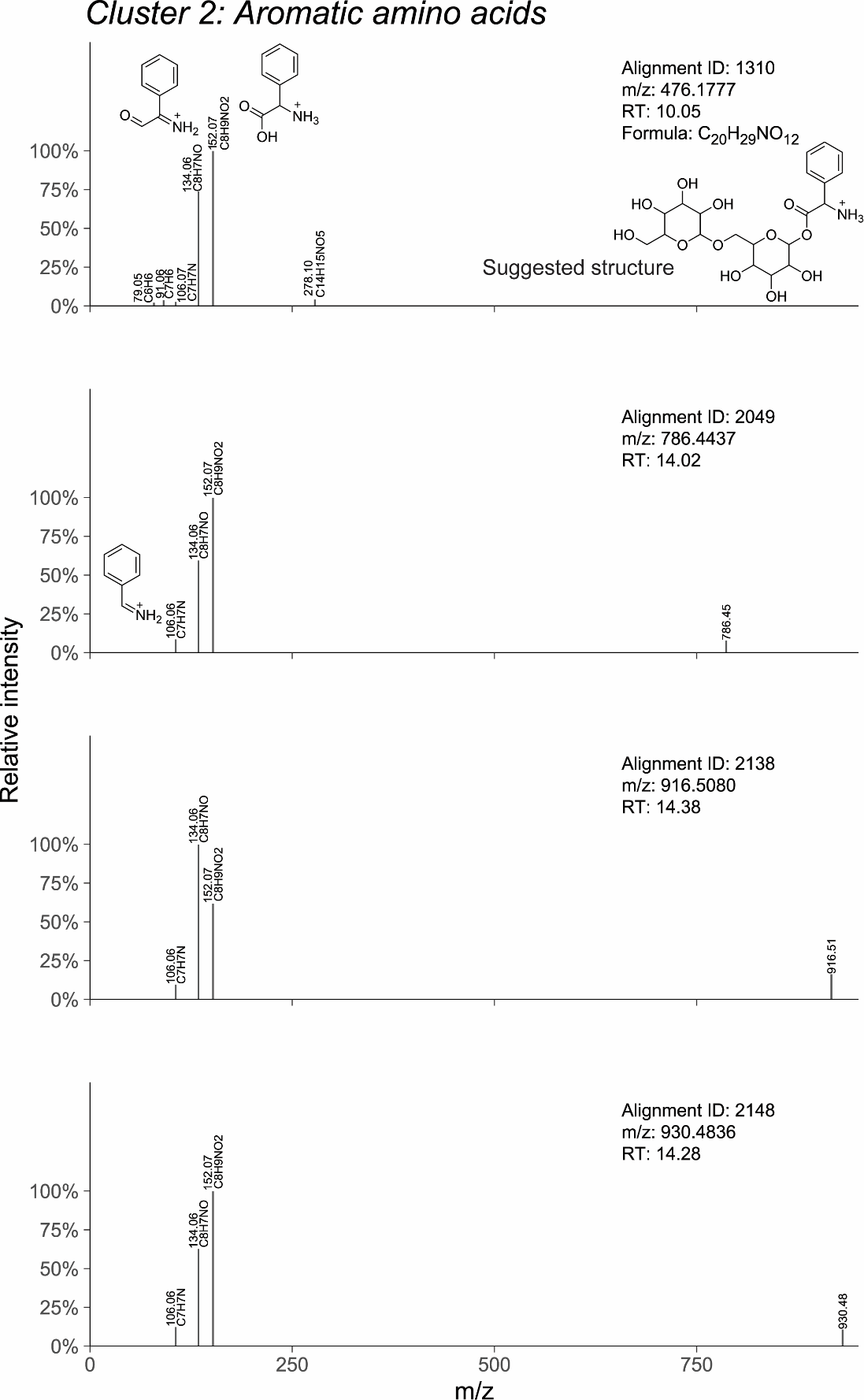


## **Figure S15:** Fragmentation patterns for aligned compounds of interest in cluster 2 of the molecular network highlighting Alignment ID 1310 is likely comprised of a benzene + amino (106.06 M/z) + carboxyl (152.07 M/z) + dihexose (476.18 M/z). All highlighted aligned compounds contain benzene, carboxyl, and amino-indicating fragments.


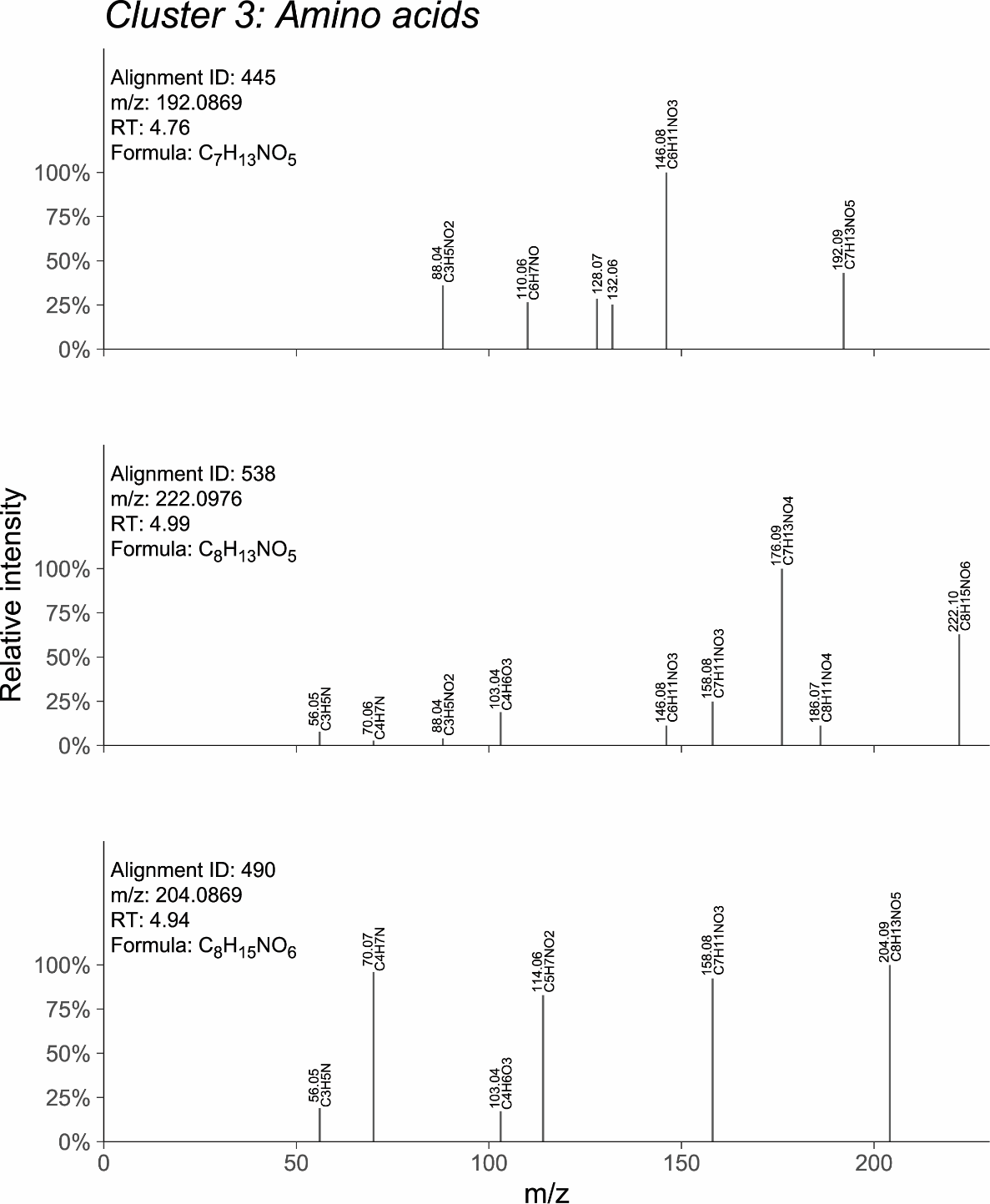


## Figure S16: Fragmentation patterns for aligned compounds of interest in cluster 3 of the molecular network which share a carboxylic acid (46.01 Da) functional group loss within the fragmentation pattern.


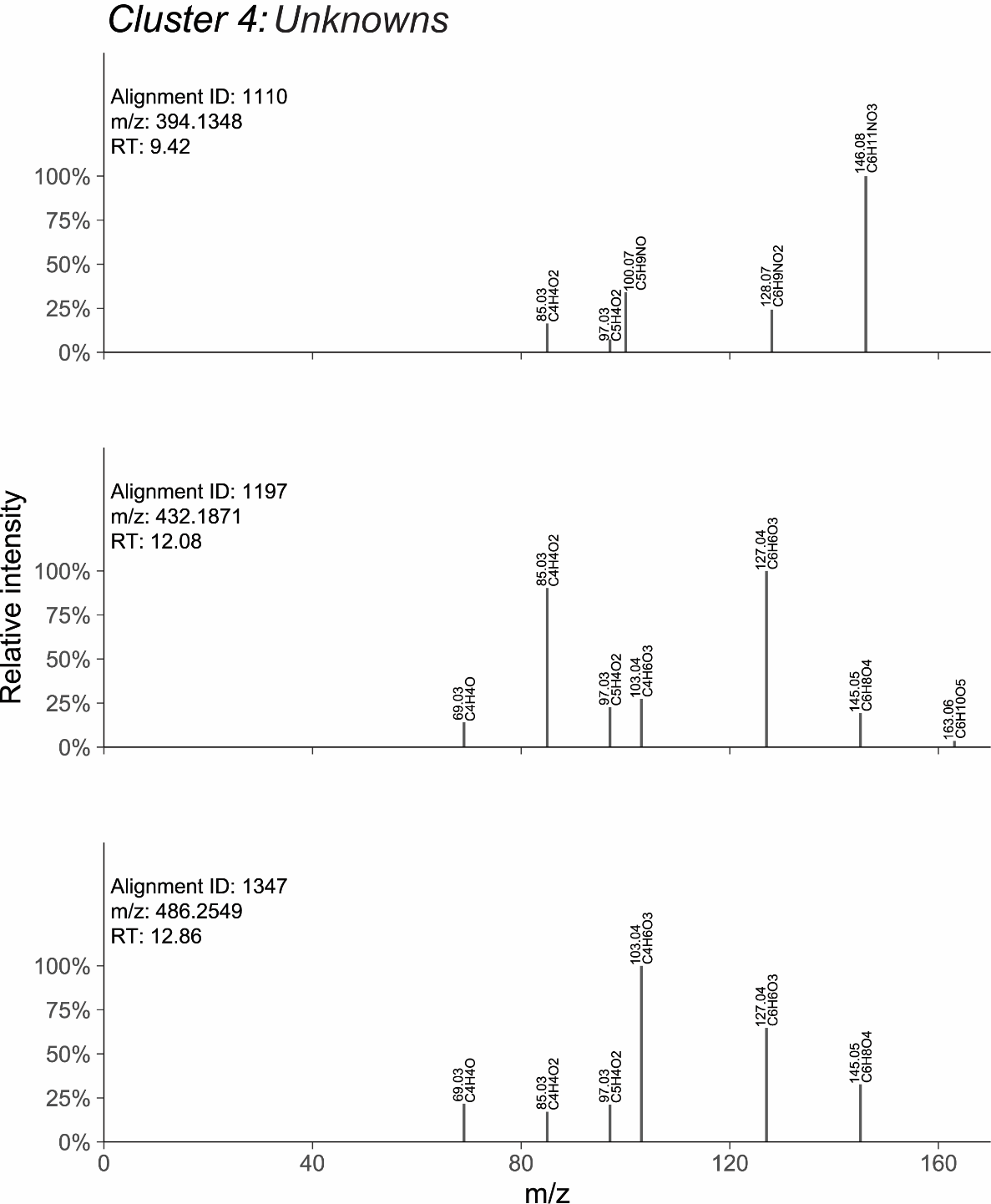


## Figure S17: Fragmentation patterns for aligned compounds of interest in cluster 4.

# REFERENCES

[1] J.M. Chaparro, D.V. Badri, M.G. Bakker, A. Sugiyama, D.K. Manter, J.M. Vivanco, Root Exudation of Phytochemicals in Arabidopsis Follows Specific Patterns That Are Developmentally Programmed and Correlate with Soil Microbial Functions, PLOS ONE 8 (2013) e55731. https://doi.org/10.1371/journal.pone.0055731.

[2] G. Neumann, S. Bott, M.A. Ohler, H.-P. Mock, R. Lippmann, R. Grosch, K. Smalla, Root exudation and root development of lettuce (Lactuca sativa L. cv. Tizian) as affected by different soils, Front. Microbiol. 5 (2014). https://doi.org/10.3389/fmicb.2014.00002.

[3] A.D. Rovira, Plant root exudates, The Botanical Review 35 (1969) 35–57. https://doi.org/10.1007/BF02859887.

[4] A. Kawasaki, S. Donn, P.R. Ryan, U. Mathesius, R. Devilla, A. Jones, M. Watt, Microbiome and Exudates of the Root and Rhizosphere of Brachypodium distachyon, a Model for Wheat, PLOS ONE 11 (2016) e0164533. https://doi.org/10.1371/journal.pone.0164533.

[5] L.C. Carvalhais, P.G. Dennis, D. Fedoseyenko, M. Hajirezaei, R. Borriss, N. von Wirén, Root exudation of sugars, amino acids, and organic acids by maize as affected by nitrogen, phosphorus, potassium, and iron deficiency, Z. Pflanzenernähr. Bodenk. 174 (2011) 3–11. https://doi.org/10.1002/jpln.201000085.

[6] A.I. Shaposhnikov, V.Yu. Shakhnazarova, N.A. Vishnevskaya, E.V. Borodina, O.K. Strunnikova, Aromatic Carboxylic Acids in Barley-Root Exudates and Their Influence on the Growth of Fusarium culmorum and Pseudomonas fluorescens, Appl Biochem Microbiol 56 (2020) 344–351. https://doi.org/10.1134/S0003683820030138.

[7] S.B. Miller, A.L. Heuberger, C.D. Broeckling, C.E. Jahn, Non-Targeted Metabolomics Reveals Sorghum Rhizosphere-Associated Exudates are Influenced by the Belowground Interaction of Substrate and Sorghum Genotype, International Journal of Molecular Sciences 20 (2019) 431. https://doi.org/10.3390/ijms20020431.

[8] B.K. Ghimire, B. Ghimire, C.Y. Yu, I.-M. Chung, Allelopathic and Autotoxic Effects of Medicago sativa—Derived Allelochemicals, Plants 8 (2019) 233. https://doi.org/10.3390/plants8070233.

[9] C.-H. Kong, S.-Z. Zhang, Y.-H. Li, Z.-C. Xia, X.-F. Yang, S.J. Meiners, P. Wang, Plant neighbor detection and allelochemical response are driven by root-secreted signaling chemicals, Nat Commun 9 (2018) 3867. https://doi.org/10.1038/s41467-018-06429-1.

[10] V. Novak, P.F. Andeer, B.P. Bowen, Y. Ding, K. Zhalnina, C. Tomaka, A.N. Golini, S.M. Kosina, T.R. Northen, Reproducible growth of Brachypodium distachyon in fabricated ecosystems (EcoFAB 2.0) reveals that nitrogen form and starvation modulate root exudation, Plant Biology, 2023. https://doi.org/10.1101/2023.01.18.524647.

[11] K. Tawaraya, R. Horie, A. Saito, T. Shinano, T. Wagatsuma, K. Saito, A. Oikawa, Metabolite profiling of shoot extracts, root extracts, and root exudates of rice plant under phosphorus deficiency, Journal of Plant Nutrition 36 (2013) 1138–1159. https://doi.org/10.1080/01904167.2013.780613.

[12] D.R. Batish, S. Kaur, H.P. Singh, R.K. Kohli, Nature of interference potential of leaf debris of Ageratum conyzoides, Plant Growth Regul 57 (2008) 137–144. https://doi.org/10.1007/s10725-008-9329-9.

[13] K. Sasikumar, C. Vijayalakshmi, K.T. Parthiban, Allelopathic effects of four eucalyptus species on redgram (Cajanus cajan L.), Journal of Tropical Agriculture 39 (2001) 134–138.

[14] M.A.K. Lodhi, R. Bilal, K.A. Malik, Allelopathy in agroecosystems: Wheat phytotoxicity and its possible roles in crop rotation, J Chem Ecol 13 (1987) 1881–1891. https://doi.org/10.1007/BF01013237.

[15] A. Iannucci, M. Fragasso, C. Platani, R. Papa, Plant growth and phenolic compounds in the rhizosphere soil of wild oat (Avena fatua L.), Frontiers in Plant Science 4 (2013). https://www.frontiersin.org/articles/10.3389/fpls.2013.00509 (accessed November 20, 2023).

[16] J. Kalinova, N. Vrchotova, J. Triska, Exudation of Allelopathic Substances in Buckwheat Fagopyrum esculentum, Journal of Agricultural and Food Chemistry 55 (2007) 6453–6459. https://doi.org/10.1021/jf070795u.

[17] Y.K. Kim, X. Li, H. Xu, N. Il Park, M.R. Uddin, J.Y. Pyon, S.U. Park, Production of phenolic compounds in hairy root culture of tartary buckwheat (Fagopyrum tataricum Gaertn), J. Crop Sci. Biotechnol. 12 (2009) 53–57. https://doi.org/10.1007/s12892-009-0075-y.

[18] A. Gfeller, G. Glauser, C. Etter, C. Signarbieux, J. Wirth, Fagopyrum esculentum Alters Its Root Exudation after Amaranthus retroflexus Recognition and Suppresses Weed Growth, Front. Plant Sci. 9 (2018). https://doi.org/10.3389/fpls.2018.00050.

[19] E. Tsuzuki, Y. Yamamoto, T. Shimizu, Fatty Acids in Buckwheat are Growth Inhibitors, Annals of Botany 60 (1987) 69–70. https://doi.org/10.1093/oxfordjournals.aob.a087423.

[20] J. Kalinova, N. Vrchotova, Level of Catechin, Myricetin, Quercetin and Isoquercitrin in Buckwheat ( *Fagopyrum esculentum* Moench), Changes of Their Levels during Vegetation and Their Effect on The Growth of Selected Weeds, Journal of Agricultural and Food Chemistry 57 (2009) 2719–2725. https://doi.org/10.1021/jf803633f.
